# Tracking mental health and cognitive changes following the Australian social media minimum age legislation: a 4-wave longitudinal study protocol

**DOI:** 10.1136/bmjopen-2026-120216

**Published:** 2026-08-05

**Authors:** Anna Bilston, Lyndsay Brown, Xu Qi, Emily Morgan, Yasmin Hasan, Sarah Stevens, Weike Wang, Aliza Werner-Seidler, Susanne Schweizer

**Affiliations:** 1 School of Psychology, University of New South Wales, Sydney, New South Wales, Australia; 2 Black Dog Institute, University of New South Wales, Sydney, New South Wales, Australia

**Keywords:** Social Media, Child & adolescent psychiatry, Anxiety disorders, Depression & mood disorders, Parents, PUBLIC HEALTH

## Abstract

**Abstract:**

**Introduction:**

Over the last two decades, increases in adolescent mental health difficulties have been recorded alongside a rapid expansion in social media use. In response to these trends, on 10 December 2025, the Australian government implemented Social Media Minimum Age legislation prohibiting Australians under the age of 16 years from having social media accounts. The introduction of this legislation allows for a unique quasi-experimental investigation of the impact of social media use on adolescent mental health and the mechanisms through which social media may impact young Australian adolescents’ mental health, well-being and cognition.

**Methods and analysis:**

To assess the relationship between social media use and young adolescents’ mental health, well-being and cognition, a longitudinal mixed-methods approach will be taken. 422 young people (aged 10–15 years) and parent/carer dyads were recruited and invited to complete a baseline assessment before the implementation of the Social Media Minimum Age legislation (October–December 2025). Three follow-up assessments will then be completed after 6 (April–May 2026), 12 (October–November 2026) and 18 months (April–May 2027). Data from parent/carer and adolescent surveys, ecological momentary assessments (EMAs), cognitive tasks and smartphone-recorded screenshots of social media and messaging app use across each of these timepoints will be analysed. Linear regression analyses will be used to assess correlational relationships between social media experiences and responses and mental health outcomes as well as how individual factors moderate these relationships. To assess these relationships over time and between momentary experiences and responses (EMA data) and more stable trait-based (individual) and environmental (parenting and government restrictions) factors, we will use linear mixed-effects models and mixed-effects segmented regression.

**Ethics and dissemination:**

This study has been approved by the UNSW Human Research Ethics Committee (iRECS 9197). Findings from this study will help identify potential opportunities for interventions at individual, micro (parental) and macro (governmental) levels to mitigate any negative impacts of social media for younger adolescents. Results will be disseminated through peer-reviewed publications, conference presentations, public engagement opportunities and researcher-associated websites. After each assessment timepoint, core findings will also be distilled into engaging formats such as infographics and videos, which will be shared with young people and their parent/carer.

**STRENGTHS AND LIMITATIONS OF THIS STUDY**

- Ecological momentary assessments (EMAs) to capture mood, mental health symptoms and online exposures in close-to-real time, minimising recall bias, combined with a longitudinal design to capture changes both within and between participants across a 5-year age range, enabling investigation of the causal role of social media exposure on mental health by age;
- Matched parent/carer and adolescent dyads to assess associations with digital parenting as well as data collection before and ~6, 12 and 18 months after the introduction of the Social Media Minimum Age legislation, enabling examination of the potential impacts of the Australian government policy.
- In contrast, potential limitations include limited generalisability due to under-representation of lower socio-economic groups, diverse genders and Aboriginal and Torres Strait Islander Peoples;
- The risk of insufficient participant retention to maintain statistical power for all analyses, albeit minimised by engaging participant communications and remuneration, increases across each stage of the study;
- EMAs are limited by not capturing interactions before and during the school day when participants are not permitted access to their mobile phones;

## Introduction

 Globally, increases in adolescent social media use have occurred alongside rising rates of adolescent psychological distress and mental health problems, prompting debate about the potentially negative impact of social media on healthy adolescent development.^1 2^ However, to date, studies examining the relationship between the time adolescents spend on social media and their mental health and cognitive functioning have produced mixed results, highlighting the need to better understand the factors that may explain why social media appears to be harmful for some, but not other, young people.^3–5^ One way of conceptualising these differences is to consider influences on adolescent social media usage, behaviour and responses at multiple levels—individual, micro (parental) and macro (governmental).

At the individual level, dispositional and developmental factors, such as social sensitivity and propensity to social comparison, are likely to shape adolescents’ responses to social media and may moderate the relationship between these responses and mental health and cognition.^6–11^ These factors may also differentially influence how adolescents respond to experiences that mediate negative social media effects, such as cyber-bullying and exposure to sexual, hateful or violent content.^12^

At a micro level, adolescents’ online exposure and experiences are likely to be influenced by parents’ positive or negative views of social media, their attitude to the acceptable amount and type of social media exposure for their children and themselves and the way they negotiate social media access with their children, often referred to as ‘digital parenting’.^13–16^ Digital parenting styles may also impact mental health outcomes, with emerging research suggesting stronger positive associations between positive adolescent mental health and open communication and monitoring than with restrictions and surveillance.^17 18^ Finally, at the macro level, societal rules and norms shape adolescents’ access to and use of social media platforms. The recently implemented *Online Safety Amendment (Social Media Minimum Age) Act 2024* places restrictions on adolescents under 16 years of age from holding a social media account. The introduction of the restrictions provides a novel opportunity for a quasi-experimental investigation of how the broader regulatory environments may influence adolescents’ online experiences by comparing adolescent social media experiences and mental health before and after implementation of the legislation.^19 20^

Elucidating the role of factors operating at each of these levels of influence is critical to determine (1) whether and how social media use is related to adolescent mental health issues, (2) whether social media use impacts adolescent cognitive development and (3) if so, where and how we might optimally intervene—at an individual, parental and/or governmental level—to help prevent further declines in adolescent well-being and the associated human and economic costs.

### Aims of the study

This study aims to expand our understanding of the relationship between social media and young adolescents’ (10–15-year-olds)^21^; mental health, well-being and cognition over an 18-month time frame and to identify the mechanisms through which these relationships may operate. Specifically, we aim to:

1. Examine the relationships between adolescents’ mental health, well-being and cognition and (a) objectively measured time spent on social media and (b) online versus offline momentary social experiences and responses;
2. Examine the moderating role of dispositional and developmental differences on these relationships over time;
3. Investigate the role of digital parenting in modifying the impact of social media use on adolescent mental health and well-being, both between different individuals and within the same individuals over time;
4. Assess the impact of the Australian Social Media Minimum Age legislation on adolescents’ social media usage and their mental health, well-being and cognition.

## Methods and analysis

### Study design

**Figure 1:**
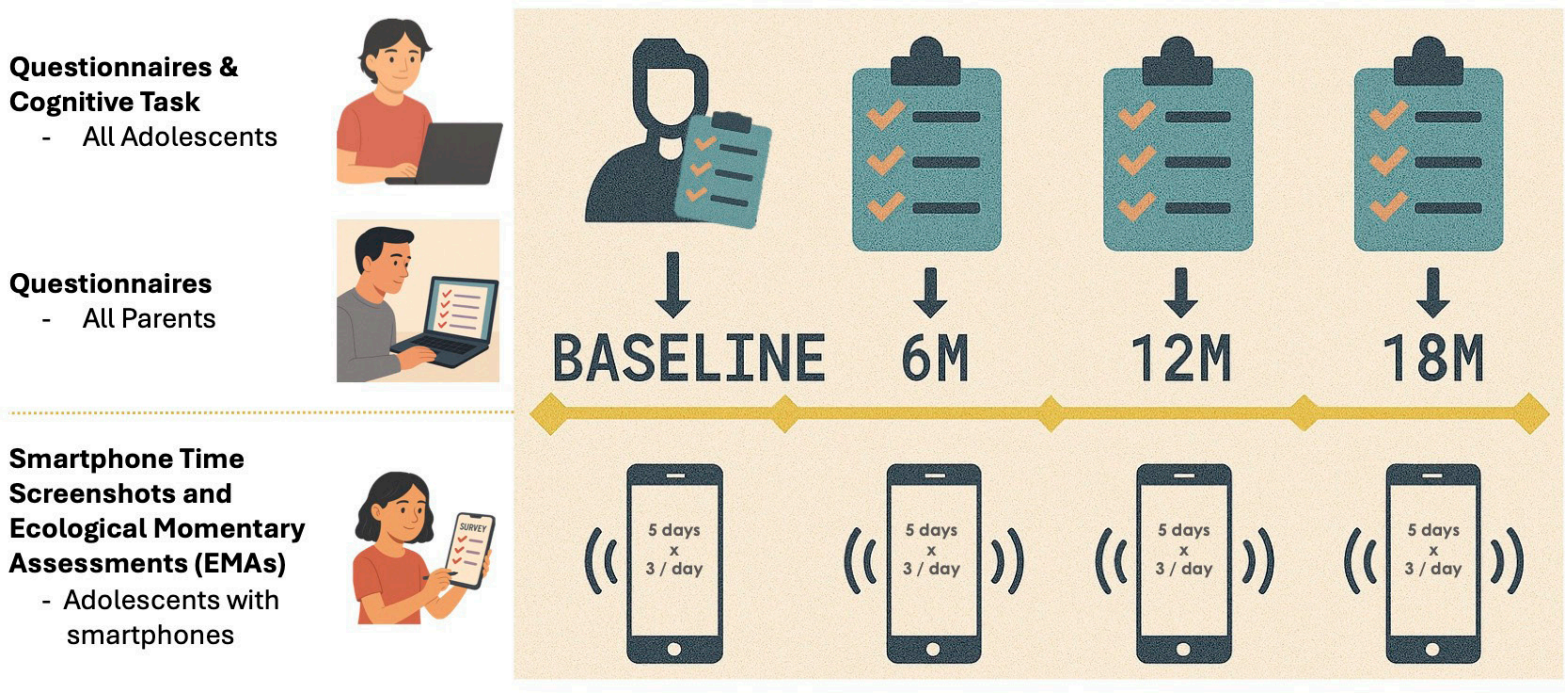
Study design: All adolescent and parent participants will complete online questionnaires at baseline and then 6, 12 and 18 months later. Adolescent participants with smartphones will also upload screenshots of their daily average screentime over the past week as part of these questionnaires and then complete a 5-day EMA period with three prompts per day outside of school hours.

This study employs an interrupted time series design (figure 1), with the introduction of the Social Media Minimum Age legislation serving as the exposure. Data will be collected from parent/carer–adolescent dyads (note; for parsimony the rest of the manuscript refers to parent–adolescent dyads but is inclusive of other caregivers) at four timepoints: baseline (collected in the 6 weeks before the implementation of the Australian Social Media Minimum Age legislation on 10 December 2025); then again at 6-month (April–May 2026), 12-month (October–November 2026) and 18-month (April–May 2027) follow-ups.

At each timepoint, adolescent participants will complete a core assessment (approximately 70 min) as well as a 5-day ecological momentary assessment (EMA). The EMA includes three brief surveys per day (approximately 2 min per survey; 30 min total). Parent participants will complete a core assessment at each timepoint (approximately 30 min).

The adolescent core assessments include a questionnaire battery of validated measures that capture anxiety, depression, well-being and disordered eating behaviours, as well as individual dispositional factors such as social sensitivity and inclination to social comparison. Attitudes and experiences relating to social media will also be captured with validated measures, as well as a set of bespoke questions capturing attitudes and behaviours relating to the Australian Social Media Minimum Age legislation (see Measurements below, online supplemental table S3 and online supplemental appendix 1 for specific measures and questions as they appear to participants). Cognitive functioning will be assessed using the affective backward digit span task (approximately 5 min), a validated measure that captures cognitive control in affective and neutral contexts.^22 23^ This will allow us to examine the potentially different impact of social media on affective versus cognitive control. In addition, adolescent participants with a smartphone will be asked to upload screen shots of overall time spent on their phone and on their most used applications over the last full week.

Parents will complete a questionnaire battery as part of each core assessment assessing parents’ digital parenting style as well as their own and their child’s social media attitudes and behaviours (online supplemental table S4 and online supplemental appendix 2).

### Participant sample size and recruitment

It was estimated that 321 adolescents would be required to achieve 80% power to detect an effect of social media exposure on mental health.^24^ Based on expected screened participant attrition of 30% across the 18-month period, we aimed to recruit 417 parent–adolescent (10–15 years old) dyads.

All adolescent and parent participants were required to be fluent in English and living in Australia. There were no other inclusion/exclusion criteria for parents. Adolescent participants were not required to have a smartphone to participate in the questionnaire component of the study. To be eligible to participate in the EMA component of the study, adolescents were additionally required to have access to an Android or iOS smartphone for the four 5-day, multi-daily survey periods following the baseline and 6-, 12- and 18-month assessments. These participants were also asked to upload screenshots of screentime as a part of the questionnaire battery. Adolescent participants could earn up to $170 (questionnaires only) or $190 (questionnaires and EMAs) and parent/carers up to $55 for completing all parts of the study.

**Figure 2:**
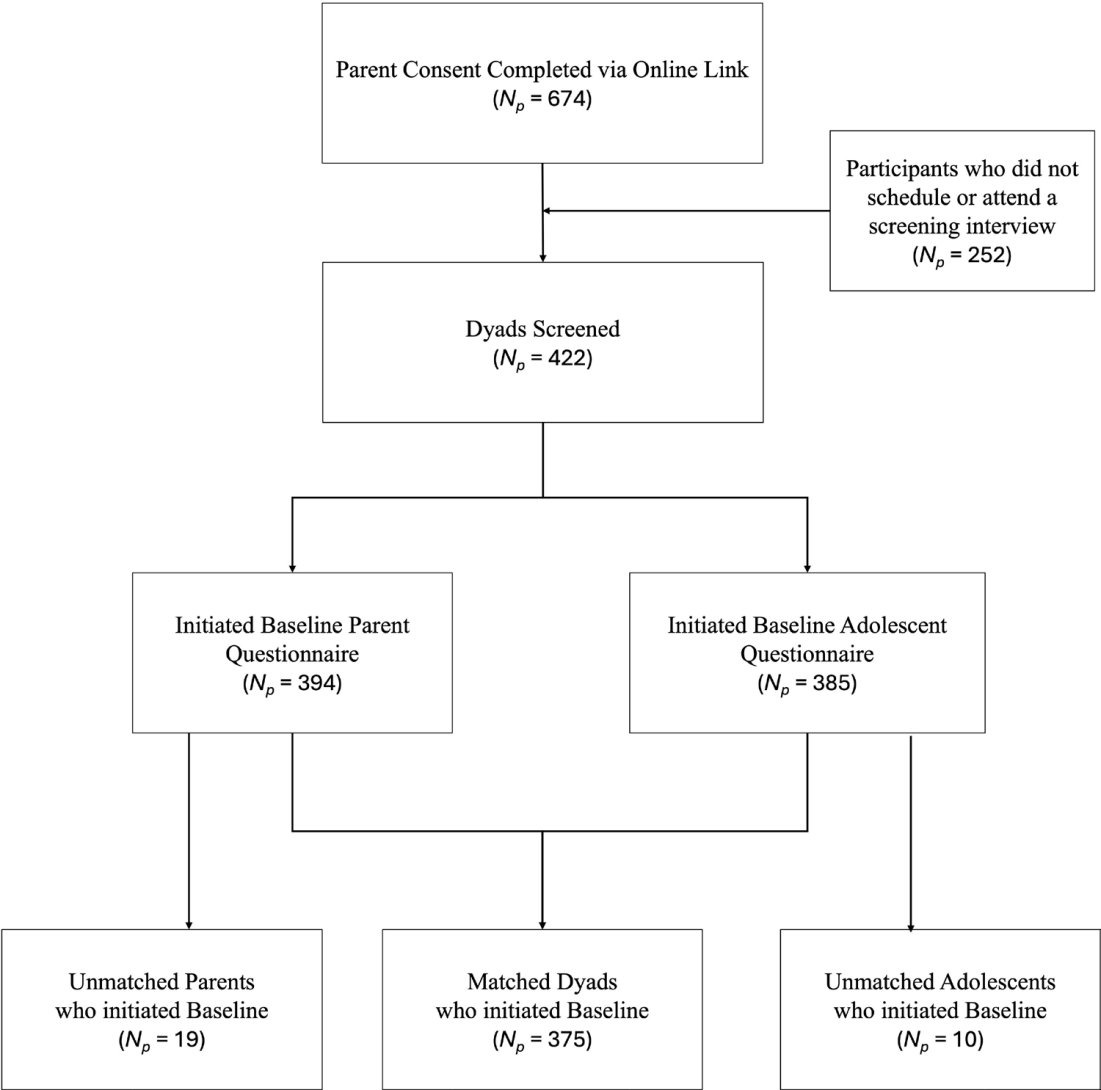
Participant inclusion flowchart*: N_p_*=number of participants.

Following approval from the UNSW Human Research Ethics Committee (iRECS9197), parent participants were recruited via social media advertising, flyers, Black Dog Institute and non-government schools’ mailing list communications and word-of-mouth before data collection from 25 October 2025 to 9 December 2025. After parents provided consent online, adolescent–parent dyads attended a Microsoft Teams interview to confirm their eligibility for the study. Questionnaires were then sent to parents and adolescents separately via email. Adolescent participants provided informed assent online prior to completing the baseline questionnaire. See figure 2 for a participant inclusion flowchart.

This resulted in a sample of 422 parent and adolescent dyads being screened. Of these, 375 matched parent–adolescent dyads commenced baseline. An additional 20 parents and 10 adolescents commenced the baseline assessment; however, they did not have a matching adolescent or parent, respectively, start the baseline survey. All dyads where at least one of the dyad (parent or adolescent) completed the baseline questionnaire will be contacted to complete the 6-, 12- and 18-month follow-up questionnaires.

The adolescent sample is broadly gender and ethnically representative of the Australian general population (online supplemental table S1), with the exception of non-binary gender identity and Aboriginal or Torres Strait Islander identity. Only 1% of adolescents identified as non-binary, and 1% identified as Aboriginal or Torres Strait Islander compared with just under 4% in the general population, according to the most recent census data.^25 26^ Though it should be noted that census data for gender identification is based on data from individuals over the age of 16 years, with 1% potentially being closer to the population average in 10–15-year-olds. The baseline sample is also less socio-demographically diverse than the general population, as the majority of parents are university educated (83%; online supplemental table S2).

All online questionnaires and surveys are administered and screenshots uploaded via Qualtrics. The digit span task will be completed on Gorilla via a link from Qualtrics. Links to all information and assessments on Qualtrics will be made available via a weblink (eg, social media ads, emails) or QR code (eg, flyers) and can be completed on participants’ own computer, tablet or phone. Participants will use their smartphones to complete EMA surveys.

### Patient and public involvement

This study has been developed in response to adolescents’, parents’ and policymakers’ expressed needs to understand how social media use relates to adolescent mental health. Public surveys indicate that, of those concerned about young people’s mental health, 44% of parents and 22% of adolescents believe social media is the thing that most impacts teen mental health,^27^ and they are looking for guidance about how to address this.^28 29^ Further, this study design is informed by ‘known evidence gaps’ identified as priorities by the US Surgeon General including, but not limited to, the impact of online versus offline social interaction on mental health, the potential pathways through which social media causes harm and the moderating role of development stage on the relationship between social media use and adolescent mental health.^2^ Importantly, all study measures, including the questionnaires, cognitive task and EMA protocol, have been validated in adolescents (10–24 years old).^21^ The research team has regularly involved youth advisors to guide us on all aspects of the research, including consultation with students across 12 schools via surveys (n=336) and focus groups (n=521) to inform the design, development and delivery of a social media literacy programme. To inform the current study, we drew on our experience and advice from young people across these activities over the last 5 years.

### Measurements

#### Questionnaire measures

##### Participant demographics

Age, gender and ethnicity were captured for parents and adolescents. Parents additionally reported on country of residence, postcode, gender, ethnicity, level of education and perceived wealth

##### Adolescent mental health

At baseline, parents reported whether adolescents have previously and/or currently received any mental health or neurodevelopmental diagnoses and/or status. At each timepoint, adolescents will complete self-report measures to capture their adverse mental health symptoms and well-being over the past 2 weeks. Adolescent generalised anxiety symptoms will be measured using the 7-item Generalised Anxiety Disorder Scale (GAD-7)^30^ with items such as ‘Not being able to stop or control worrying’ and ‘Trouble relaxing’. Adolescent depression symptoms will be assessed using the Patient Health Questionnaire-Adolescent, 8-Item Version (PHQ-8)^31–33^ with items such as ‘Little interest or pleasure in doing things’ and ‘Feeling down, depressed, irritable or hopeless’. Both the GAD-7 and PHQ-8 items are reported on a 4-point Likert scale from ‘Not at All’ to ‘Nearly Every day’. Well-being will be assessed using the Short Warwick-Edinburgh Mental Well-being Scale,^34^ with items such as “I’ve been feeling useful” and “I’ve been thinking clearly”, assessed on a 5-point Likert scale from ‘None of the Time’ to ‘All of the Time’. Eating disorder symptoms will be measured using the 8-item Eating Disorder Examination Questionnaire adapted for children^35^ with items such as “Have you tried not to eat any foods you like to control your weight and shape?” and “How often have you felt guilty after eating because of the effect on your shape and weight?”. These scales are well validated and commonly used, enabling comparison across previous and future studies.

##### Adolescent individual differences

As a key developmental vulnerability factor, social sensitivity will be measured using the Online and Offline Social Sensitivity Scale.^36^ The 18-item scale captures participants’ sensitivity to social rejection with both general and online-specific items such as “I always expect criticism” and “I delete my social media post if I don’t get the responses I wanted”. A social version of the Repetitive Negative Thinking Questionnaire will be used. The original questionnaire assesses repetitive negative thinking tendencies across generic negative situations; in this modified version, the situations are adapted to be social (eg, ‘Following unpleasant social interactions my thoughts keep repeating themselves’.). This 8-item social version has been validated in older adolescents.^11^ This questionnaire will be validated for younger adolescents in this study. Additionally, as a lot of concern around social media use focuses on the displacement of healthy habits, physical activity will be assessed using a 2-item measure assessing physical exercise time per day (using a validated single-item measure^37^) and days per week (selected from the International Physical Activity Questionnaire - Short Form^38^). Also, sleep quality will be assessed using the 8-item PROMIS Paediatric Sleep Disturbance Form.^39^ All these measures are validated in adolescent populations.

##### Adolescent and parent social media attitudes and behaviours

Adolescent internet and social media usage, including access, behaviour and parental oversight, will be assessed using questions from the LSE Toolkit^40^ as well as bespoke questions regarding age of first phone, social media and messaging use. Adolescent and parent attitudes to social media will be assessed using the Social Media Mindsets Scale,^41^ which includes both valence and agency items such as “Using social media is meaningful for me” and “I’m in control of how I use social media” respectively, measured on a 5-point Likert scale from ‘Strongly Disagree’ to ‘Strongly Agree’. An adapted version of this scale to assess parents’ attitudes to their child’s social media use. Parents’ approaches to managing and monitoring their child’s social media use will be assessed using the Digital Parenting Scale,^13^ which includes items such as “I have rules about the amount of time my child is allowed to spend on digital devices” and “I teach my child how to behave on digital devices”, measured on a 5-point Likert scale from ‘Never’ to ‘Always’.

##### Mechanisms of social media impacts

Social comparison will be assessed using the Social Comparison Scale,^42^ with 10 items such as ‘Unlikeable’ to ‘More Likeable’ and ‘Untalented’ to ‘More Talented’ reported on a 10-point semantic differential scale. Specific appearance-based comparison will also be assessed using a subscale of the State Self-Esteem Scale^43^ with items such as “I am dissatisfied with my weight” and “I feel good about myself” measured on a 5-point Likert scale from ‘Not at all’ to ‘Extremely’. Online harm exposure and responses will be assessed using five items (contact with strangers, bullying, sexual content, violence and hate messaging and actions without consent) from the EU Kids Online Research Toolkit. Examples of questions include “Have you ever had contact on social media or messaging with someone you or your family/a close friend have not met face-to-face before?” and “In the past year, have you ever seen any sexual images (not including via school or home education on puberty and sexuality)?”

##### Australian Social Media Minimum Age legislation impact

Australian adolescent and parent changes in social media use associated with the ban will be assessed using bespoke items asking about knowledge about the ban and reactions to publicity before (at baseline) and after the introduction of the ban (at 6-, 12- and 18-month follow-ups).

##### Screen shot uploads

Objectively measured total phone and online social interaction time for the prior week will be measured via smartphone screentime reports uploaded and extracted at each timepoint.

### Cognitive task

At the end of the adolescent questionnaires, participants will be redirected to Gorilla to complete the affective backwards digit span task as a measure of affective and cognitive control.^23^ Participants will be presented with a series of digits (1500 ms) superimposed on images (online supplemental figures S1A and B). After the last image in a series, they are asked to report the digits in reverse order on an on-screen number pad (online supplemental figure S1C). The task begins at a span level presenting two digits. Each span level is presented two times. The task progresses to the next span level until the participant has completed both trials at a given level incorrectly or a span of 8 digits is reached, whichever comes first. The task is presented twice in two conditions. The presentation order is counter-balanced across participants. In one condition, the images are neutral, and in the other condition, they are affective. All images are derived from the Geneva Affective Picture Database.^44^ Three indices will be derived from this task: (a) affective span and (b) neutral span (cognitive control), which are operationalised as the maximum span levels achieved in the affective and neutral conditions, respectively; and (c) affective control, which is operationalised as the proportional difference between affective and neutral span levels divided by the overall span level. Proportional scores are sensitive to performance differences that are accounted for by age-related differences in cognition.

### EMA

Participants with smartphones will also complete EMA surveys as an ecologically valid method of assessing the relative effects of adolescents’ online and offline social interactions. The surveys were designed to be completed on participants’ phones in 1–2 min (approximately 30 min total per survey period). EMA prompts will be sent outside of school hours, with the first prompt at 15:40 to capture interactions that have happened in the final hours of school, then at 17:40 and 20:00. The EMA period will run for 5 days from Wednesday to Sunday, capturing both weekday and weekend interactions. Questions largely refer to participants’ experiences in the prior 2 hours and include multiple-choice questions about participants’ most important (a) online and (b) in-person social interactions, including the type, number and closeness of people involved and how the interaction made them feel overall (affective response) and socially (affiliative response). Participants are also asked about self-reported passive versus active time spent on social media apps, as well as feelings of anxiety related to missing, or not responding fast enough to, messages and exposure to harmful content. Momentary affect, anxiety, body image, self-esteem and sense of affiliation are also measured. See online supplemental table S5 for a detailed summary of questions and sources and online supplemental appendix 3 for the questions as they appeared to participants.

### Data analysis

#### Descriptive analyses

Descriptive statistics will be used to provide an overview of the main characteristics of participant and study variables. Frequencies and percentages will be calculated for categorical variables, while means and SD will be reported for continuous variables. The reliability of scales will be calculated at each timepoint. Total scores for validated scales will be calculated according to the relevant questionnaire instructions. On the cognitive task, maximum affective span, maximum neutral span (ie, cognitive control) and affective control (ie, proportional difference score) will be reported at each timepoint. Based on prior studies,^45^ EMA compliance will be calculated based on the percentage of EMAs completed. Compliance and attrition analyses will be completed to compare completers versus non-completers on key variables.

#### Inferential analyses

Data collected throughout the study will be analysed using multiple methods, including linear regression, and mixed-effects models will be applied to questionnaire and EMA-derived data for cross-sectional and prospective analyses, respectively. These analyses will enable assessment of mental health, well-being and cognitive outcomes between individuals over time in relation to individual demographic, dispositional, developmental and experiential differences; digital parenting approaches and governmental policy adherence. The repeated measures and longitudinal design will also enable examination of changes within individuals based on changes in their online and offline social interaction behaviours over time. The core analyses will include separate models for each set of outcomes of interest: mental health, cognition and well-being. The models will include pre/post legislation and wave (baseline, 6 months, 12 months, 18 months) as fixed effects and participant ID as a random intercept effect. Changes in the proposed mechanisms of action (eg, harms exposure) will be added to these models to examine whether they account for any changes in the outcomes of interest before and after the Social Media Minimum Age legislation. Additionally, mixed-effects segmented regression will be applied to EMA-derived data to examine trends in momentary mood before and after the Social Media Minimum Age legislation. This will allow us to model mood trends across the 15 EMAs before the Social Media Minimum Age legislation and in each of the EMAs after the legislation. The inclusion of timepoints as nested variables will allow us to track the differential impact over time.

Sensitivity analyses will be conducted, including socio-economic status in the analyses, to investigate whether any effects hold when accounting for individual differences in socio-economic status indexed through self-perceived wealth and parental education. Each will be entered in separate sensitivity analyses. If sample attrition varies as a function of any specific characteristics, non-response weights will be applied.

### Ethics and dissemination

#### Ethics and safety considerations

This study has been developed by a team within the Developmental Affective Science Lab at UNSW that is experienced in conducting research with people at vulnerable stages across the lifespan. It has been approved by the UNSW Human Research Ethics Committee (iRECS 9197).

A key issue related to ethics and safety is questions pertaining to sensitive topics. To minimise any distress from questions regarding online harms (eg, cyberbullying or sexual, violent or gory content), the following measures will be implemented: (1) As part of consent all participants have been advised that there will be questions about online harms such as cyberbullying and sexual content; (2) It was emphasised to adolescent and parent participants that their participation is voluntary**,** and they may discontinue and withdraw from the study at any time, for any reason and without providing the research team with a reason; (3) Before asking questions pertaining to online harms, participants are being alerted to the content and given the opportunity to skip the section if they believe it will cause discomfort or distress; (4) Participants are given the option to ‘prefer not to say’ in response to any questions and (5) If participants feel discomfort or distress at any time during the study and would like support, details are provided for 24/7 mental health crisis support lines and/or they are able to contact the research team.

Additionally, data privacy and confidentiality are of utmost importance to protect this vulnerable population. Data will be stored on a secure UNSW OneDrive server, with access restricted to the research team. During the study, data will be stored in a re-identifiable format where any identifiers are removed and replaced with a unique code for each participant as well as a dyad code for linking parents and adolescents and a family code for linking parents and siblings. A linkage file including the personally identifiable email, phone number and random participant ID will be stored separately on UNSW OneDrive and only accessible by the chief investigator and lead researcher. Data will only be re-identified in the case of withdrawal.

### Dissemination of findings

The results of this study will be disseminated through multiple channels, including academic journals, conference presentations and researcher-related websites. Participant confidentiality will be maintained by reporting only aggregate results and not including any individually identifying information in publications. Following each assessment timepoint, core findings will also be captured in engaging formats, such as infographics and short videos. These materials will be shared with young people and their carers (eg, the following baseline descriptives were captured in an infographic; online supplemental figure S2). Participants who would like to receive the full results of the study will be able to provide their email or postal address to the research team, who will share aggregated findings only. Additionally, included participants have provided consent for de-identified information to be shared for use in future research in an open access repository that will also include all analyses and measures used in the study: https://osf.io/thky7/overview.

### Study status

Recruitment and baseline data collection were conducted between 25 October–9 December 2025 and are now complete. Limited analyses were conducted on participants’ intended actions in response to the social media ban and the relationship between these intentions and baseline mental health symptoms, with preliminary results under review for publication. No further analysis had been completed at the time of protocol submission. Following this, participants were contacted again in April 2026 (6-month follow-up), and data collection is in progress. Participants will be contacted again in October 2026 (12-month follow-up) and April 2027 (18-month follow-up) to complete the follow-up assessments.

## Supplementary material

10.1136/bmjopen-2026-120216 online supplemental file 1
