# Supplementary material for: Tracking mental health and cognitive changes following the Australian social media minimum age legislation: a 4-wave longitudinal study protocol
Source: BMJ Open. 2026 Aug 5;16(8):e120216. doi: 10.1136/bmjopen-2026-120216 (PMC13448575; doi:10.1136/bmjopen-2026-120216)

TRACKING MENTAL HEALTH AND COGNITIVE CHANGES FOLLOWING THE AUSTRALIAN SOCIAL MEDIA MINIMUM AGE LEGISLATION: A 4-WAVE LONGITUDINAL STUDY PROTOCOL

SUPPLEMENTAL MATERIAL:
Demographic Data and Questionnaires

Table of Contents

**Table S1.** *Baseline Adolescent Participant Demographics*	2
**Table S2.** *Baseline Parent/Carer Participant Demographics*	3
**Table S3.** *Overview of Adolescent Questionnaire Measures and Sources*	5
**Table S4.** *Overview of Parent Questionnaire Measures and Sources*	7
**Table S5.** *Overview of Adolescent EMA Measures and Sources*	8
**Figure S1.** *Example Backward Digit Span Images for a (a) Neutral and (b) Affective Trial Condition, and (c) On-screen number Pad Image*	10
**Appendix 1.** *Adolescent Part 1 Questionnaire Battery (Qualtrics)*	11
**Appendix 2.** *Parent Part 1 Questionnaire Battery (Qualtrics)*	50
**Appendix 3.** *Adolescent EMA Survey (Qualtrics)*	66
**Figure S2.** *Infographic Shared with Participants After Baseline*	69

Table S1. *Baseline Adolescent Participant Demographics*

Variable		Part 1 (*N*=385)
**Gender n (%)**		
	Female	177 (46%)
	Male	180 (47%)
	Non-binary / Another gender	5 (1%)
	Unknown / prefer not to say	23 (6%)
**Age in years n (%)**		
	10	28 (8%)
	11	43 (12%)
	12	73 (20%)
	13	100 (27%)
	14	70 (19%)
	15	51 (14%)
	All 10-15 years M (SD)	12.8 (1.4)
**Ethnicity n (%)**		
	White	243 (66%)
	Asian	50 (14%)
	Mixed	46 (13%)
	Aboriginal or Torres Strait Islander	4 (1%)
	Another ethnicity	15 (4%)
	Unknown / prefer not to say	27 (7%)
**Own Smartphone n (%)**		
	Yes	301 (78%)
	No	84 (22%)

Table S2. *Baseline Parent/Carer Participant Demographics*

Variable		Part 1 (*N*=395)
**Gender n (%)**		
	Female	338 (85%)
	Male	55 (14%)
	Non-binary / Another gender	1 (<1%)
	Unknown / prefer not to say	1 (<1%)
**Age (years)**		
	All parent/carers M (SD)	45.4 (7.4)
**State n (%)**		
	New South Wales	230 (58%)
	Victoria	62 (16%)
	Queensland	34 (9%)
	Western Australia	29 (7%)
	Australian Capital Territory	19 (5%)
	South Australia	14 (4%)
	Tasmania	5 (1%)
	Northern Territory	2 (<1%)
**Region n (%)**		
	Capital City	347 (88%)
	Regional	48 (12%)
**Ethnicity n (%)**		
	White	282 (71%)
	Asian	60 (15%)
	Mixed	14 (3%)
	Aboriginal or Torres Strait Islander	5 (1%)
	Another ethnicity	26 (8%)
	Prefer not to say	8 (2%)
**Perceived Wealth n (%)**		
	Not at all	5 (1%)
	Not very	37 (9%)
	Fairly	185 (47%)
	Rather	118 (30%)
	Very	34 (9%)
	Unknown / prefer not to say	16 (4%)

Variable	Part 1 (*N*=395)
**Education n (%)**	
High School	18 (5%)
Professional / vocational training	44 (11%)
University	329 (83%)
Unknown / Prefer not to say	4 (1%)
**Source of recruitment n (%)**	
Social Media Advertising	179 (45%)
Word of Mouth	79 (20%)
Black Dog Institute Mailing List	34 (9%)
Independent Schools	25 (6%)
Signed up after sibling	19 (5%)
Other / Unknown	59 (15%)

Table S3. *Overview of Adolescent Questionnaire Measures and Sources*

Category	Construct	Measure	Source
General	Demographics	Bespoke – age, gender, ethnicity	Previous UNSW research
Mental Health & Wellbeing	Anxiety	7-item Generalized Anxiety Disorder Scale (GAD-7)	[47]
	Depression	8-item Patient Health Questionnaire (PHQ-8)	[31]
	Disordered eating and body image	Eating Disorder Examination Questionnaire adapted for Children (ChEDE-Q8)	[34]
	Wellbeing	Short Warwick-Edinburgh Mental Wellbeing Scale	[48]
Smartphone and Social Media Use - Time	Extent of smartphone screen time and social media and messaging app usage	Objectively measured phone and social media time for the past week will be measured via screen time reports (including the screentime overview statistic and time spent on each app) uploaded and extracted at each timepoint.<br>This procedure was successfully piloted in a 2024 study by the same research team	[45]
Social Media - Use, Behaviours and Attitudes	Internet and social media access and behaviour	From the LSE EU Kids Online Research Toolkit (see lse.ac.uk/media-and-communications/research/research-projects/eu-kids-online/toolkit)	[40]
	Age of first phone and social media use	Bespoke questions	
	Attitudes to social media use	Social Media Mindsets Scale - validated in adults. This study will assess the validity of this measure in adolescents.	[41]
	Compliance with Social Media Minimum Age legislation and changes in social media and messaging app usage	Mix of questions from the EU Kids Online Research Toolkit (see lse.ac.uk/media-and-communications/research/research-projects/eu-kids-online/toolkit) and questions novel to this study. In the latter case, the sample characteristics can be compared to data from large-scale adolescent cohorts that have been tracking social media use in this age group over recent years [49,50]	[40]
Individual differences – *Dispositional*	Social Sensitivity	Online and Offline Social Sensitivity Scale	[51]
	Social Repetitive Negative Thinking	Scale developed by the same research team in 2024 and validated in 190 older adolescents with good validity and reliability. Includes 8-items rated on a 5-	[35]

		point Likert scale from "Not true at All" to "Very true" to assess participants tendency to engage in repetitive negative thinking about social interactions, which may potentiate the effects of interacting online. This will be validated for younger adolescents in this study.	
	Social and appearance-based comparison	Social Comparison Scale	[52]
		Appearance subscale of the State Self-Esteem Scale	[53]
Individual differences: *Experiential*	Online harm exposure	6-items assessing online harm exposure (upsetting encounters, contact with strangers, bullying, sexual content, violence and hate messaging, and actions without consent) from the EU Kids Online Research Toolkit (see lse.ac.uk/media-and-communications/research/research-projects/eu-kids-online/toolkit)	[40]
Individual differences: *Physical*	Physical Activity	2-item measure assessing minutes of activity per day and number of days of activity per week	[37,38]
	Sleep	8-Item PROMIS Pediatric Sleep Disturbance Form	[39]
Cognition	Working Memory / Processing Speed /Cognitive and Affective control	Affective backward digit span task, requiring participants to enter sequentially presented digits in reverse order. Digits are presented superimposed over neutral (e.g., chair) or negative (e.g., snakes, spiders) stimuli. The task ends when participants complete both trials at a given span level incorrectly. Cognitive and affective control will be operationalised as the number of digits remembered in each condition	[54,55]

Table S4. *Overview of Parent Questionnaire Measures and Sources*

Category	Construct	Measure	Source
General / Child Mental Health History	Demographics	Bespoke – age, postcode, gender, ethnicity, level of education, perceived wealth	Previous UNSW research
	Child mental and neurological health and development	Existence of past and current diagnoses of mental health, neurological or neurodevelopmental conditions	
Child's Social Media Use - Behaviours and Attitudes	Internet and social media permissions and behaviour	Adapted from the LSE EU Kids Online Research Toolkit (see lse.ac.uk/media-and-communications/research/research-projects/eu-kids-online/toolkit)	[40]
	Age of first phone and social media use	Bespoke question	
	Attitudes to child's social media use	Adapted Social Media Mindsets Scale - This study will assess the validity of this as a measure of parents' attitudes to child behaviour	[41]
	Parent engagement with and approach to Social Media Minimum Age legislation	Mix of questions from the EU Kids Online Research Toolkit (see lse.ac.uk/media-and-communications/research/research-projects/eu-kids-online/toolkit) and questions novel to this study.	[40]
	Digital Parenting	Digital Parenting Scale	[12]
	Compliance with Social Media Minimum Age legislation and changes in social media and messaging app usage	Bespoke items measuring their child's compliance with the age-restrictions at each time point, and changes in their child's social media use compared to prior to the publicity about the ban (at baseline) / introduction of the ban (post 2025).	
Parent's Own Social Media Use - Behaviours and Attitudes	Internet and social media usage and behaviour	As parental modelling is known to influence child behaviour, parents will be asked similar questions to those asked of their adolescents about their own use of social media.	[40]
	Attitudes to own social media use	Social Media Mindsets Scale	[41]
	Approach to Social Media Minimum Age legislation and changes in social media and messaging app usage	Bespoke items measuring their own social media use compared to prior to the publicity about the ban (at baseline) / introduction of the ban (post 2025).	

Table S5. *Overview of Adolescent EMA Measures*

Category	Construct	Measure	Source
Mental Health & Wellbeing	Momentary Feelings	Feeling “right now” rated from 1-100 for<br>• Affect: Very Unhappy-Very Happy<br>• Anxiety: Anxious-Relaxed<br>• Body Image: Uncomfortable - Comfortable	[56–58]
	Momentary Self-perception	Feeling “relative to others right now” rated from 1-100<br>• Likeability: Unlikeable – Likeable<br>• Affiliation: Left Out - Included	[57]
All the remaining questions related to experiences in the prior 2 hours:			
Social Media & Messaging –Behaviours & Responses	Momentary Online Social Interaction – Nature and responses	Minutes passively reading or viewing online posts or messages with known and unknown people	
		Whether any online interaction<br>> For their single most important online social interaction:<br>• Platform<br>• Known v unknown people<br>• Number of people	[59,60]
		How the interaction made them feel rated from 1-100 for<br>• Affect: Very Unhappy-Very Happy<br>• Affiliation: Left Out - Included	[57,58]
	Online Social Interaction-related anxiety	Novel questions regarding frequency on a 5-point Likert scale from “Not at All” to “Constantly” for how worried they have felt re:<br>• Missing messages or posts<br>• Responding fast enough to messages or posts<br>• Other people not responding to messages or updates<br>• Other people misunderstanding an update	
In-person Social Interactions – Behaviours & Responses	Momentary In-Person Social Interaction – Nature	Whether any in-person interaction > For their single most important in-person social interaction:<br>• Known v unknown people<br>• Number of people	[59,60]
	Momentary In-Person Social Interaction – Responses	How the interaction made them feel rated from 1-100 for<br>• Affect: Very Unhappy-Very Happy<br>• Affiliation: Left Out - Included	[57,58]
Individual differences – *Dispositional*	Momentary social and appearance-	Whether they have compared their appearance to another<br>• Context (online & offline)	[56,57]

	based comparison	• Result of comparison on a 5-point Likert scale from “Much better” to “Much worse”	
Individual differences: *Experiential*	Momentary online harm exposure	• Whether they have been “treated in a nasty or hurtful way” online<br>○ Platform	Adapted from [40]
		• Whether they have seen images they “did not want to see”<br>○ Platform	

Figure S1. *Example Backward Digit Span Images for a (a) Neutral and (b) Affective Trial Condition, and (c) On-screen number Pad Image*

(a)

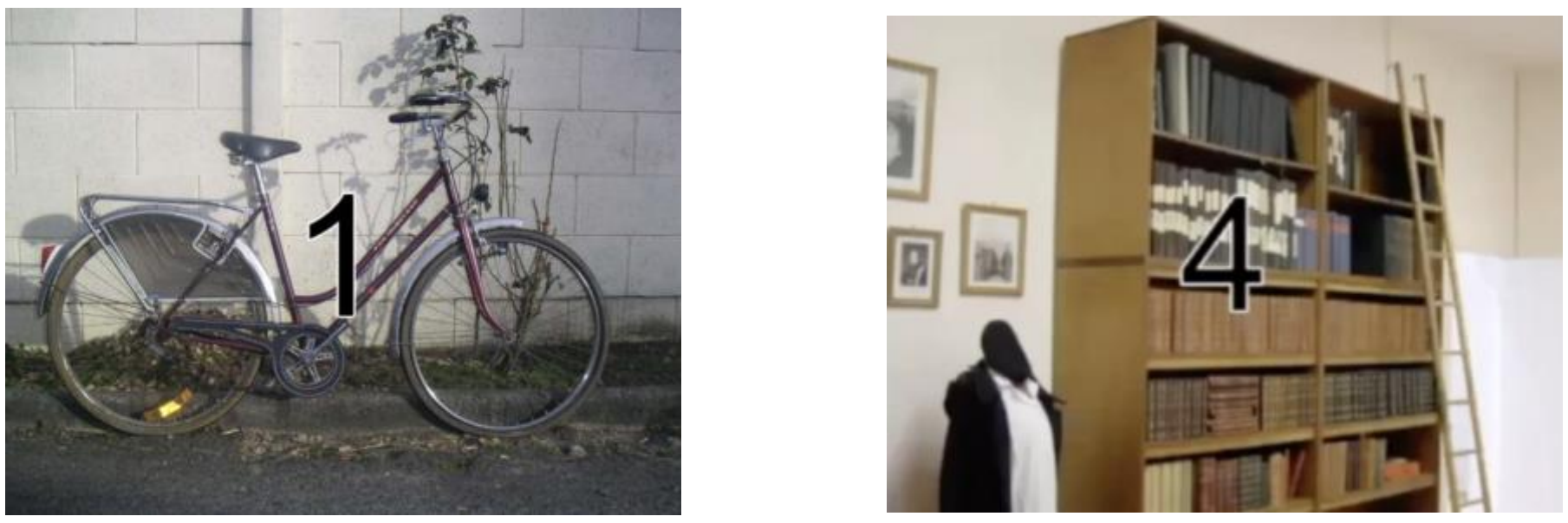

(b)

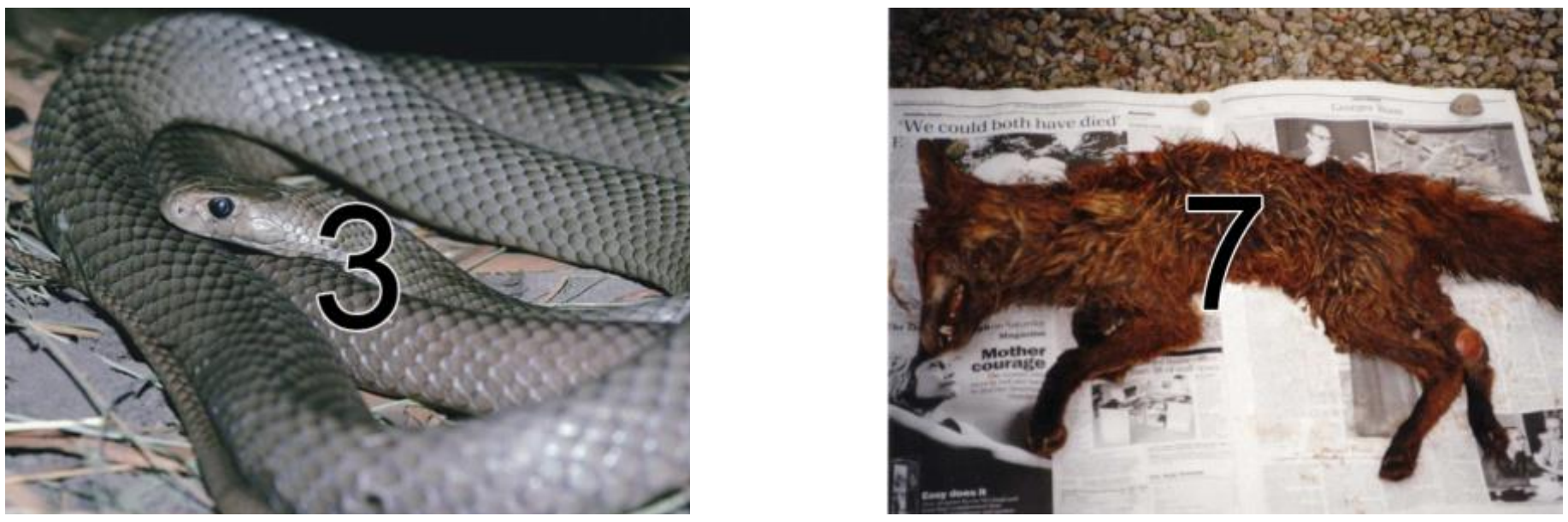

(c)

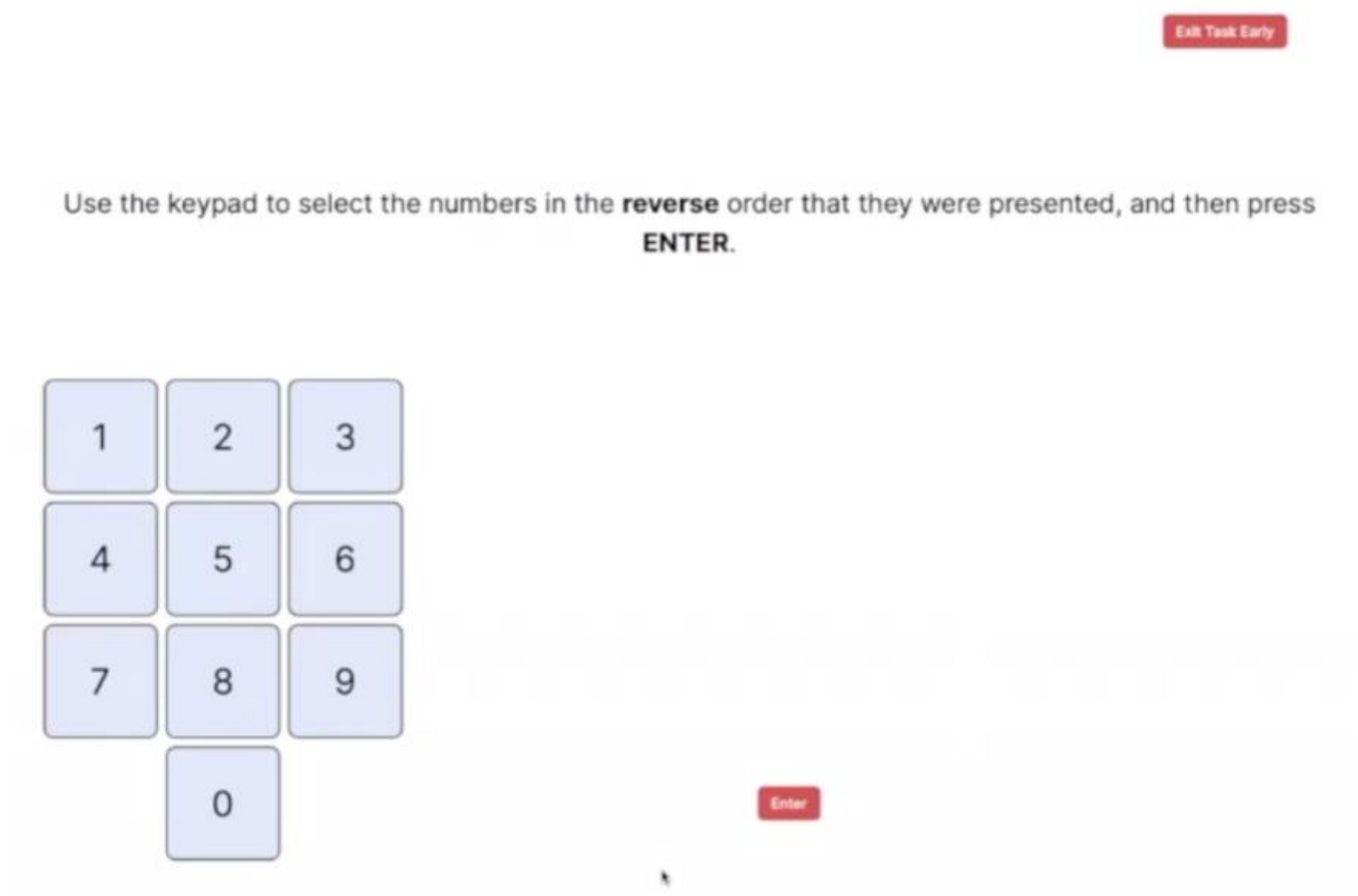

Appendix 1. *Adolescent Part 1 Questionnaire Battery*

1.1.1 - OSIYAW - Adolescent Questionnaires

ExpertReview score Fair

Landing & Consent - A_Aus - Phone

LP - A_Phone

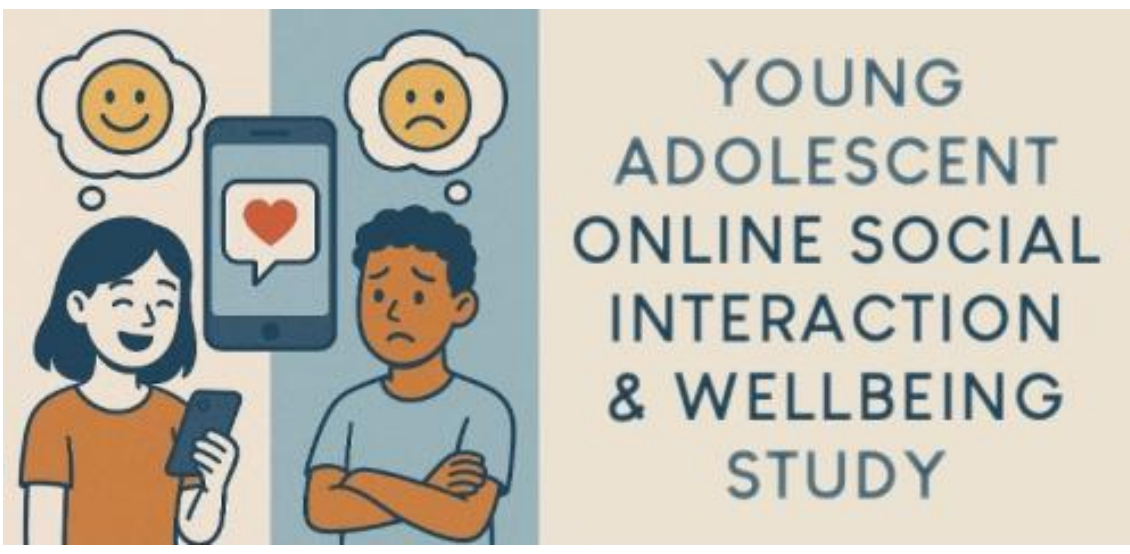

You are invited to participate in our study!

Is using social media and messaging a good or bad thing for young people?
For whom? Why?

We're looking for young people aged 10–15 years old to tell us about your experiences connecting online and in-person.

The study involves four parts - now, and in 6-, 12- and 18-months time. At each timepoint we will ask you to complete:

- Questionnaires
- A short cognitive task
- Screenshots uploads of your phone screen time (overall and most used apps) for the last full week.
- A short survey 3-times-a-day for 5 days about how you are feeling and your recent online and offline social interactions

Completing all parts of the study will earn you a **A$170** Amazon voucher, with potential additional **bonuses of up to A$20** for timely completion

Interested?

Page Break

PISCF - A_Aus - Ph

To make sure you are happy with participating in this research please take the time to read this participant information statement and, if you'd like to participate indicate with "I agree" below:

This study has been approved by UNSW Human Research Ethics Committee (iRECS 9197) who check that the research we are conducting with you is useful and make sure your rights and privacy are protected.

1. **What is this research study about?**
   The research study aims to investigate the relationship between online social interactions and mental health, and what factors predict whether someone will find online social interactions beneficial or not. There has been lots of public interest in whether the rise in social media use is related to an increase in youth mental health problems, and we hope to use information we gain from this research study to contribute to our understanding of the pros and cons of interacting online.

2. **Who is conducting this research?**
   The study is being carried out by the following researchers: Scientia Associate Professor, Dr Susanne Schweizer (Chief Investigator), Anna Bilston (Lead Researcher / PhD Candidate), Associate Professor Dr Aliza Werner-Siedler (Black Dog Institute), Dr Lucy Zhang (Post Doctoral Researcher), Dr Lyndsay Brown, (Black Dog Institute), Dr Sarah Stevens (Black Dog Institute), Yasmin Hasan (Research Assistant), Emily Morgan (Research Assistant), Weike Wang (PhD Student), Xu Qi (Third Year Student) and Kirsten Hertog (Third Year Student) at the UNSW School of Psychology.
   Research Funder: This research is being funded by the Australian Research Council.

3. **Inclusion/Exclusion Criteria**
   Before you decide to participate in this research study, we need to ensure that it is ok for you to take part. The research study is looking recruit people who meet the following criteria:
   - The young person needs to be 10-15 years old
   - The young person must have access to an Android or IOS smartphone that will be available for the three 5-day, multi-daily survey periods at baseline, and 6-, 12- and 18-months later
   - The young person and parent/carer need to have very good command of written and spoken English
   - Live in Australia, the UK, or NZ

4. **Do I have to take part in this research study?**
   Participation in this research study is **voluntary**. If you do not want to take part, you do not have to. If you agree to take part and later change your mind, you are free to withdraw yourself from the study at any stage.
   If you decide you want to take part in the research study, you will be asked to:
   - Read this study information carefully (ask questions if necessary).
   - Provide consent at the bottom of this page if you decide to participate in the study.
   - Keep a copy of this form.

5. **What does participation in this research require, and are there any risks involved?**
If you agree to participate, we will first ask you to complete a brief 2-5-minute video phone call involving you, your parent / carer and a member of the research team, to confirm your eligibility. The call will be recorded as evidence of eligibility. After that call, we will invite you to complete the five-part study.

You will then be asked to complete the following:
1. Complete an online questionnaire asking you to answer questions about how you have felt in the last week generally, how you feel in social situations online and offline and the type of content you encounter. You will also be asked to complete a short cognitive task and upload screenshots of your total phone screentime and time on each application. This should take approximately 75 minutes to complete.
2. You will then be contacted via SMS three (3) times per day, for five (5) days (outside school hours) to fill in brief surveys (1-2 minutes) about how you are feeling, any online and offline social interactions you have had, and what type (if any) of online content you have encountered in the prior two hours.
3. You will be invited to complete the questionnaires and SMS questions again after 6-, 12-, and 18-months. At each time point, participants will also complete a short cognitive task (~5 min) and be asked to upload screenshots of their total phone screentime and time on each application.

The research team will contact you via email and/or SMS to remind you when to complete the next round of questionnaires. You will receive an initial reminder, and one follow up reminder. You can opt out of reminders and withdraw from the study at any time.

Costs and Remuneration: There are no costs associated with participating in this research project. Upon full and faithful completion of the study you will be reimbursed for each section completed in Amazon gift vouchers:
- For each of the four assessments (~75min each) you will receive A$22.50 (total A$90). You will also receive bonus payments if you complete all your follow-up assessments (Time 2-4) in full. If you complete the assessment at Time 2 (~6 months after Time 1) you will receive an extra A$5; for Time 3 (~12 months after Time 1) you will receive an extra A$10 and for Time 4 (~18 months after Time 1) an extra A$15. That means if you complete all four assessments in full you will receive a total of A$120.
- For each of the four multiple daily survey periods (3 SMS/day for 5 days) you will receive A$12.50 (each). Total of A$50.
- That is, for full and faithful study completion you will receive A$170.
- Faithful completion means that you cannot complete more than two of the attention checks incorrectly at any timepoint. Attention checks are questions that are inserted into the study to make sure you are paying attention to the study instructions.
- Additionally, you will be eligible for a further bonus payment of A$5 for each 5-day SMS period if you respond to each SMS prompt within one (1) hour. That is, you can earn an additional total of A$20.

Some questions may be upsetting. This information will be clearly stated before sensitive questions are presented. If you do not wish to answer a question or complete a task, you may skip it and go to the next question or stop immediately. If you experience discomfort or feelings of distress while participating in the research and you require support, you can stop participating at any time. You can also contact the research team with any questions or request to be contacted by the research team's clinical psychologist. Alternatively, a list of support services and their contact details are provided below.

6. **What are the possible benefits to participation?**
We hope to use information we get from this research study to contribute to our global understanding of the pros and cons of interacting online to help young people, parents and governments make the right decisions about how to manage, monitor and regulate young people's access to social media

7. **What will happen to information about me?**
By clicking the consent button, you consent to the research team collecting and using information about you for the research study. Information and recordings collected from you will be stored on a UNSW password protected One Drive only accessible to the approved research investigators. During the study, data will be stored in a re-identifiable format where any identifiers such as your name and email will be replaced with a unique code. The research team will take all reasonable steps to protect your privacy. After the analysis is complete, the research team will store the data collected from you for this research project for a minimum of 7 years after the publication of research results.

You will be asked to provide your consent for the research team to share or use information collected from you in future research. Your information will only be shared in a format that will not identify you. This fully anonymised data (that means you cannot be identified through any means) will be made available on an open access repository for other researchers who consent to adhering to ethical research practices.
The information you provides is personal information for the purposes of the Privacy and Personal Information Protection Act 1998 (NSW). You have the right of access to personal information held about you by the University, the right to request correction and amendment of it, and the right to make a complaint about a breach of the Information Protection Principles as contained in the PPIP Act. Further information on how the University protects personal information is available in the **UNSW Privacy Management Plan**.

8. **How and when will I find out what the results of the research study are?**
The research team intend to publish and report the results of the research. All Information will be published in a way that will not identify you.

If you would like to receive a copy of the results you can let the research team know by inserting your email or mailing address in the consent form. We will only use these details to send you the results of the research.

9. **What if I want to withdraw from the research study?**
If you do consent to participate, you may withdraw at any time. You can do so by contacting the research team and telling them that you no longer want to participate. Your decision not to participate or to withdraw from the study will not affect your relationship with UNSW, Sydney or any organisation helping with recruitment for this research e.g. your school or sports club. If you decide to leave the research study, the researchers will not collect additional information from you. You can request that any identifiable information about you be withdrawn from the research project.

10. **What should I do if I have further questions about my involvement in the research study?**
The person you may need to contact will depend on the nature of your query. If you require further information regarding this study or if you have any problems which may be related to your involvement in the study, you can contact the following member/s of the research team. We will aim to answer within two business days.

Research Team Contact Details

Lead Researcher

**Name**	Anna Bilston
**Position**	PhD Candidate
**Email**	a.bilston@unsw.edu.au

Chief Investigator

**Name**	Susanne Schweizer

**Position**	Scientia Associate Professor, School of Psychology, UNSW
**Telephone**	+61 2 9348 1896
**Email**	DASLab@unsw.edu.au<br>s.schweizer@unsw.edu.au

Support Services Contact Details

If at any stage during the study, you feel distressed or require additional support from someone not involved in the research please contact one of the following free, 24/7 support services. Many have text or online chat services if you are not comfortable or not able to call, as provided:

**Name/Organisation**	Lifeline
**Position**	Crisis counsellor
**Telephone**	13 11 14
**Text Support**	0477 13 11 14
**Online Chat**	https://www.lifeline.org.au/crisis-chat/

**Name/Organisation**	13YARN (Aboriginal
**Position**	Aboriginal or Torres Strait Islander Crisis Supporter
**Telephone**	13 92 76

**Name/Organisation**	1800RESPECT
**Position**	Sexual assault, family and domestic violence counsellor
**Telephone**	1800 737 732
**Text Support**	0458 737 732
**Online Chat link:**	https://www.1800respect.org.au/online-chat-1800respect

**Name/Organisation**	Beyond Blue
**Position**	Counsellor
**Telephone**	1300 22 4636
**Online Chat link:**	https://www.beyondblue.org.au/get-support/talk-to-a-counsellor/chat

**Name/Organisation**	Kids Helpline
**Position**	Counsellor
**Telephone**	1800 55 1800
**Online Chat link**	https://kidshelpline.com.au/get-help/webchat-counselling

11. What if I have a complaint or any concerns about the research study?

If you have a complaint regarding any aspect of the study or the way it is being conducted, please contact the UNSW Human Ethics Coordinator:

Complaints Contact

**Position**	UNSW Human Research Ethics Coordinator
**Telephone**	+ 61 2 9385 6222
**Email**	humanethics@unsw.edu.au
**HC Reference Number**	iRECS 9197

Consent Form

Declaration by the participant

By checking "I agree" below:

- I understand I am being asked to provide consent to participate in this research study;
- I have read the Participant Information Sheet, or it has been provided to me in a language that I understand;
- I understand the purposes, study tasks and risks of the research described in the study;
- I understand that the research team will video record the short screening interview; I agree to be recorded for this purpose.
- I provide my consent for the information collected about me to be used for the purpose of this research study.
- I consent for fully anonymised data (that means I cannot be identified by any means) to be made accessible to other researchers on an open access repository.
- I have had an opportunity to ask questions, and I am satisfied with the answers I have received.
- I freely agree to participate in this research study as described and understand that I am free to withdraw at any time during the study and withdrawal will not affect my relatinship with any of the named organisations and/or research team members
- I understand that the results of the research will be made available on the DAS Lab website

○ I DO NOT Agree

○ **I Agree**

Q74

To make sure you are happy with participating in this research please take the time to read this participant information statement and, if you'd like to participate indicate with "I agree" below:

This study has been approved by UNSW Human Research Ethics Committee (iRECS 9197) who check that the research we are conducting with you is useful and make sure your rights and privacy are protected.

1. **What is this research study about?**
   The research study aims to investigate the relationship between online social interactions and mental health, and what factors predict whether someone will find online social interactions beneficial or not. There has been lots of public interest in whether the rise in social media use is related to an increase in youth mental health problems, and we hope to use information we gain from this research study to contribute to our understanding of the pros and cons of interacting online.

2. **Who is conducting this research?**
   The study is being carried out by the following researchers: Scientia Associate Professor, Dr

Susanne Schweizer (Chief Investigator), Anna Bilston (Lead Researcher / PhD Candidate), Associate Professor Dr Aliza Werner-Siedler (Black Dog Institute), Dr Lucy Zhang (Post Doctoral Researcher), Dr Lyndsay Brown, (Black Dog Institute), Dr Sarah Stevens (Black Dog Institute), Yasmin Hasan (Research Assistant), Emily Morgan (Research Assistant), Weike Wang (PhD Student), Xu Qi (Third Year Student) and Kirsten Hertog (Third Year Student) at the UNSW School of Psychology.

Research Funder: This research is being funded by the Australian Research Council.

3. **Inclusion/Exclusion Criteria**

Before you decide to participate in this research study, we need to ensure that it is ok for you to take part. The research study is looking recruit people who meet the following criteria:

- The young person needs to be 10-15 years old
- The young person must have access to an Android or IOS smartphone that will be available for the three 5-day, multi-daily survey periods at baseline, and 6-, 12- and 18-months later
- The young person and parent/carer need to have very good command of written and spoken English
- Live in Australia, the UK, or NZ

4. **Do I have to take part in this research study?**

Participation in this research study is **voluntary**. If you do not want to take part, you do not have to. If you agree to take part and later change your mind, you are free to withdraw yourself from the study at any stage.

If you decide you want to take part in the research study, you will be asked to:

- Read this study information carefully (ask questions if necessary).
- Provide consent at the bottom of this page if you decide to participate in the study.
- Keep a copy of this form.

5. **What does participation in this research require, and are there any risks involved?**

If you agree to participate, we will first ask you to complete a brief 2-5-minute video phone call involving you, your parent / carer and a member of the research team, to confirm your eligibility. The call will be recorded as evidence of eligibility. After that call, we will invite you to complete the five-part study.

You will then be asked to complete the following:

1. Complete an online questionnaire asking you to answer questions about how you have felt in the last week generally, how you feel in social situations online and offline and the type of content you encounter. You will also be asked to complete a short cognitive task and upload screenshots of your total phone screentime and time on each application. This should take approximately 75 minutes to complete.
2. You will then be contacted via SMS three (3) times per day, for five (5) days (outside school hours) to fill in brief surveys (1-2 minutes) about how you are feeling, any online and offline social interactions you have had, and what type (if any) of online content you have encountered in the prior two hours.
3. You will be invited to complete the questionnaires and SMS questions again after 6-, 12-, and 18-months. At each time point, participants will also complete a short cognitive task (~5 min) and be asked to upload screenshots of their total phone screentime and time on each application.

The research team will contact you via email and/or SMS to remind you when to complete the next round of questionnaires. You will receive an initial reminder, and one follow up reminder. You can opt out of reminders and withdraw from the study at any time.

Costs and Remuneration: There are <u>no costs</u> associated with participating in this research project. Upon full and faithful completion of the study you will be reimbursed for each section completed in Amazon gift vouchers:

- For each of the four assessments (~75min each) you will receive A$22.50 (total A$90). You will also receive bonus payments if you complete all your follow-up assessments (Time 2-4) in full. If you complete the assessment at Time 2 (~6 months after Time 1) you will receive an extra A$5; for Time 3 (~12 months after Time 1) you will receive an extra A$10 and for Time 4 (~18 months after Time 1) an extra A$15. That means if you complete all four assessments in full you will receive a total of A$120.
- For each of the four multiple daily survey periods (3 SMS/day for 5 days) you will receive A$12.50 (each). Total of A$50.
- That is, for full and faithful study completion you will receive A$170.
- Faithful completion means that you cannot complete more than two of the attention checks incorrectly at any timepoint. Attention checks are questions that are inserted into the study to make sure you are paying attention to the study instructions.
- Additionally, you will be eligible for a further bonus payment of A$5 for each 5-day SMS period if you respond to each SMS prompt within one (1) hour. That is, you can earn an additional total of A$20.

Some questions may be upsetting. This information will be clearly stated before sensitive questions are presented. If you do not wish to answer a question or complete a task, you may skip it and go to the next question or stop immediately. If you experience discomfort or feelings of distress while participating in the research and you require support, you can stop participating at any time. You can also contact the research team with any questions or request to be contacted by the research team's clinical psychologist. Alternatively, a list of support services and their contact details are provided below.

6. **What are the possible benefits to participation?**

We hope to use information we get from this research study to contribute to our global understanding of the pros and cons of interacting online to help young people, parents and governments make the right decisions about how to manage, monitor and regulate young people's access to social media

7. **What will happen to information about me?**

By clicking the consent button, you consent to the research team collecting and using information about you for the research study. Information and recordings collected from you will be stored on a UNSW password protected One Drive only accessible to the approved research investigators. During the study, data will be stored in a re-identifiable format where any identifiers such as your name and email will be replaced with a unique code. The research team will take all reasonable steps to protect your privacy. After the analysis is complete, the research team will store the data collected from you for this research project for a minimum of 7 years after the publication of research results.

You will be asked to provide your consent for the research team to share or use information collected from you in future research. Your information will only be shared in a format that will not identify you. This fully anonymised data (that means you cannot be identified through any means) will be made available on an open access repository for other researchers who consent to adhering to ethical research practices.

The information you provides is personal information for the purposes of the Privacy and Personal Information Protection Act 1998 (NSW). You have the right of access to personal information held about you by the University, the right to request correction and amendment of it, and the right to make a complaint about a breach of the Information Protection Principles as contained in the PPIP Act. Further information on how the University protects personal information is available in the **<u>UNSW Privacy Management Plan</u>**.

8. **How and when will I find out what the results of the research study are?**

The research team intend to publish and report the results of the research. All Information will be published in a way that will not identify you.

published in a way that will not identify you.

If you would like to receive a copy of the results you can let the research team know by inserting your email or mailing address in the consent form. We will only use these details to send you the results of the research.

9. What if I want to withdraw from the research study?
If you do consent to participate, you may withdraw at any time. You can do so by contacting the research team and telling them that you no longer want to participate. Your decision not to participate or to withdraw from the study will not affect your relationship with UNSW, Sydney or any organisation helping with recruitment for this research e.g. your school or sports club. If you decide to leave the research study, the researchers will not collect additional information from you. You can request that any identifiable information about you be withdrawn from the research project.

10. What should I do if I have further questions about my involvement in the research study?
The person you may need to contact will depend on the nature of your query. If you require further information regarding this study or if you have any problems which may be related to your involvement in the study, you can contact the following member/s of the research team. We will aim to answer within two business days.

Research Team Contact Details

Lead Researcher

**Name**	Anna Bilston
**Position**	PhD Candidate
**Email**	a.bilston@unsw.edu.au

Chief Investigator

**Name**	Susanne Schweizer
**Position**	Scientia Associate Professor, School of Psychology, UNSW
**Telephone**	+61 2 9348 1896
**Email**	DASLab@unsw.edu.au<br>s.schweizer@unsw.edu.au

Support Services Contact Details
If at any stage during the study, you feel distressed or require additional support from someone not involved in the research please contact one of the following free, 24/7 support services. Many have text or online chat services if you are not comfortable or not able to call, as provided:

**Name/Organisation**	Lifeline
**Position**	Crisis counsellor
**Telephone**	13 11 14
**Text Support**	0477 13 11 14
**Online Chat**	https://www.lifeline.org.au/crisis-chat/

**Name/Organisation**	13YARN (Aboriginal
**Position**	Aboriginal or Torres Strait Islander Crisis Supporter
**Telephone**	13 92 76

**Name/Organisation**	1800RESPECT
**Position**	Sexual assault, family and domestic violence counsellor
**Telephone**	1800 737 732
**Text Support**	0458 737 732
**Online Chat link:**	https://www.1800respect.org.au/online-chat-1800respect

**Name/Organisation**	Beyond Blue
**Position**	Counsellor
**Telephone**	1300 22 4636
**Online Chat link:**	https://www.beyondblue.org.au/get-support/talk-to-a-counsellor/chat

**Name/Organisation**	Kids Helpline
**Position**	Counsellor
**Telephone**	1800 55 1800
**Online Chat link**	https://kidshelpline.com.au/get-help/webchat-counselling

11. What if I have a complaint or any concerns about the research study?
If you have a complaint regarding any aspect of the study or the way it is being conducted, please contact the UNSW Human Ethics Coordinator:

Complaints Contact

**Position**	UNSW Human Research Ethics Coordinator
**Telephone**	+ 61 2 9385 6222
**Email**	humanethics@unsw.edu.au
**HC Reference Number**	iRECS 9197

Consent Form

Declaration by the participant

By checking "I agree" below:

- I understand I am being asked to provide consent to participate in this research study;
- I have read the Participant Information Sheet, or it has been provided to me in a language that I understand;
- I understand the purposes, study tasks and risks of the research described in the study;
- I understand that the research team will video record the short screening interview; I agree to be recorded for this purpose.
- I provide my consent for the information collected about me to be used for the purpose of this research study.
- I consent for fully anonymised data (that means I cannot be identified by any means) to be made accessible to other researchers on an open access repository.
- I have had an opportunity to ask questions, and I am satisfied with the answers I have received.
- I freely agree to participate in this research study as described and understand that I am free to withdraw at any time during the study and withdrawal will not affect my relatinship with any of the named organisations and/or research team members
- I understand that the results of the research will be made available on the DAS Lab website

○ I DO NOT Agree

○ **I Agree**

Q73

To make sure you are happy with participating in this research <u>please take the time to read this participant information statement</u> and, if you'd like to participate indicate with "I agree" below:

This study has been approved by UNSW Human Research Ethics Committee (iRECS 9197) who check that the research we are conducting with you is useful and make sure your rights and privacy are protected.

1. **What is this research study about?**
   The research study aims to investigate the relationship between online social interactions and mental health, and what factors predict whether someone will find online social interactions beneficial or not. There has been lots of public interest in whether the rise in social media use is related to an increase in youth mental health problems, and we hope to use information we gain from this research study to contribute to our understanding of the pros and cons of interacting online.

2. **Who is conducting this research?**
   The study is being carried out by the following researchers: Scientia Associate Professor, Dr Susanne Schweizer (Chief Investigator), Anna Bilston (Lead Researcher / PhD Candidate), Associate Professor Dr Aliza Werner-Siedler (Black Dog Institute), Dr Lucy Zhang (Post Doctoral Researcher), Dr Lyndsay Brown, (Black Dog Institute), Dr Sarah Stevens (Black Dog Institute), Yasmin Hasan (Research Assistant), Emily Morgan (Research Assistant), Weike Wang (PhD Student), Xu Qi (Third Year Student) and Kirsten Hertog (Third Year Student) at the UNSW School of Psychology.
   Research Funder: This research is being funded by the Australian Research Council.

3. **Inclusion/Exclusion Criteria**
   Before you decide to participate in this research study, we need to ensure that it is ok for you to take part. The research study is looking recruit people who meet the following criteria:
   - The young person needs to be 10-15 years old
   - The young person must have access to an Android or IOS smartphone that will be available for the three 5-day, multi-daily survey periods at baseline, and 6-, 12- and 18-months later
   - The young person and parent/carer need to have very good command of written and spoken English
   - Live in Australia, the UK, or NZ

4. **Do I have to take part in this research study?**
   Participation in this research study is **voluntary**. If you do not want to take part, you do not have to. If you agree to take part and later change your mind, you are free to withdraw yourself from the study at any stage.
   If you decide you want to take part in the research study, you will be asked to:
   - Read this study information carefully (ask questions if necessary).
   - Provide consent at the bottom of this page if you decide to participate in the study.
   - Keep a copy of this form.

5. **What does participation in this research require, and are there any risks involved?**
   If you agree to participate, we will first ask you to complete a brief 2-5-minute video phone call involving you, your parent / carer and a member of the research team, to confirm your eligibility. The call will be recorded as evidence of eligibility. After that call, we will invite you to complete the five-part study.

You will then be asked to complete the following:

1. Complete an online questionnaire asking you to answer questions about how you have felt in the last week generally, how you feel in social situations online and offline and the type of content you encounter. You will also be asked to complete a short cognitive task and upload screenshots of your total phone screentime and time on each application. This should take approximately 75 minutes to complete.
2. You will then be contacted via SMS three (3) times per day, for five (5) days (outside school hours) to fill in brief surveys (1-2 minutes) about how you are feeling, any online and offline social interactions you have had, and what type (if any) of online content you have encountered in the prior two hours.
3. You will be invited to complete the questionnaires and SMS questions again after 6-, 12-, and 18-months. At each time point, participants will also complete a short cognitive task (~5 min) and be asked to upload screenshots of their total phone screentime and time on each application.

The research team will contact you via email and/or SMS to remind you when to complete the next round of questionnaires. You will receive an initial reminder, and one follow up reminder. You can opt out of reminders and withdraw from the study at any time.

Costs and Remuneration: There are <u>no costs</u> associated with participating in this research project. Upon full and faithful completion of the study you will be reimbursed for each section completed in Amazon gift vouchers:

- For each of the four assessments (~75min each) you will receive A$22.50 (total A$90). You will also receive bonus payments if you complete all your follow-up assessments (Time 2-4) in full. If you complete the assessment at Time 2 (~6 months after Time 1) you will receive an extra A$5; for Time 3 (~12 months after Time 1) you will receive an extra A$10 and for Time 4 (~18 months after Time 1) an extra A$15. That means if you complete all four assessments in full you will receive a total of A$120.
- For each of the four multiple daily survey periods (3 SMS/day for 5 days) you will receive A$12.50 (each). Total of A$50.
- That is, for full and faithful study completion you will receive A$170.
- Faithful completion means that you cannot complete more than two of the attention checks incorrectly at any timepoint. Attention checks are questions that are inserted into the study to make sure you are paying attention to the study instructions.
- Additionally, you will be eligible for a further bonus payment of A$5 for each 5-day SMS period if you respond to each SMS prompt within one (1) hour. That is, you can earn an additional total of A$20.

Some questions may be upsetting. This information will be clearly stated before sensitive questions are presented. If you do not wish to answer a question or complete a task, you may skip it and go to the next question or stop immediately. If you experience discomfort or feelings of distress while participating in the research and you require support, you can stop participating at any time. You can also contact the research team with any questions or request to be contacted by the research team's clinical psychologist. Alternatively, a list of support services and their contact details are provided below.

6. **What are the possible benefits to participation?**
We hope to use information we get from this research study to contribute to our global understanding of the pros and cons of interacting online to help young people, parents and governments make the right decisions about how to manage, monitor and regulate young people's access to social media

7. **What will happen to information about me?**
By clicking the consent button, you consent to the research team collecting and using information about you for the research study. Information and recordings collected from you will be stored on a UNSW password protected One Drive only accessible to the approved research investigators. During the study, data will be stored in a re-identifiable format where any identifiers such as your name and email will be replaced with a unique code. The research team will take all reasonable steps to protect your privacy. After the analysis is complete, the research team will store the data collected from you for this research project for a minimum of 7 years after the publication of research results.

You will be asked to provide your consent for the research team to share or use information collected from you in future research. Your information will only be shared in a format that will not identify you. This fully anonymised data (that means you cannot be identified through any means) will be made available on an open access repository for other researchers who consent to adhering to ethical research practices.
The information you provides is personal information for the purposes of the Privacy and Personal Information Protection Act 1998 (NSW). You have the right of access to personal information held about you by the University, the right to request correction and amendment of it, and the right to make a complaint about a breach of the Information Protection Principles as contained in the PPIP Act. Further information on how the University protects personal information is available in the **UNSW Privacy Management Plan**.

8. **How and when will I find out what the results of the research study are?**
The research team intend to publish and report the results of the research. All Information will be published in a way that will not identify you.

If you would like to receive a copy of the results you can let the research team know by inserting your email or mailing address in the consent form. We will only use these details to send you the results of the research.

9. **What if I want to withdraw from the research study?**
If you do consent to participate, you may withdraw at any time. You can do so by contacting the research team and telling them that you no longer want to participate. Your decision not to participate or to withdraw from the study will not affect your relationship with UNSW, Sydney or any organisation helping with recruitment for this research e.g. your school or sports club. If you decide to leave the research study, the researchers will not collect additional information from you. You can request that any identifiable information about you be withdrawn from the research project.

10. **What should I do if I have further questions about my involvement in the research study?**
The person you may need to contact will depend on the nature of your query. If you require further information regarding this study or if you have any problems which may be related to your involvement in the study, you can contact the following member/s of the research team. We will aim to answer within two business days.

Research Team Contact Details

Lead Researcher

**Name**	Anna Bilston
**Position**	PhD Candidate
**Email**	a.bilston@unsw.edu.au

Chief Investigator

**Name**	Susanne Schweizer
**Position**	Scientia Associate Professor, School of Psychology, UNSW
**Telephone**	+61 2 9348 1896
**Email**	DASLab@unsw.edu.au<br>s.schweizer@unsw.edu.au

Support Services Contact Details
If at any stage during the study, you feel distressed or require additional support from someone not involved in the research please contact one of the following free, 24/7 support services. Many have text or online chat services if you are not comfortable or not able to call, as provided:

**Name/Organisation**	Lifeline
**Position**	Crisis counsellor
**Telephone**	13 11 14
**Text Support**	0477 13 11 14
**Online Chat**	https://www.lifeline.org.au/crisis-chat/

**Name/Organisation**	13YARN (Aboriginal
**Position**	Aboriginal or Torres Strait Islander Crisis Supporter
**Telephone**	13 92 76

**Name/Organisation**	1800RESPECT
**Position**	Sexual assault, family and domestic violence counsellor
**Telephone**	1800 737 732
**Text Support**	0458 737 732
**Online Chat link:**	https://www.1800respect.org.au/online-chat-1800respect

**Name/Organisation**	Beyond Blue
**Position**	Counsellor
**Telephone**	1300 22 4636
**Online Chat link:**	https://www.beyondblue.org.au/get-support/talk-to-a-counsellor/chat

**Name/Organisation**	Kids Helpline
**Position**	Counsellor
**Telephone**	1800 55 1800
**Online Chat link**	https://kidshelpline.com.au/get-help/webchat-counselling

11. **What if I have a complaint or any concerns about the research study?**
If you have a complaint regarding any aspect of the study or the way it is being conducted, please contact the UNSW Human Ethics Coordinator:

Complaints Contact

**Position**	UNSW Human Research Ethics Coordinator
**Telephone**	+ 61 2 9385 6222
**Email**	humanethics@unsw.edu.au
**HC Reference Number**	iRECS 9197

Consent Form

Declaration by the participant

By checking “I agree” below:

- I understand I am being asked to provide consent to participate in this research study;
- I have read the Participant Information Sheet, or it has been provided to me in a language that I understand;
- I understand the purposes, study tasks and risks of the research described in the study;
- I understand that the research team will video record the short screening interview; I agree to be recorded for this purpose.
- I provide my consent for the information collected about me to be used for the purpose of this research study.
- I consent for fully anonymised data (that means I cannot be identified by any means) to be made accessible to other researchers on an open access repository.
- I have had an opportunity to ask questions, and I am satisfied with the answers I have received.
- I freely agree to participate in this research study as described and understand that I am free to withdraw at any time during the study and withdrawal will not affect my relatinship with any of the named organisations and/or research team members
- I understand that the results of the research will be made available on the DAS Lab website

○ I DO NOT Agree

○ **I Agree**

Page Break

A_Ph

Display this question

If To make sure you are happy with participating in this research please take the time to read... I Agree Is Selected

What is your **mobile phone** number?

If filled, please check this is correct. This will only be used for notifications for surveys as outlined in the Participant Information Statement.

Mobile phone number

${e://Field/A_Ph}

Import from library | Add new question

Landing & Consent - A_Aus - No Phone

LP - A_No Phone

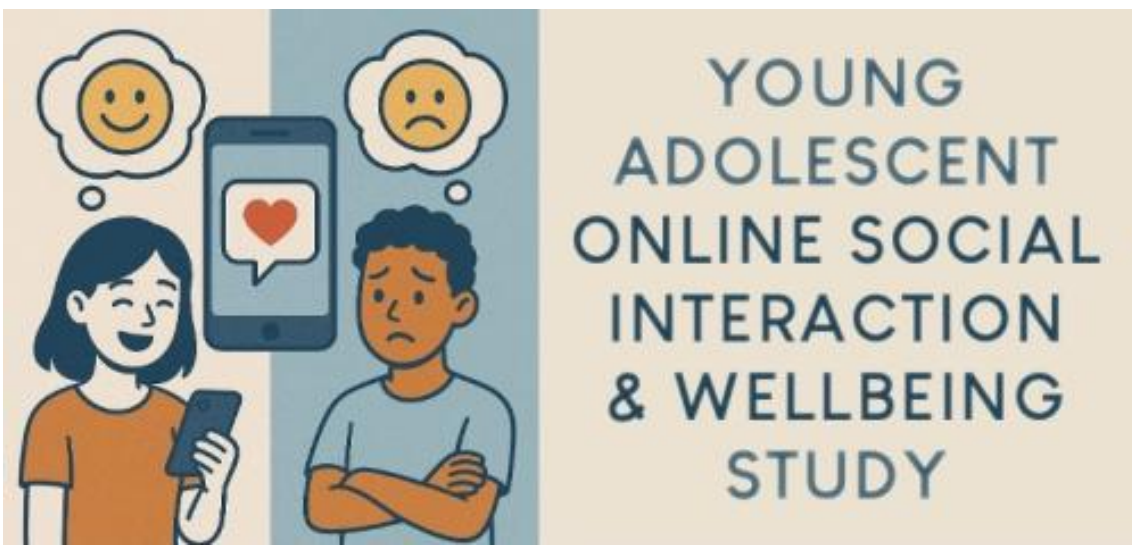

You are invited to participate in our study!

Is using social media and messaging a good or bad thing for young people?
For whom? Why?

We're looking for young people aged 10–15 years old to tell us about your experiences connecting online and in-person.

The study involves four parts over 18-months. Will ask you to complete questionnaires and a short cognitive task now and then in 6-, 12- and 18- months time.

Completing all parts of the study will earn you a **A$120** Amazon voucher.

Interested?

Page Break

PISCF - A_Aus - No P

To make sure you are happy with participating in this research please take the time to read this participant information statement and, if you'd like to participate indicate with "I agree" below:

This study has been approved by UNSW Human Research Ethics Committee (iRECS 9197) who check that the research we are conducting with you is useful and make sure your rights and privacy are protected.

1. **What is this research study about?**
   The research study aims to investigate the relationship between online social interactions and mental health, and what factors predict whether someone will find online social interactions beneficial or not. There has been lots of public interest in whether the rise in social media use is related to an increase in youth mental health problems, and we hope to use information we gain from this research study to contribute to our understanding of the pros and cons of interacting online.

2. **Who is conducting this research?**
   The study is being carried out by the following researchers: Scientia Associate Professor, Dr Susanne Schweizer (Chief Investigator), Anna Bilston (Lead Researcher / PhD Candidate), Associate Professor Dr Aliza Werner-Siedler (Black Dog Institute), Dr Lucy Zhang (Post Doctoral Researcher), Dr Lyndsay Brown, (Black Dog Institute), Dr Sarah Stevens (Black Dog Institute), Yasmin Hasan (Research Assistant), Emily Morgan (Research Assistant), Weike Wang (PhD Student), Xu Qi (Third Year Student) and Kirsten Hertog (Third Year Student) at the UNSW School of Psychology.
   Research Funder: This research is being funded by the Australian Research Council.

3. **Inclusion/Exclusion Criteria**
   Before you decide to participate in this research study, we need to ensure that it is ok for you to take part. The research study is looking recruit people who meet the following criteria:
   - The young person needs to be 10-15 years old
   - The young person and parent/carer need to have very good command of written and spoken English
   - Live in Australia, the UK, or NZ

4. **Do I have to take part in this research study?**
   Participation in this research study is **voluntary**. If you do not want to take part, you do not have to. If you agree to take part and later change your mind, you are free to withdraw yourself from the study at any stage.
   If you decide you want to take part in the research study, you will be asked to:
   - Read this study information carefully (ask questions if necessary).
   - Provide consent at the bottom of this page if you decide to participate in the study.
   - Keep a copy of this form.

5. **What does participation in this research require, and are there any risks involved?**
   If you agree to participate, we will first ask you to complete a brief 2-5-minute video phone call involving you, your parent / carer and a member of the research team, to confirm your eligibility. The call will be recorded as evidence of eligibility. After that call, we will invite you to complete the five-part study.

   You will then be asked to complete the following:
   1. Complete an online questionnaire asking you to answer questions about how you have felt in the last week generally, how you feel in social situations online and offline and the type of content you encounter. You will also be asked to complete a short cognitive task. This should take approximately 75 minutes to complete.
   2. You will be invited to complete the questionnaires again after 6-, 12-, and 18-months.

   The research team will contact you via email to remind you when to complete the next round of questionnaires. You will receive an initial reminder, and one follow up reminder. You can opt out of reminders and withdraw from the study at any time.

   Costs and Remuneration: There are no costs associated with participating in this research project. Upon full and faithful completion of the study you will be reimbursed for each section completed in Amazon gift vouchers:
   - For each of the four assessments (~75min each) you will receive A$22.50 (total A$90). You will also receive bonus payments if you complete all your follow-up assessments (Time 2-4) in full. If you complete the assessment at Time 2 (~6 months after Time 1) you will receive an extra A$5; for Time 3 (~12 months after Time 1) you will receive an extra A$10 and for Time 4 (~18 months after Time 1) an extra A$15.
   - That is, for full and faithful study completion you will receive A$120.
   - Faithful completion means that you cannot complete more than two of the attention checks incorrectly at any timepoint. Attention checks are questions that are inserted into the study to make sure you are paying attention to the study instructions.

   Some questions may be upsetting. This information will be clearly stated before sensitive questions are presented. If you do not wish to answer a question or complete a task, you may skip it and go to the next question or stop immediately. If you experience discomfort or feelings of distress while participating in the research and you require support, you can stop participating at any time. You can also contact the research team with any questions or request to be contacted by the research team's clinical psychologist. Alternatively, a list of support services and their contact details are provided below.

6. **What are the possible benefits to participation?**
   We hope to use information we get from this research study to contribute to our global understanding of the pros and cons of interacting online to help young people, parents and governments make the right decisions about how to manage, monitor and regulate young people's access to social media

7. **What will happen to information about me?**
   By clicking the consent button, you consent to the research team collecting and using information about you for the research study. Information and recordings collected from you will be stored on a UNSW password protected One Drive only accessible to the approved research investigators. During the study, data will be stored in a re-identifiable format where any identifiers such as your name and email will be replaced with a unique code. The research team will take all reasonable steps to protect your privacy. After the analysis is complete, the research team will store the data collected from you for this research project for a minimum of 7 years after the publication of research results.

   You will be asked to provide your consent for the research team to share or use information collected from you in future research. Your information will only be shared in a format that will not

identify you. This fully anonymised data (that means you cannot be identified through any means) will be made available on an open access repository for other researchers who consent to adhering to ethical research practices.

The information you provide is personal information for the purposes of the Privacy and Personal Information Protection Act 1998 (NSW). You have the right of access to personal information held about you by the University, the right to request correction and amendment of it, and the right to make a complaint about a breach of the Information Protection Principles as contained in the PPIP Act. Further information on how the University protects personal information is available in the UNSW Privacy Management Plan

8. How and when will I find out what the results of the research study are?
The research team intend to publish and report the results of the research. All Information will be published in a way that will not identify you.

If you would like to receive a copy of the results you can let the research team know by inserting your email or mailing address in the consent form. We will only use these details to send you the results of the research.

9. What if I want to withdraw from the research study?
If you do consent to participate, you may withdraw at any time. You can do so by contacting the research team and telling them that you no longer want to participate. Your decision not to participate or to withdraw from the study will not affect your relationship with UNSW, Sydney or any organisation helping with recruitment for this research e.g. your school or sports club. If you decide to leave the research study, the researchers will not collect additional information from you. You can request that any identifiable information about you be withdrawn from the research project.

10. What should I do if I have further questions about my involvement in the research study?
The person you may need to contact will depend on the nature of your query. If you require further information regarding this study or if you have any problems which may be related to your involvement in the study, you can contact the following member/s of the research team. We will aim to answer within two business days.

Research Team Contact Details

Lead Researcher

**Name**	Anna Bilston
**Position**	PhD Candidate
**Email**	a.bilston@unsw.edu.au

Chief Investigator

**Name**	Susanne Schweizer
**Position**	Scientia Associate Professor, School of Psychology, UNSW
**Telephone**	+61 2 9348 1896
**Email**	DASLab@unsw.edu.au<br>s.schweizer@unsw.edu.au

Support Services Contact Details
If at any stage during the study, you feel distressed or require additional support from someone not involved in the research please contact one of the following free, 24/7 support services. Many have text or online chat services if you are not comfortable or not able to call, as provided:

**Name/Organisation**	Lifeline
**Position**	Crisis counsellor
**Telephone**	13 11 14
**Text Support**	0477 13 11 14

Consent Form

Declaration by the participant

By checking "I agree" below:

- I understand I am being asked to provide consent to participate in this research study;
- I have read the Participant Information Sheet, or it has been provided to me in a language that I understand;
- I understand the purposes, study tasks and risks of the research described in the study;
- I understand that the research team will video record the short screening interview; I agree to be recorded for this purpose.
- I provide my consent for the information collected about me to be used for the purpose of this research study.
- I consent for fully anonymised data (that means I cannot be identified by any means) to be made accessible to other researchers on an open access repository.
- I have had an opportunity to ask questions, and I am satisfied with the answers I have received.
- I freely agree to participate in this research study as described and understand that I am free to withdraw at any time during the study and withdrawal will not affect my relatinship with any of the named organisations and/or research team members
- I understand that the results of the research will be made available on the DAS Lab website

○ I DO NOT Agree

○ **I Agree**

Page Break

Import from library

Add new question

A_Email Details

A_Email

What is your **email**?

Please complete if no auto-fill. This is needed to send a voucher as compensation when the study is completed.

Email

${e://Field/A_Email}

Page Break

Results

Would you like to receive the results of the study when complete?

- ☐ No - thanks, but I am not interested in the results
- ☐ Yes - please send the results to my email address (provided above)
- ☐ Yes - please send the results to the following postal address:

Import from library | Add new question

Screenshots

Phone Type

Do you have an iPhone or Android phone?

- ○ iPhone
- ○ Android

Page Break

DT - iPhone

Display this question

If Do you have an iPhone or Android phone? iPhone Is Selected

Please enter your **Daily Average Phone Screen Time** for the most recent **FULL WEEK** (Sunday to Saturday) by carefully ***following these instructions***:

1) Go to Settings

2) Go to Screen Time

3) Tap "See All App & Website Activity

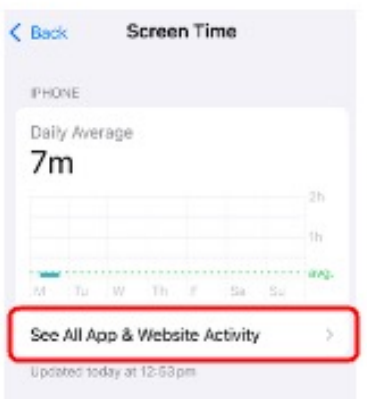

4) Check the report is for your phone time ONLY (i.e. says "iPhone or similar, NOT All Devices…)

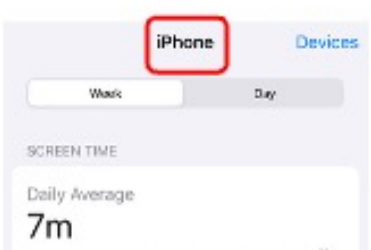

If it doesn't say "iPhone" or says "All Devices" at the top of the screen and the blue "Devices" is visible in the top right hand corner ….

Click on "Devices" at the top left and select your phone from your list of devices

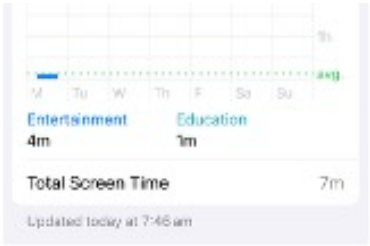

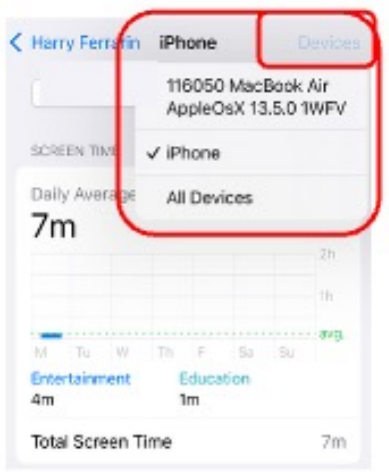

5) Tap **"Week"** to see a summary of your weekly usage

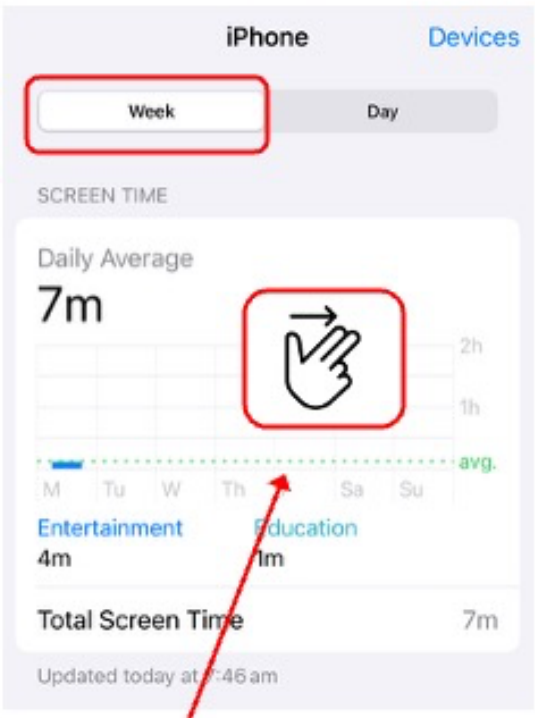

6) Swipe Right to get a full **7-day view** with "Last Week's Average"

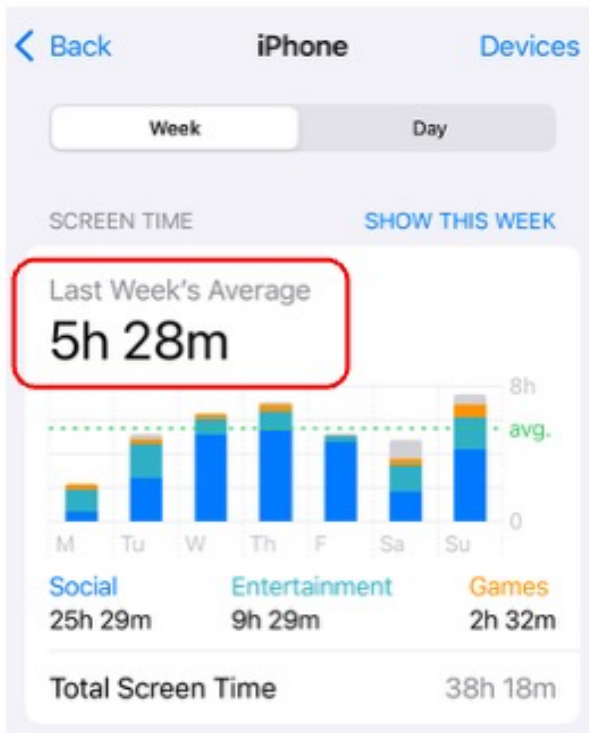

7) Please record the **average daily hours and minutes from your phone** below:

Hours

Minutes

Page Break

SS #1 - iPhone

Display this question

If Do you have an iPhone or Android phone? iPhone Is Selected

8) Now, take a **screenshot of this same page** with Last Week's Average Screentime **= (Screenshot #1)**

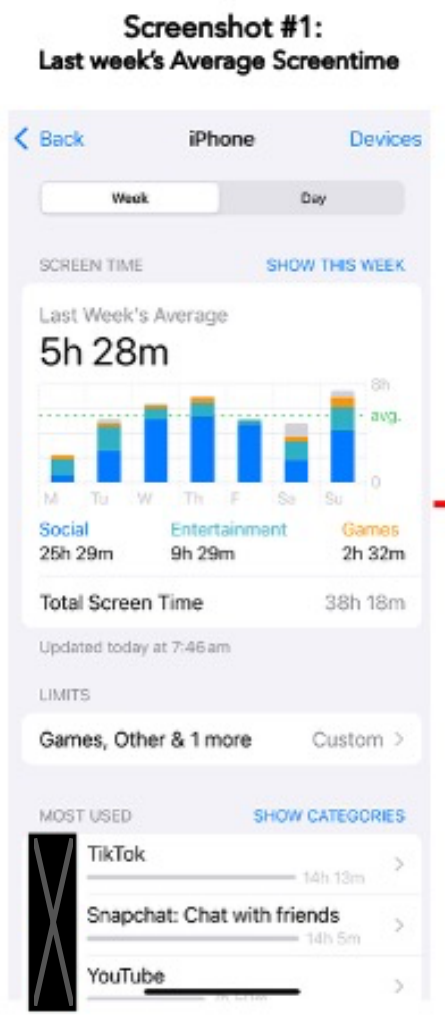

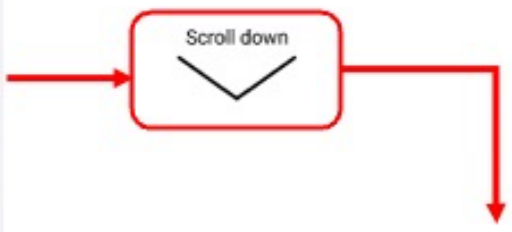

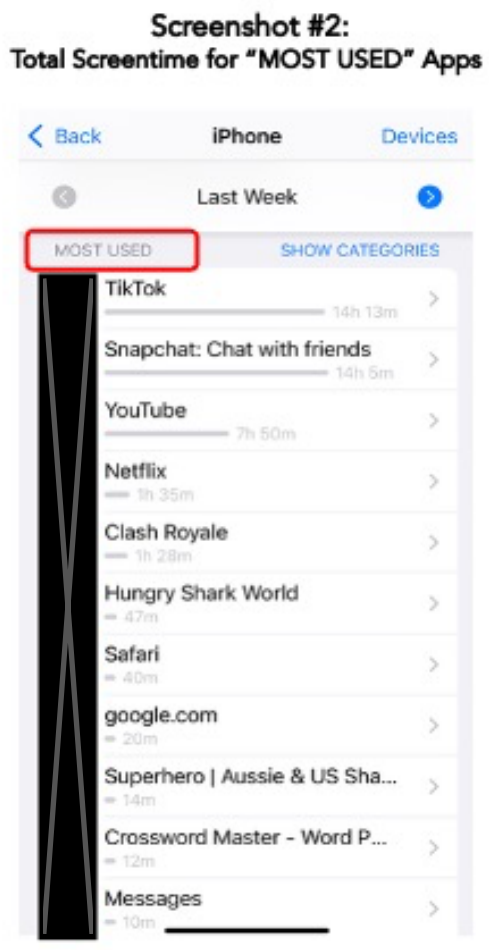

9) Next, **SCROLL DOWN** to **fill the screen** with your **most used apps** for the last week

Leave "MOST USED" in the shot and take another screenshot = **(Screenshot #2)**

10) Please upload these screenshots below:

Screenshot #1

Choose File no file selected

iPhone SS #2

Display this question

If Do you have an iPhone or Android phone? iPhone Is Selected

Screenshot #2

Choose File no file selected

Page Break

DT - Android

Display this question

If Do you have an iPhone or Android phone? Android Is Selected

Please enter your **Daily Average Phone Screen Time** for the most recent **FULL WEEK** (Sunday to Saturday) by carefully ***following these instructions***:

1) Open the Settings App 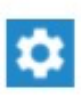

2) Select "Digital Wellbeing and Parental Controls"

- If you don't see this option, tap User & accounts, and then turn on "Show parental controls."

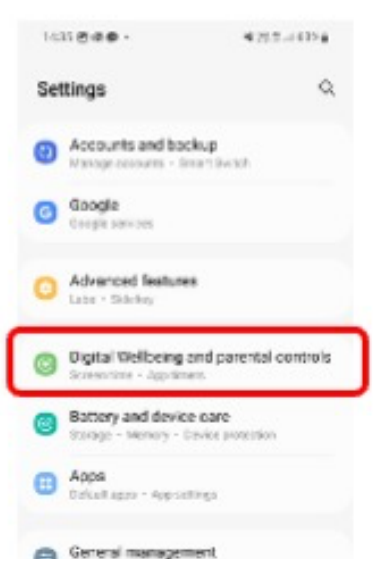

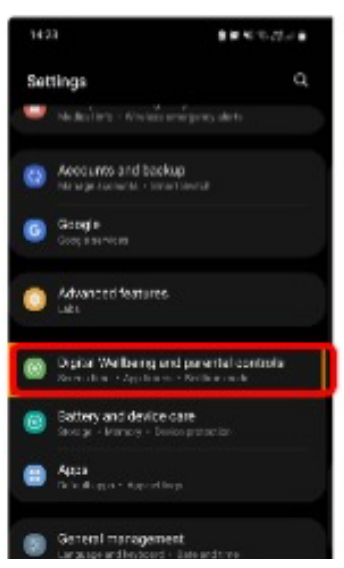

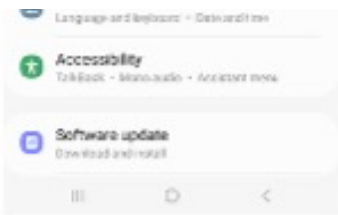

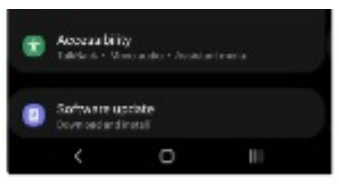

3) Tap Graph icon or “Dashboard”

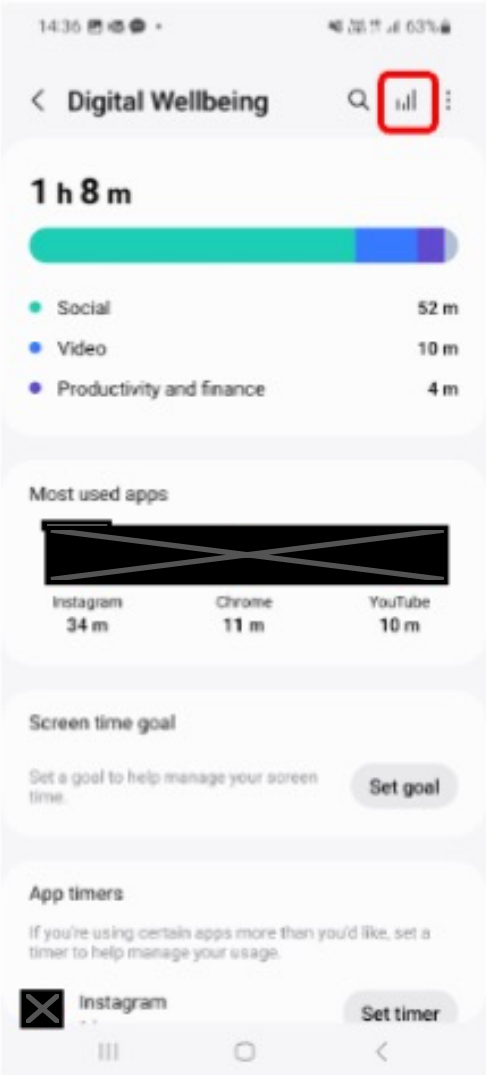

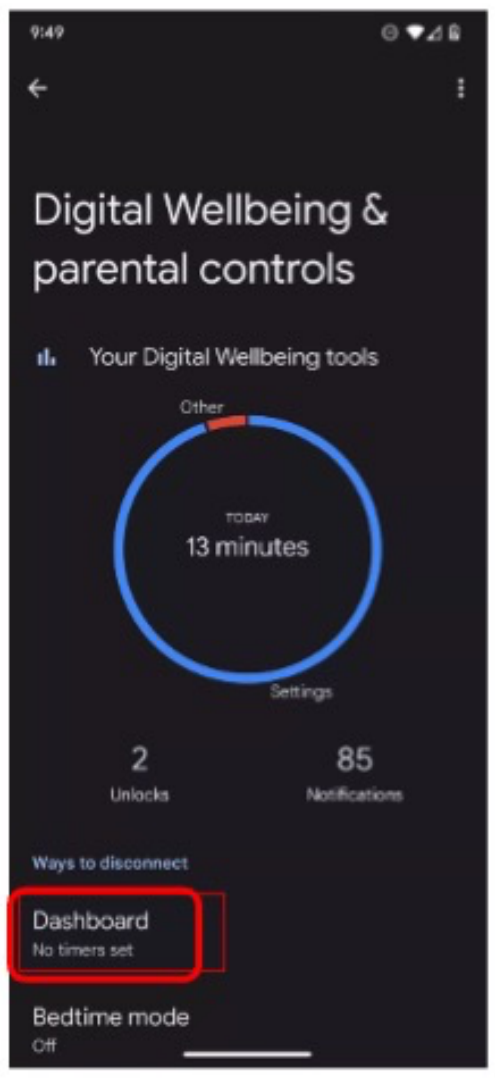

4) Check the graph shows a “Weekly report” / the **last <u>complete week</u>** ***(7-days)***:

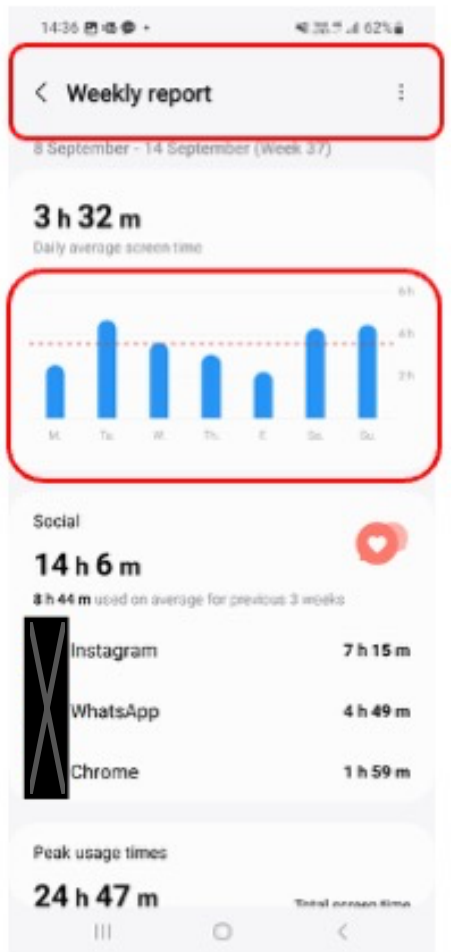

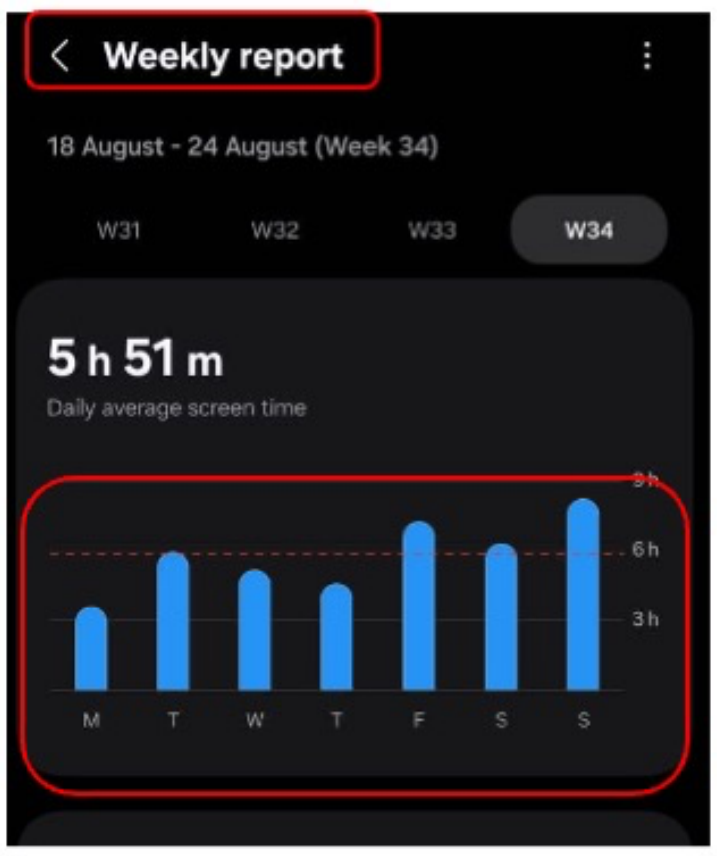

4) ***Please record you <u>Daily Average Screen Time</u> for the last complete week (7-days):***

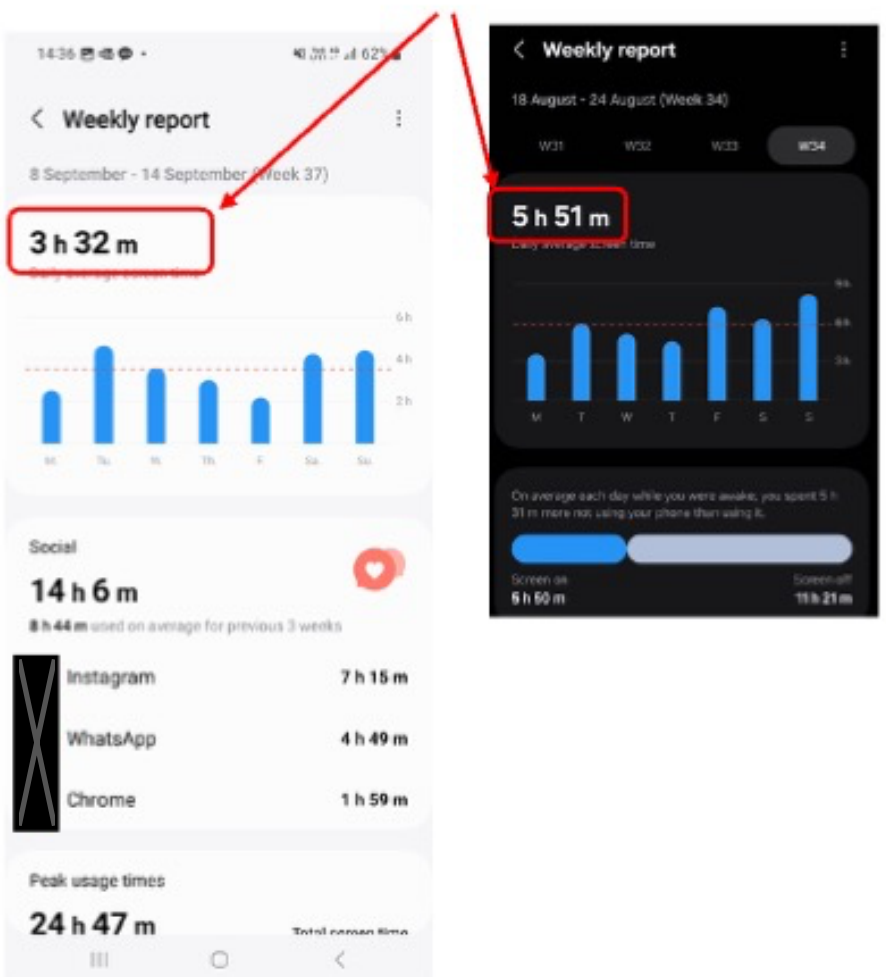

Hours

[ ]

Minutes

Page Break

SS#1 - Android

Display this question

If Do you have an iPhone or Android phone? Android Is Selected

6) While showing the same “Weekly Report” screen,

a. **TAKE A SCREEN SHOT(s)** of ***<u>all the information provided</u> (as per the images)***
b. Send them to yourself and
c. Upload below

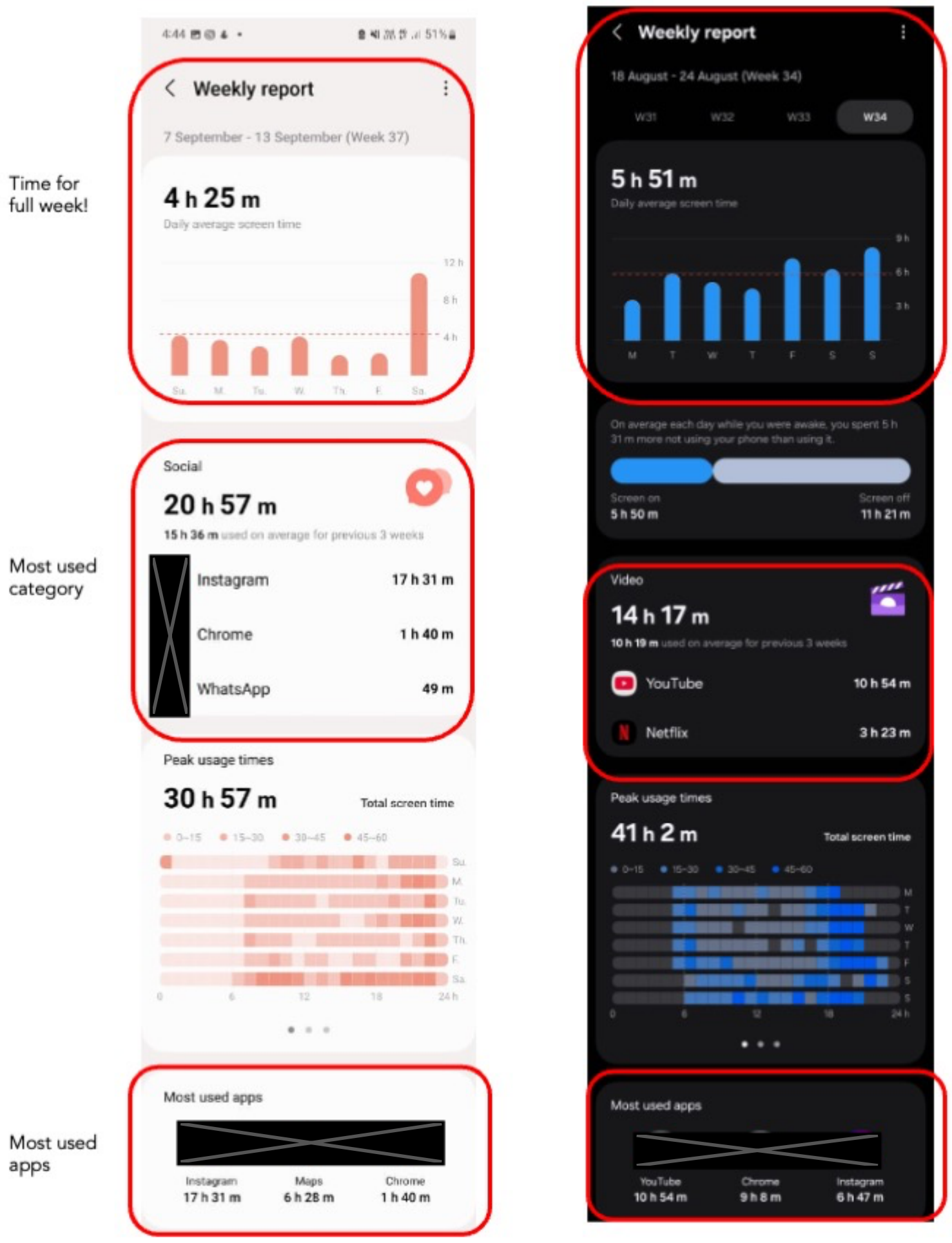

Note: To get the whole image at once, after taking a screenshot, you can tap

- **Capture more** (on Pixels with Android 12

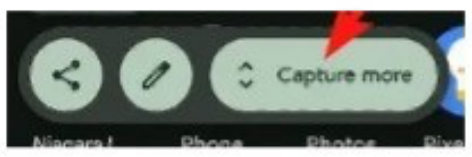

Or

- **Scroll capture** (on Samsung)

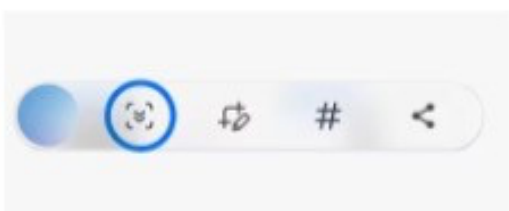

- Otherwise take 2-3 screenshots to capture

Screenshot #1

Choose File no file selected

SS#2

Display this question

If Do you have an iPhone or Android phone? Android Is Selected

Screenshot #2

Choose File no file selected

SS#3

Display this question

If Do you have an iPhone or Android phone? Android Is Selected

Screenshot #3 (if needed)

Choose File no file selected

SS#4

Display this question

If Do you have an iPhone or Android phone? Android Is Selected

Screenshot #4 (if needed)

Choose File no file selected

Import from library | Add new question

Demographics

Age

What is your **age** in years?

15

Page Break

Gender

Please select which **gender** you identify with:

- Female
- Male
- Non-binary / third gender
- I use a different term:
- Prefer not to say

Page Break

Ethnicity

Please select which **ethnicity** you identify with:

- Aboriginal or Torres Strait Islander
- Maori
- Black
- Asian

- White
- Hispanic
- Mixed
- Other
- Prefer not to say

Import from library | Add new question

AC 1

AC 1

For this question, please select the word that is not a fruit in the list below:

- Apple
- Pear
- Hammer
- Banana
- Orange

Import from library | Add new question

OSI Intro

OSI Intro

Some of the following questions refer to social media and messaging. Examples are:

Social media
e.g. Facebook, Instagram, X, TikTok, Snapchat

[redacted]

and

Messaging
e.g. Messages, WhatsApp, Facebook/Kids Messenger, Telegram, Discord, WeChat

[redacted]

For the purpose of this study, ***You Tube, other internet and gaming sites*** are **NOT** considered ***social media or messaging***.

Import from library | Add new question

Online Social Interaction

SM - ever used

Have you **ever used** **social media?** (e.g. Facebook, Instagram, X, TikTok, Snapchat, etc)

- Yes
- No
- Prefer not to say

Page Break

SM - first use

Display this question

If Have you ever used social media? (e.g. Facebook, Instagram, X, TikTok, Snapchat, etc) Yes Is Selected

What **age** were you when you **first used** **social media**?

15

Page Break

SM - # used

Display this question

If Have you ever used social media? (e.g. Facebook, Instagram, X, TikTok, Snapchat, etc) Yes Is Selected

How many **social media** applications do you use **once a week or more**?

(e.g. Snapchat only = 1, Snapchat & Instagram = 2, Snapchat & Instagram & Facebook =3, etc)

0

Page Break

TX - ever

Have you **ever used** a **messaging** application (e.g. Messages, WhatsApp, Facebook Messenger, Kids Messenger, Telegram, Discord, WeChat, etc)?

- Yes
- No
- Prefer not to say

Page Break

TX - first use

Display this question

If Have you ever used a messaging application (e.g. Messages, WhatsApp, Facebook Messenger, Kids Mes... Yes Is Selected

What **age** were you when you **first used** **messaging**?

15

Page Break

Own accounts

Do you have your **own account/profile** that you currently use for ...

	Yes	No	Don't Know	Prefer not to say
**Social Media?** (e.g., Facebook, Instagram, X, TikTok, Snapchat, etc)	○	○	○	○
**Messaging?** (e.g., Messages, WhatsApp, Facebook Messenger, Kids Messenger, Telegram, Discord, WeChat)	○	○	○	○

Gaming?
(e.g., Fortnite, Roblox)

Page Break

SM accounts

Display this question

If Do you have your own account/profile that you currently use for ... Social Media?
 (e.g., Facebook, Instagram, X, TikTok, Snapchat, etc) - Yes Is Selected

On **which** of the following **social media** applications do you have your **own account / profile**?

- Facebook
- Instagram
- X
- TikTok
- SnapChat
- Other(s) (please specify:)
- Prefer not to say

Page Break

SM Use type

Display this question

If Do you have your own account/profile that you currently use for ... Social Media?
 (e.g., Facebook, Instagram, X, TikTok, Snapchat, etc) - Yes Is Selected

When using **social media**, do you **spend more time** ...

- Browsing
- Sharing, posting or liking
- Time spent equally
- Prefer not to say

Page Break

TX sites

Display this question

If Do you have your own account/profile that you currently use for ... Messaging?
 (e.g., Messages, WhatsApp, Facebook Messenger, Kids Messenger, Telegram, Discord, WeChat) - Yes Is Selected

On **which** of the following **messaging** applications do you have your **own account / profile**?

- Messages
- WhatsApp
- Facebook Messenger
- Kids Messenger
- Telegram
- Discord
- WeChat
- Other(s) (please specify)
- Prefer not to say

Import from library | Add new question

Social Media Mindset

SM Mindset

For this question, we want you to think about the **ways _you_ think about and use social media**.

For each series of statements, please rate the extent to which you agree with each statement.

	Strongly Disagree	Disagree	Neither Agree nor Disagree	Agree	Strongly agree	Not Applicable
Using social media is meaningful for me	○	○	○	○	○	○
Using social media is a waste of time for me	○	○	○	○	○	○
Using social media is fun and enjoyable for me	○	○	○	○	○	○
Using social media strengthens and supports my relationships	○	○	○	○	○	○
Social media is a helpful tool that I use	○	○	○	○	○	○
I'm good at managing the ways I use social media	○	○	○	○	○	○
I'm in control of how I use social media	○	○	○	○	○	○
I end up using social media even when I don't mean to	○	○	○	○	○	○
I find it hard to resist the pull of social media	○	○	○	○	○	○
I am often manipulated by social media	○	○	○	○	○	○

Import from library | Add new question

Internet Permission and Behaviours

Internet use

These questions are about what your parent/carer allows you to do on the internet and if so, whether you need their permission to do them each time. (Not whether / how often you might do it)

How often **_are you allowed_ by your parent/carer(s) to ...**

	Anytime	Only with permission or supervision	Never	I don't know	Prefer not to say
Use a web or phone camera (e.g., for Facetime / other video chat)	○	○	○	○	○
Download music or films	○	○	○	○	○
Use a social networking site (e.g., Facebook, Snapchat, Instagram, Twitter)	○	○	○	○	○
Watch video clips (e.g., on YouTube)	○	○	○	○	○
Play games with other people online	○	○	○	○	○
Read/watch news online	○	○	○	○	○
Use the internet for school / work	○	○	○	○	○
Spend time in a virtual world (e.g. Minecraft)	○	○	○	○	○
Share photos or videos of _myself_	○	○	○	○	○
Share photos, videos or music of _other people_	○	○	○	○	○

Page Break

Parent knowledge

How much do you think your **_parent/carer knows_** about **_what you do_** on the internet**?**

- ○ Nothing
- ○ Just a little
- ○ Quite a bit

- A lot
- I don't know
- Prefer not to say

Import from library | Add new question

AC 2

AC 2

You're doing great!

For this question, please select the word "pumpkin" from the list of vegetables below:

- Broccoli
- Pumpkin
- Carrot
- Lettuce
- Cauliflower

Import from library | Add new question

Aus Ban

Aus Ban - aware

Before this question, **did you know that the Government is banning social media accounts for people under 16 years** of age?

- Yes
- No
- Prefer not to say

Page Break

Aus ban - changes

Display this question

If Before this question, did you know that the Government is banning social media accounts for peopl... Yes Is Selected

Has knowing that there will be a ban **changed your social media use already?**

- Yes, I deleted my account(s) and chose to stop using social media based on this
- Yes, I deleted my account(s) but I still browse social media anonymously
- No, I still have a social media account(s)
- No, I don't have a social media account(s) but I still browse social media anonymously
- No, I don't use social media anyway
- Prefer not to say

Page Break

Aus ban - to change

When it starts, **how will the Government ban** on accounts for under-16-year-olds **change your social media** usage?

- I will stop using social media completely
- I will still browse social media without an account
- I will find a way to maintain an account and still use it
- I will be 16 years old by then and will keep using my social media account
- I don't use social media so nothing will change

Prefer not to say

Import from library | Add new question

Harmful Content

HC - Warning

There are lots of great things to do on social media that many young people enjoy, but there are also some things that are not always good. Some young people can find some of these upsetting, while others might not get upset.

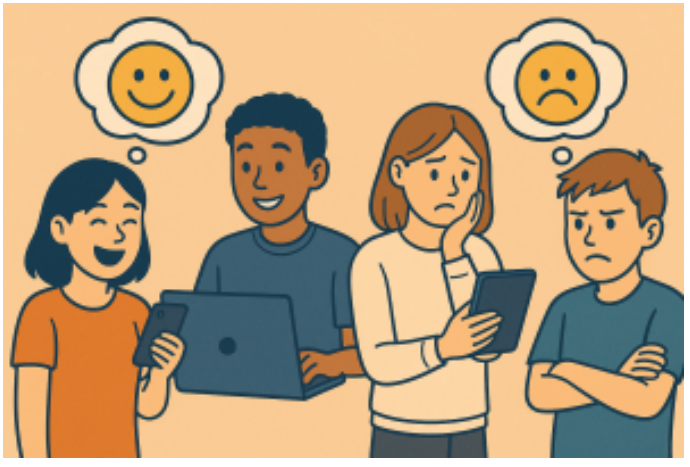

We want to learn about the things that can upset young people online so we can help them avoid these things, or deal with them better.

REMEMBER, *if you find any of these questions uncomfortable or distressing you don't have to answer them – mark "Prefer not to say", or you can discontinue by shutting the browser at any time.*

Page Break

HC - Stranger Online

Have you ever had **contact on social media** or **messaging** with someone you or your family/a close friend have **not met face-to-face** before?

- Yes
- No
- Prefer not to say

Page Break

HC - Y Stranger F2F

Display this question

If Have you ever had contact on social media or messaging with someone you or your family/a close fr... Yes Is Selected

In the ***past year***, have you ever **met anyone face-to-face** that you **first met** on **social media** or **messaging**?

- Yes
- No
- Prefer not to say

Page Break

HC - Y Stranger feel

Display this question

If In the past year, have you ever met anyone face-to-face that you first met on social media or mes... Yes Is Selected

Thinking of the ***last time*** you met anyone face-to-face that you first got to know on **social media** or **messaging**, **how did you feel** about it?

- I was happy
- I was not happy or upset
- I was a little upset
- I was fairly upset

I was very upset

Prefer not to say

Page Break

Bullying - desc

Sometimes people say or do hurtful or nasty things to someone and this can often be quite a few times on different days over a period of time.

This can include
- teasing someone in a way the person does not like
- leaving someone out of things

When people are hurtful or nasty to someone in this way, it can happen
- face-to-face (in-person)
- by mobile phone (text, calls, video clips)
- on the internet (e-mail, messaging, social media, chatrooms)

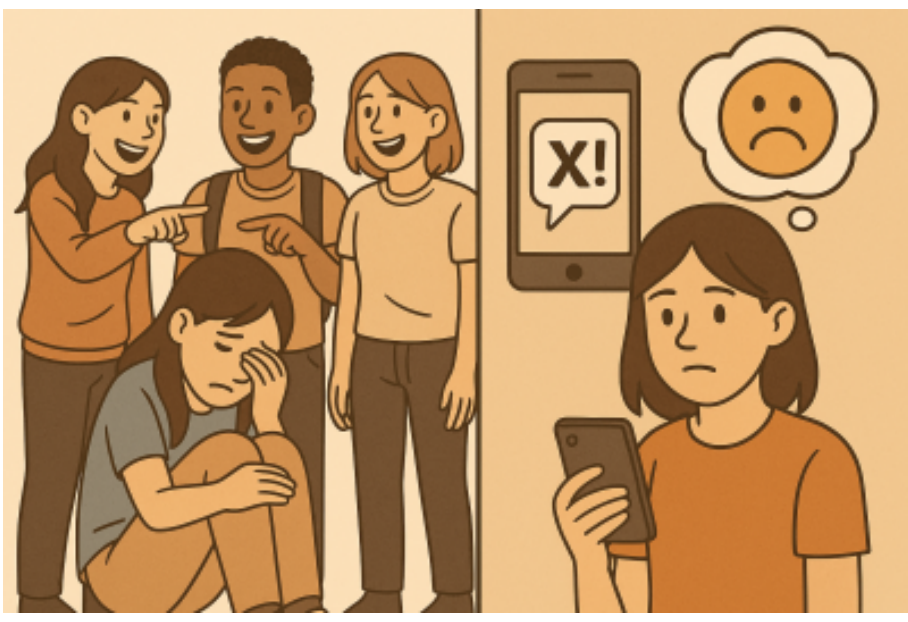

The following questions ask about these types of situations.

Remember, all answers are confidential (no one will link your answers to you) and you can leave the survey at any time.

Page Break

Bullying - victim

Over the ***past year***, has anyone ever **treated you in a hurtful or nasty way** in any of the following ways?

	Never / Almost Never	At least a few times every month	At least every week	Daily or almost daily	I don't know	Prefer not to say
In-person / face-to-face	○	○	○	○	○	○
Via social media	○	○	○	○	○	○
Via messaging	○	○	○	○	○	○
In an online game	○	○	○	○	○	○
Some other way	○	○	○	○	○	○

Page Break

Bullied - feelings

Display this question

If Over the past year, has anyone ever treated you in a hurtful or nasty way in any of the following... Via social media - Never / Almost Never Is Not Selected

Or Over the past year, has anyone ever treated you in a hurtful or nasty way in any of the following... Via messaging - Never / Almost Never Is Not Selected

Or Over the past year, has anyone ever treated you in a hurtful or nasty way in any of the following... In an online game - Never / Almost Never Is Not Selected

Thinking of the ***last time*** someone treated you in a hurtful or nasty way **online** **how did you feel?**

- I was not upset
- I was a little upset
- I was fairly upset
- I was very upset
- I don't know
- Prefer not to say

Page Break

Bully - perpetrator

In the ***past year***, have **you ever treated someone else in a hurtful or nasty way** in any of the following ways?

	Never / Almost Never	At least a few times every month	At least every week	Daily or almost daily	I don't know	Prefer not to say
In-person (face-to-face)	◯	◯	◯	◯	◯	◯
Via social media	◯	◯	◯	◯	◯	◯
Via messaging	◯	◯	◯	◯	◯	◯
In an online game	◯	◯	◯	◯	◯	◯
Some other way	◯	◯	◯	◯	◯	◯

Page Break

Sexual - warning

In the past year, you have seen lots of different images – pictures, photos, videos.

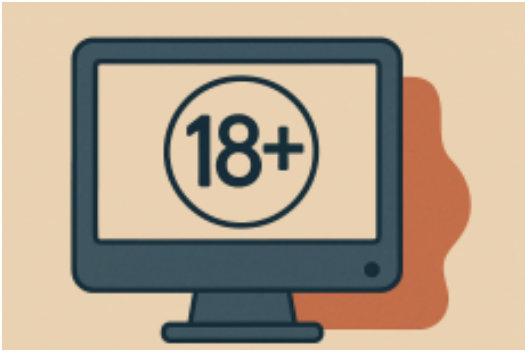

Sometimes, these images might be obviously sexual, e.g., they may show people naked or people having sex.

You might have never seen anything like this, or you may have seen something like this on a mobile phone, in a magazine, on the TV, on a DVD or on the internet.

The next few questions ask you about things like this.

Page Break

Sexual - seen

In the ***past year***, have you **ever seen any sexual images** (not including via school or home education on puberty and sexuality)?

◯ Yes
◯ No
◯ I don't know
◯ Prefer not to say

Page Break

Sexual - locn / freq

Display this question

If In the past year, have you ever seen any sexual images (not including via school or home educatio... Yes Is Selected

In the ***past year***, **how often** have you seen images of this kind in any of the **following ways**?

	Never / Almost Never	At least every month	At least every week	Daily or almost daily	I don't know	Prefer not to say
In a magazine or book	◯	◯	◯	◯	◯	◯
On television, film	◯	◯	◯	◯	◯	◯
On an online video sharing platform/site (e.g. YouTube)	◯	◯	◯	◯	◯	◯
Via social media (e.g. Facebook, TickTok, Instagram)	◯	◯	◯	◯	◯	◯

Via a messaging app (e.g. WhatsApp, Messenger. Text)						
In an online game						
On a pornographic website (adult/X-rated website)						
By a message sent directly to me via my computer / email						
By pop-ups on the internet (unrequested windows that appear during web surfing)						
Some other way						

Page Break

Sexual - feelings

Display this question

If In the past year, have you ever seen any sexual images (not including via school or home educatio... Yes Is Selected

Thinking of the ***last time*** you saw images of this kind, **how did you feel** about it?

- I was happy
- I was not happy or upset
- I as a little upset
- I was fairly upset
- I was very upset
- I don't know
- Prefer not to say

Page Break

Physical harm - warn

Online, people discuss things that may not be good for you. Here are some questions about these kinds of things.

In the ***past year***, have you seen **social media** or **messaging** content **where people talk about or show any of these things?:**

	Never / Almost Never	At least every month	At least every week	Daily or almost daily	Prefer not to say
**Ways to be very thin** (such as being anorexic or bulimic, or "thinspiration")					
**Hate messages that attack certain groups or individuals** (e.g., people of different colour, religion, nationality, or sexuality)					
**Experiences of drinking alcohol or taking illegal drugs**					
**Gory or violent images**, for example of people hurting other people or animals					

Page Break

Phys harm - intent

Display this question

If Online, people discuss things that may not be good for you. Here are some questions about these k... - Never / Almost Never Is Less Than 4

The ***last time*** you saw such content, was it **your intention to see it?**

- Yes
- No
- Prefer not to say

Page Break

No consent

Sometimes, **other people create posts or talk about us online** with or without our permission.

In the ***past year***, how often have the following things happened to you?

	Never / Almost Never	At least every month	At least every week	Daily or almost daily	I don't know	Prefer not to say
My parent/carer published information (such as text, pictures or movies) about me on the internet without asking first if I was OK with it	○	○	○	○	○	○
I received negative or hurtful comments from someone because of something my parent/carer published online	○	○	○	○	○	○
I asked my parent/carer to remove things they had published on the internet	○	○	○	○	○	○
I was upset because of information my parents published online	○	○	○	○	○	○
My teacher(s) published information about me on the internet without asking first if I was OK with it	○	○	○	○	○	○
My friend(s) published information about me on the internet without asking first if I was OK with it	○	○	○	○	○	○

Import from library | Add new question

AC 3

AC3

Whoa! So many questions, we know! You're more than half way done now!

Which of these things does not move:

- Car
- Plane
- Tram
- Statue
- Bike

Import from library | Add new question

MH and Individual Differences Intro

MH/ID Intro

The **next questions** are about **how you feel and think about things**.

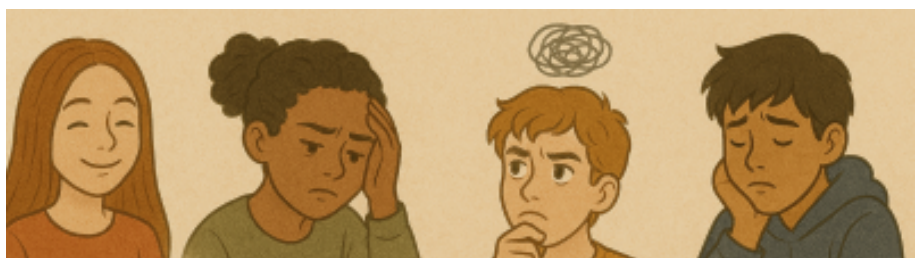

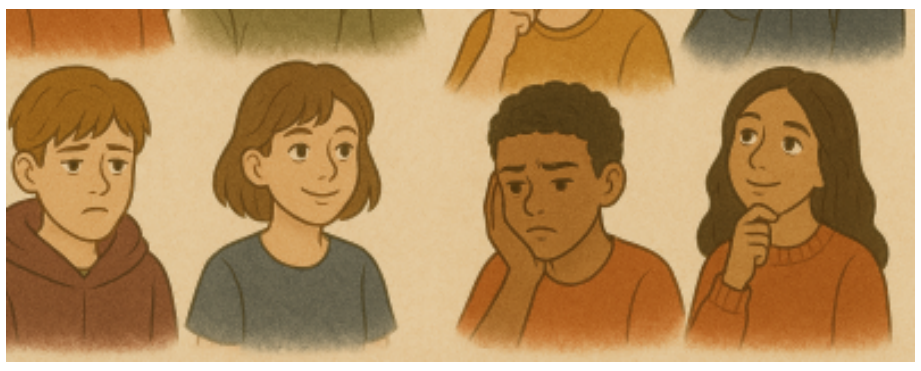

There are **no right or wrong answers**.

Import from library | Add new question

Mental Health

GAD-7

How often have you been **bothered by each of the following problems** during the ***past two weeks*?**

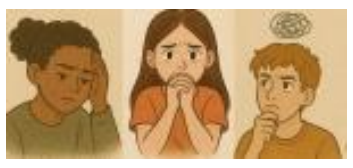

	Not at all	Several Days	More than half the days	Nearly every day
1. Feeling nervous, anxious or on edge	○	○	○	○
2. Not being able to stop or control worrying	○	○	○	○
3. Worrying too much about different things	○	○	○	○
4. Trouble relaxing	○	○	○	○
5. Being so restless it is hard to sit still	○	○	○	○
6. Becoming easily annoyed or irritable	○	○	○	○
7. Feeling afraid as if something awful might happen	○	○	○	○

Page Break

PHQ

How often have you been **bothered by each of the following symptoms** during the ***past two weeks*?**

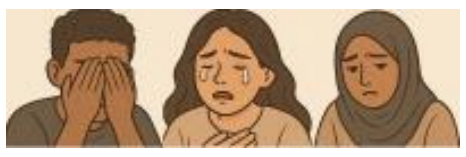

	Not at all	Several days	More than half the days	Nearly every day
1. Little interest in pleasure or doing things	○	○	○	○
2. Feeling down, depressed, irritable or hopeless	○	○	○	○
3. Trouble falling asleep, staying asleep or sleeping too much	○	○	○	○
4. Feeling tired or having little energy	○	○	○	○
5. Poor appetite, weight loss or overeating	○	○	○	○
6. Feeling bad about yourself – or that you are a failure or have let yourself or your family down	○	○	○	○
7. Trouble concentrating on things, such as school work, reading or watching television	○	○	○	○
8. Moving or speaking so slowly that other people could have noticed? Or the opposite – being so fidgety or restless that you have been moving around a lot more than usual	○	○	○	○

Import from library | Add new question

Wellbeing

WEMWB

Below are some more statements about **feelings and thoughts**. Please tick the box that best describes your experience of each over the ***last two weeks.***

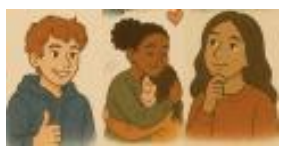

	None of the time	Rarely	Some of the time	Often	All of the time
I've been feeling optimistic about the future	○	○	○	○	○
I've been feeling useful	○	○	○	○	○
I've been feeling relaxed	○	○	○	○	○
I've been dealing with problems well	○	○	○	○	○
I've been thinking clearly	○	○	○	○	○
I've been feeling close to other people	○	○	○	○	○
I've been able to make up my own mind about things	○	○	○	○	○

Import from library | Add new question

Eating Disorders

EDQ-1

The following questions are about your **thoughts and feelings** about eating and your body in the ***past two weeks* *(14 days) only*.**

	No days	1-2 days	3-6 days	7 days	8-10 days	12-13 days	Every day
Have you been been **trying to cut down on food** to control your weight or shape?	○	○	○	○	○	○	○
Have you **tried not to eat any foods you like** to control your weight and shape?	○	○	○	○	○	○	○
Has **thinking about food or calories made it much harder to concentrate** on things you are interested in, e.g. reading, watching TV, or doing your homework?	○	○	○	○	○	○	○
Have you **felt fat?**	○	○	○	○	○	○	○
Have you had a **very strong wish to lose weight?**	○	○	○	○	○	○	○

Page Break

EDQ-2

Over the ***past two weeks*** / 14 days ...

	Never	A few of the times	Less than half the times	Half the times	More than half the time	Most times	Every time
How often have you **felt guilty after eating** because of the effect on your shape and weight? *(Do not count binges – times when you have eaten a really big amount of food and felt out of control)*	○	○	○	○	○	○	○

EDQ-3

	Not at all<br>0	1	A bit<br>2	3	A lot<br>4	5	Very, very much<br>6
**How unhappy have you felt about your weight?**	○	○	○	○	○	○	○
**How uncomfortable have you felt seeing your body**: for example, in the mirror, in shop windows, when you undress or when you have a bath or shower?	○	○	○	○	○	○	○

Import from library | Add new question

AC 4

AC 4

Which of these colours is the colour of snow?

- Red
- Purple
- Green
- White
- Black

Import from library | Add new question

O2S3

O2S3

Below are listed a number of statements that describe **how you may feel in social situations**.

Please respond to each item how much you **how well it *generally* (most of the time) represents you**. There are no right or wrong answers.

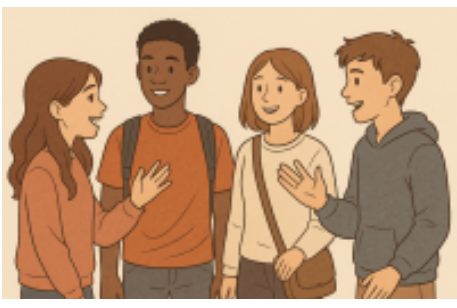

	Strongly Disagree	Moderately Disagree	Moderately Agree	Strongly Agree
1. I worry about the effect I have on other people	◯	◯	◯	◯
2. I delete my social media post if I don't get the responses I wanted	◯	◯	◯	◯
3. I feel that people generally like me	◯	◯	◯	◯
4. I feel anxious before posting anything on social media	◯	◯	◯	◯
5. I always expect criticism	◯	◯	◯	◯
6. I care about what people feel about me	◯	◯	◯	◯
7. Initiating contact online with others, such as sending friend requests, is not something I feel comfortable doing	◯	◯	◯	◯
8. When I update my social media status and no one comments on it, I tend to be disappointed	◯	◯	◯	◯
9. I check responses to my posts on social media very often	◯	◯	◯	◯
10. If others knew the real me, they would not like me	◯	◯	◯	◯
11. My value as a person depends enormously on what others think of me	◯	◯	◯	◯
12. When I update my social media status, I expect others to comment on it	◯	◯	◯	◯
13. I worry about being criticised for things I have said or done	◯	◯	◯	◯
14. I feel others do not understand me	◯	◯	◯	◯
15. I prefer to post on social media sites where I can stay anonymous (i.e. without revealing my name or identity)	◯	◯	◯	◯
16. If other people knew what I am really like, they would think less of me	◯	◯	◯	◯
17. I worry about what others think of me	◯	◯	◯	◯
18. I feel insecure if I don't receive positive responses to my social media posts	◯	◯	◯	◯

Import from library | Add new question

SRT

SRT

In this questionnaire, you will be asked to describe how you ***typically*** **think about social interactions**.

	Not true at all	A little true	Somewhat true	Quite true	Very True
1. The same thoughts about negative social experiences (e.g., argument with a friend) keep going through my mind again and again.	◯	◯	◯	◯	◯
2. Thoughts about positive social interactions pop into my mind, even if I don't want them to.	◯	◯	◯	◯	◯
3. When I feel like a social interaction did not go well, I move on and easily put it out of my mind.	◯	◯	◯	◯	◯
4. Before social interactions I often worry about what others will think of me.	◯	◯	◯	◯	◯
5. Following unpleasant social interactions my thoughts keep repeating themselves.	◯	◯	◯	◯	◯
6. Thoughts about unpleasant social interactions come to my mind without me wanting them to.	◯	◯	◯	◯	◯
7. My thoughts about negative social experiences prevent me from focusing on other things.	◯	◯	◯	◯	◯
8. After positive social interactions I keep going over them in my mind.	◯	◯	◯	◯	◯

Import from library | Add new question

AC 5

AC 5

Great work! You are almost done with the questions!! Just a few more!

For this question, please select the word "carrot" from the list of vegetables below:

- ◯ Cauliflower
- ◯ Lettuce
- ◯ Carrot
- ◯ Pumpkin
- ◯ Broccoli

Import from library | Add new question

Social Comparison

SC - Qs

In the table below, please select the **number** that represents the point that **best describes** **how you see yourself in comparison to others.**

For example, on the following scale from short to tall, if you put a mark at "3" this means you see yourself as shorter than others; if you put a mark at "5" (middle), you see yourself at about average height; and a mark at "7" you see yourself as somewhat taller than others.

	1	2	3	4	5	6	7	8	9	10	
Short	○	○	●	○	●	○	●	○	○	○	Tall
			Shorter than others		Average height		Taller than others				

	1	2	3	4	5	6	7	8	9	10	
Inferior	○	○	○	○	○	○	○	○	○	○	Superior
Incompetent	○	○	○	○	○	○	○	○	○	○	More competent
Unlikeable	○	○	○	○	○	○	○	○	○	○	More likeable
Left out	○	○	○	○	○	○	○	○	○	○	Accepted
Different	○	○	○	○	○	○	○	○	○	○	Same
Untalented	○	○	○	○	○	○	○	○	○	○	More talented
Weaker	○	○	○	○	○	○	○	○	○	○	Stronger
Unconfident	○	○	○	○	○	○	○	○	○	○	More confident
Undesirable	○	○	○	○	○	○	○	○	○	○	More desirable
Unattractive	○	○	○	○	○	○	○	○	○	○	More attractive

Page Break

Appearance

This question is designed to measure what you are **thinking about yourself** **at this moment.** There is no right answer for any statement. Answer these questions as they are **true for you RIGHT NOW**.

	Not At All	A Little Bit	Somewhat	Very Much	Extremely
I feel satisfied with the way my body looks right now	○	○	○	○	○
I feel that others respect and admire me	○	○	○	○	○
I am dissatisfied with my weight	○	○	○	○	○
I feel good about myself	○	○	○	○	○
I am pleased with my appearance right now	○	○	○	○	○
I feel unattractive	○	○	○	○	○

Import from library | Add new question

Physical Activity & Sleep

Sleep

This question is about your **sleep**. Please respond to each question or statement by marking one box per row.

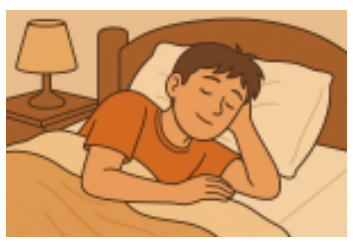

In the ***past 7 days*** ...

	Never	Almost never	Sometimes	Almost always	Always
I had difficulty falling asleep	○	○	○	○	○
I slept through the night	○	○	○	○	○
I had a problem with my sleep	○	○	○	○	○
I had trouble sleeping	○	○	○	○	○
It took me a long time to fall asleep	○	○	○	○	○
I worried about not being able to fall asleep	○	○	○	○	○
I woke up at night and had trouble falling back to sleep	○	○	○	○	○
I tossed and turned at night	○	○	○	○	○

Page Break

PA-1

The next questions are about your levels of physical activity
- i.e., any activity that **increases your heart rate and makes you a bit out of breath**
- e.g., during sport, playing with friends, or walking or cycling places.

Physical activity can be done in
- one block (e.g. 1 hour of sport = 60 minutes) or
- accumulated in multiple blocks over the entire day (e.g. 6 blocks of 10 minutes of, say, walking or cycling = 60 minutes).

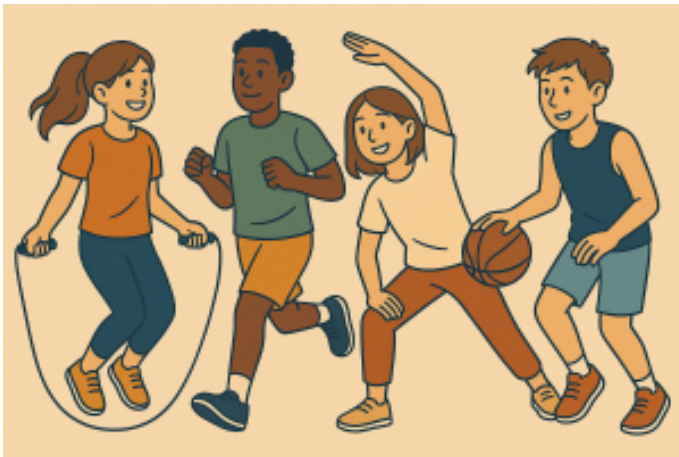

Thinking about this, ***on a typical day***, **how many minutes do you spend** doing this sort of physical activity?

Page Break

PA-2

In the ***past week***, on **how many days** have you done a total of **60min or more of** physical activity like this?

- ○ 1
- ○ 2
- ○ 3
- ○ 4
- ○ 5
- ○ 6
- ○ 7

Import from library   Add new question

Add Block

End of Survey

The final exercise is a short computer-based task. Please be patient while the page loads and during task transitions, which can feel slow.

Please **CLICK HERE** to complete the final task.

Appendix 2. *Parent Part 1 Questionnaire Battery*

Preview Pub

ExpertReview score

Landing Page

Welcome - General | Welcome to the Online Social Interactions and Wellbeing Study

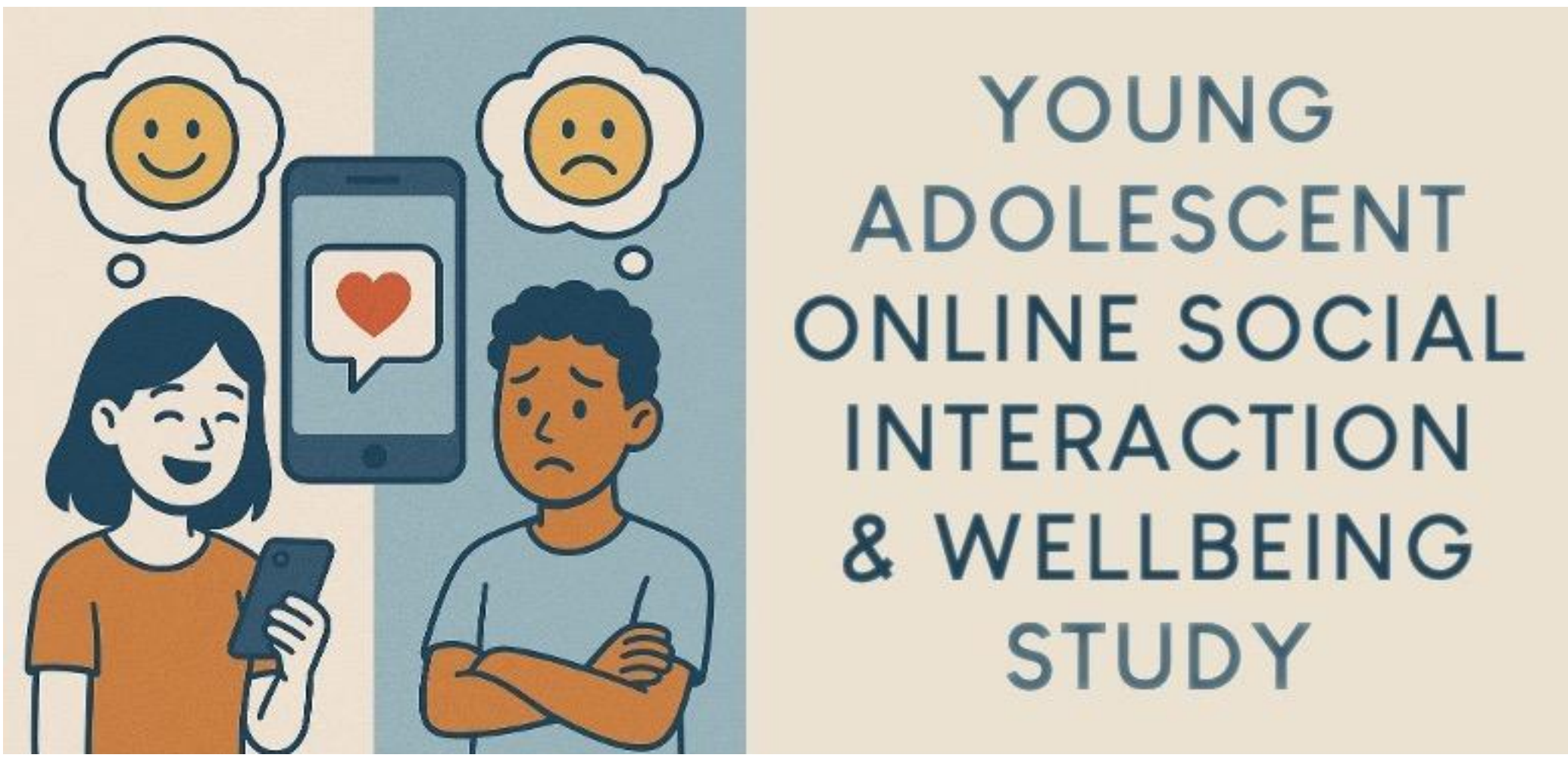

WELCOME BACK!

This survey will ask you some demographic questions as well as questions about your social media and messaging attitudes and behaviours

Import from library | Add new question

Add Block

Email check

Email

What is your email?
(This should auto-fill. If it doesn't please complete with the email you used during consent.)

${e://Field/P_Email}

Import from library | Add new question

Add Block

Demographics

Age

What is **your age** in years?

*

Page Break

Country

What **country** do you and your child live in?

*

- Australia
- UK
- NZ

Page Break

Postcode

*

Display this question

If What country do you and your child live in? Australia Is Selected

What is your **postcode**?

Page Break

Timezone

*

Display this question

If What country do you and your child live in? Australia Is Selected

Which Australian **state or territory** (timezone) are you in?

(*We need this information to ensure we have the right time zone to send your child notifications at times they can respond to them in Part 2*)

- NSW / Victoria / Tasmania / ACT
- QLD
- South Australia
- Western Australia
- Northern Territory

Page Break

Gender

Please select which **gender** you identify with:

- Female
- Male
- Non-binary / third gender
- I use a different term:
- Prefer not to say

Page Break

Ethnicity

Please select which **ethnicity** you identify with:

- Aboriginal or Torres Strait Islander
- Maori
- Black
- Asian
- White
- Hispanic
- Mixed
- Other
- Prefer not to say

Page Break

Education

What is your current / highest level of **education**?

- Primary School
- High School
- Professional / Vocational Training
- University
- Prefer not to say

Page Break

Perceived Wealth

How well off do you think you are?

(*By well off we mean how much money / wealth you have*)

- Not at all
- Not very
- Fairly
- Rather
- Very
- Prefer not to say

Page Break

Q8

Has **your child** ever been **diagnosed with a mental health condition?**

- Yes
- No
- Prefer not to say

Page Break

Q9

Display this question

If Has your child ever been diagnosed with a mental health condition? Yes Is Selected

Please select any mental health conditions your child has ever been diagnosed with:

- Depression
- Anxiety Disorder
- Eating Disorder
- Bipolar disorder (manic depression)
- Schizophrenia
- Post-traumatic stress disorder
- Obsessive compulsive disorder
- Other (please specify)
- Prefer not to say

Page Break

Q10

Display this question

If Has your child ever been diagnosed with a mental health condition? Yes Is Selected

Is **your child** ***currently*** suffering from a mental health condition?

If yes, please select relevant option(s)

- Depression
- Anxiety Disorder
- Eating Disorder
- Bipolar disorder (manic depression)
- Schizophrenia
- Post-traumatic stress disorder
- Obsessive compulsive disorder
- Other (please specify)
- No
- Prefer not to say

Page Break

ND Type

Has **<u>your child</u>** received a **neurological or neurodevelopmental related diagnosis?**

If yes, select relevant option(s)

- ☐ Epilepsy
- ☐ Seizures
- ☐ ADHD
- ☐ Autism / Autism Spectrum
- ☐ Dyslexia (Reading challenges)
- ☐ Dysgraphia (Writing challenges)
- ☐ Discalculia (Calculation challenges)
- ☐ Other (please specify) ______
- ☐ No
- ☐ Prefer not to say

Import from library | Add new question

Add Block

AC 1

AC 1 *

For this question, please select the word "broccoli" from the list of vegetables below:

- ○ Pumpkin
- ○ Lettuce
- ○ Carrot
- ○ Broccoli
- ○ Cauliflower

Import from library | Add new question

Add Block

Views on child's SM & Internet Use

Intro to OSI

Some of the following questions refer to social media and messaging. Examples of these include:

Social media
e.g. Facebook, Instagram, X, TikTok, Snapchat

and

Messaging
e.g. Messages (Text), WhatsApp, Facebook / Kids Messenger, Telegram, Discord, WeChat

For the purpose of this study, **You Tube and other internet and gaming sites are not considered social media or messaging.**

Page Break

Know child's use?

Thinking about **your child's use of the internet and social media** ...

How much do you think you know about what your child does on the internet?

- Nothing
- Just a little
- Quite a bit
- A lot
- I don't know
- Prefer not to say

Page Break

Permissions

Do you allow your child to do the following things on the internet and if so, do they need your permission to do them?

<u>My child</u> is allowed to ...

	Anytime	Only with permission or supervision	Never	I don't know	Prefer not to say
Use a web or phone camera (e.g., for Facetime / other video chat)	○	○	○	○	○
Download music or films	○	○	○	○	○
Use a social networking site (e.g. Facebook, Instagram, X, TikTok, Snapchat)	○	○	○	○	○
Watch video clips (e.g., on YouTube)	○	○	○	○	○
Play games with other people online	○	○	○	○	○
Read / watch news online	○	○	○	○	○
Use the internet for school / work	○	○	○	○	○
Spend time in a virtual world (e.g. Minecraft)	○	○	○	○	○
Share photos or videos of <u>*themselves*</u> online	○	○	○	○	○
Share photos, videos of music <u>*of others*</u> online	○	○	○	○	○

Page Break

Checking

Display this question

If Do you allow your child to do the following things on the internet and if so, do they need your p... - Never Is Not Equal to 9

When <u>your child</u> uses the internet, **how often do you check** the following things afterwards?

	Never	Hardly ever	Some times	Often	Very often	Not Applic-able	Prefer not to say
Which friends or contacts they added to their social networking profile	○	○	○	○	○	○	○
The messages in their email or apps for communicating with people	○	○	○	○	○	○	○
Their profile on a social networking site or online group	○	○	○	○	○	○	○
Which online content they viewed	○	○	○	○	○	○	○
The apps they downloaded	○	○	○	○	○	○	○
The in-app purchases they made	○	○	○	○	○	○	○

Page Break

SM use ever

Has **<u>your child</u> ever used social media?** (e.g., Facebook, Instagram, X, TikTok, Snapchat)

- ○ Yes
- ○ No
- ○ Don't know
- ○ Prefer not to say

Page Break

SM - Age first use

Display this question

If Has your child ever used social media? (e.g., Facebook, Instagram, X, TikTok, Snapchat) Yes Is Selected

What **age** was your child when they **first used social media?**

15

Page Break

TX ever

Has **your child ever used** a **messaging** application? (e.g. Messages / Text, WhatsApp, Facebook Messenger, Kids Messenger, Telegram, Discord, WeChat)

- Yes
- No
- I don't know
- Prefer not to say

Page Break

TX - Age first use

Display this question

If Has your child ever used a messaging application? (e.g. Messages / Text, WhatsApp, Facebook Messe... Yes Is Selected

What **age** was your child when they **first used** a **messaging** application?

15

Page Break

Own account

Does **your child** have their **own profile / account that they currently use** on a social media, messaging or gaming application or site?

	Yes	No	I don't know	Prefer not to say
**Social Media** (e.g., Facebook, Instagram, X, TikTok, Snapchat)	○	○	○	○
**Messaging** (e.g. Messages, WhatsApp, Facebook/Kids Messenger, Telegram, Discord, WeChat)?	○	○	○	
**Gaming site** (Fortnight, Roblox)?	○	○	○	○

Page Break

Own phone

Does **<u>your child</u>** have their **own phone**?

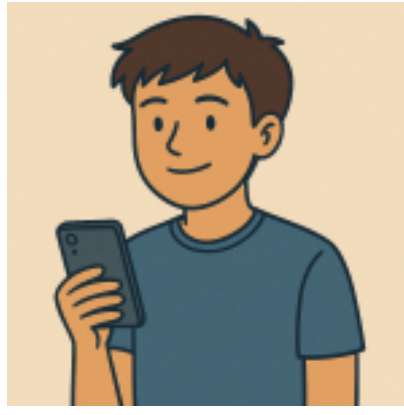

Yes

No

Page Break

SM on phone

Display this question

If Does your child have their own phone? Yes Is Selected

Does **<u>your child</u>** have access to **social media** **on their phone**?

Yes

No

I don't know

Import from library

Add new question

Add Block

Social Media Mindset - Child

SM Mindset

For this questionnaire, we want you to think about **how you think about __your child's__ social media use.**

Please rate the __extent to which you agree__ with each of the following statements.

	Strongly Disagree	Disagree	Neither Agree nor Disagree	Agree	Strongly agree	Not Applic-able
Using social media is meaningful for my child	○	○	○	○	○	○
Using social media is a waste of time for my child	○	○	○	○	○	○
Using social media is fun and enjoyable for my child	○	○	○	○	○	○
Using social media strengthens and supports my child's relationships	○	○	○	○	○	○
Social media is a helpful tool that my child uses	○	○	○	○	○	○
My child is good at managing the ways they use social media	○	○	○	○	○	○
My child is in control of how they use social media	○	○	○	○	○	○
My child gets distracted by social media	○	○	○	○	○	○
My child finds it hard to resist the pull of social media	○	○	○	○	○	○
My child is often manipulated by social media	○	○	○	○	○	○

Import from library | Add new question

Add Block

AC2

AC2

Which of these things does not move?

- ○ Car
- ○ Plane
- ○ Tram
- ○ Statue
- ○ Bike

Import from library | Add new question

Add Block

Digital Parenting Scale

Digital parenting

Thinking about **your experiences with your child** and **digital devices**, how frequently are the following statements true?

	Never	Occasion-ally	Sometimes	Often	Always
I have rules about the amount of time my child is allowed to spend on digital devices	○	○	○	○	○
I have rules about when my child is allowed to access digital devices	○	○	○	○	○
I promote offline activities to ensure that my child does not overuse their digital devices	○	○	○	○	○
I prevent my child from using their digital devices too much by engaging them in other activities	○	○	○	○	○
I try to strike a balance between my child's digital device use and other offline activities	○	○	○	○	○
I have rules about what my child is allowed to do on digital devices	○	○	○	○	○
I limit my own digital device use to be a role model for my child	○	○	○	○	○
I check what my child is sharing on social media and my child knows about this.	○	○	○	○	○
I find it difficult to parent my child's digital device use when their school requires use of digital devices.	○	○	○	○	○
I find it difficult balancing my child's use of digital devices for school with how I want to regulate their digital device use.	○	○	○	○	○
I find it difficult differentiating between when my child is using digital devices for school or for fun	○	○	○	○	○
There are tensions between me and my child if I try to regulate their digital device use	○	○	○	○	○
My child and I are in conflict about their digital device use	○	○	○	○	○
My child tries to dodge my rules by hiding their digital device use (e.g. by borrowing another device or deleting browser history)	○	○	○	○	○
I join my child in activities on their digital devices	○	○	○	○	○
My child and I do activities on their digital devices together	○	○	○	○	○
I offer to actively participate in what my child is doing on their digital devices	○	○	○	○	○
I encourage my child to be a good citizen online	○	○	○	○	○
I discuss with my child about appropriate digital behaviors	○	○	○	○	○
When needed, I help my child when they have a negative experience while using digital devices	○	○	○	○	○
I teach my child how to behave on digital devices	○	○	○	○	○
I teach my child how to stay safe when using their digital devices	○	○	○	○	○
I teach my child about some of the dangers that they could encounter while using digital devices	○	○	○	○	○
When needed, I help my child when they encounter something distressing while using digital devices	○	○	○	○	○
I am a trusted point of call if my child experiences something negative while using digital devices	○	○	○	○	○
I believe that knowing how to use digital devices will help my child in the future	○	○	○	○	○
I rely on my family or friends to help me monitor my child's digital device use	○	○	○	○	○
I rely on my family or friends to alert me if there are problems with my child's online activities	○	○	○	○	○
I rely on my family or friends to help me regulate my child's digital device use	○	○	○	○	○
I monitor my child's digital device use through the help of technologies (e.g. monitoring apps) and my child knows about this	○	○	○	○	○
I use technological aids (e.g. apps) to monitor what my child is doing on digital devices	○	○	○	○	○
I use technology to restrict what my child can do on digital devices (e.g. through filtering, blocking, or locking down features)	○	○	○	○	○

Import from library

Add new question

Add Block

Parents' Own SM Use and Behaviours

SM - ever used

Have **you** **ever used** **social media?** (e.g., Facebook, Instagram, X, TikTok, Snapchat)

- Yes
- No
- Prefer not to say

Page Break

SM - # used

Display this question

If Have you ever used social media? (e.g., Facebook, Instagram, X, TikTok, Snapchat) Yes Is Selected

How many different **social media** do **you** **use once a week or more?**

(e.g. Snapchat only = 1, Snapchat & Instagram = 2, etc)

0

Page Break

Own accounts

Do you **currently use** **your** **own account / profile** for ...

	Yes	No	Prefer not to say
**Social Media** (e.g., Facebook, Instagram, X, TikTok, Snapchat)?	○	○	○
**Messaging** (e.g. Messages, WhatsApp, Facebook Messenger, Telegram, Discord, WeChat)?	○	○	○
**Gaming** (e.g., Fortnight, Roblox)?	○	○	○

Page Break

SM Use type

Display this question

If Do you currently use your own account / profile for ... Social Media
 (e.g., Facebook, Instagram, X, TikTok, Snapchat)? - Yes Is Selected

When using **social media**, **do** **you** **spend more time** ...

- Browsing
- Sharing, posting or liking
- Time spent equally
- Prefer not to say

Import from library

Add new question

Add Block

AC3

AC3

What is the capital of France?

- Sydney
- Paris
- Melbourne
- Moscow
- Tokyo

Import from library | Add new question

Add Block

Social Media Mindset - Self

SM Mindset

For this question, we want you to think about the **ways that** **you** **think about and personally use** social media.

Please rate the extent to which you agree with each statement.

	Strongly Disagree	Disagree	Neither Agree nor Disagree	Agree	Strongly agree	Not Applic-able
Using social media is meaningful for me	○	○	○	○	○	○
Using social media is a waste of time for me	○	○	○	○	○	○
Using social media is fun and enjoyable for me	○	○	○	○	○	○
Using social media strengthens and supports my relationships	○	○	○	○	○	○
Social media is a helpful tool that I use	○	○	○	○	○	○
I'm good at managing the ways I use social media	○	○	○	○	○	○
I'm in control of how I use social media	○	○	○	○	○	○
I end up using social media even when I don't mean to	○	○	○	○	○	○
I find it hard to resist the pull of social media	○	○	○	○	○	○
I am often manipulated by social media	○	○	○	○	○	○

Import from library | Add new question

Add Block

Aus Social Media Ban

Aus Ban - aware

Before this question, did you know that **the Government is banning social media accounts for people under 16 years of age?**

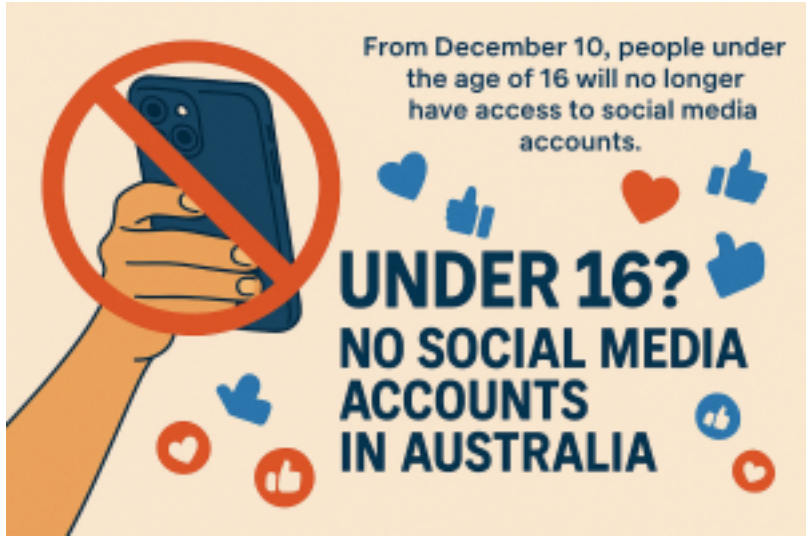

- Yes
- No
- Prefer not to say

Page Break

Restrictions - early

Display this question

If Before this question, did you know that the Government is banning social media accounts for peopl... Yes Is Selected

Have you **increased restrictions on your child's access to social media already** because of this (before the ban is in place)?

- Yes
- No
- Prefer not to say

Page Break

Restrictions - how

Display this question

If Have you increased restrictions on your child's access to social media already because of this (b... Yes Is Selected

In what way(s) have you increased restrictions on your child's access to social media?

- My child had one or more social media account(s), but they are no longer allowed to use social media at all
- My child had one or more social media account(s), but they are no longer allowed an account(s). However, they are allowed to view social media that is accessible without an account
- My child has have never had a social media account, and now they are no longer allowed to use social media at all
- Other (please specify)
- Prefer not to say

Page Break

Early change own use

Display this question

If Before this question, did you know that the Government is banning social media accounts for peopl... Yes Is Selected

Have you changed your own social media use since knowing about the ban for under 16-year-olds?

- Yes
- No
- Don't know
- Prefer not to say

Page Break

Change own use - how

Display this question

If Have you changed your own social media use since knowing about the ban for under 16-year-olds? Yes Is Selected

In what way have you changed your own social media use since knowing this?

- I no longer use social media
- I deleted my social media account but still browse publicly available social media
- I still use social media but not in front of my child(ren)
- Other (please specify)
- Prefer not to say

Page Break

After change use

Will you **change** your social media ***once the ban is introduced*?**

- Yes
- No
- Don't know
- Prefer not to say

Page Break

After change how

Display this question

If Will you change your social media once the ban is introduced? Yes Is Selected

How will your social media use change once the ban is introduced?

- I will stop using social media
- I will delete my accounts but will browse publicly available social media
- I won't use social media in front of my child(ren)
- Other (please specify)
- I don't know
- Prefer not to say

Import from library

Add new question

Add Block

End of Survey

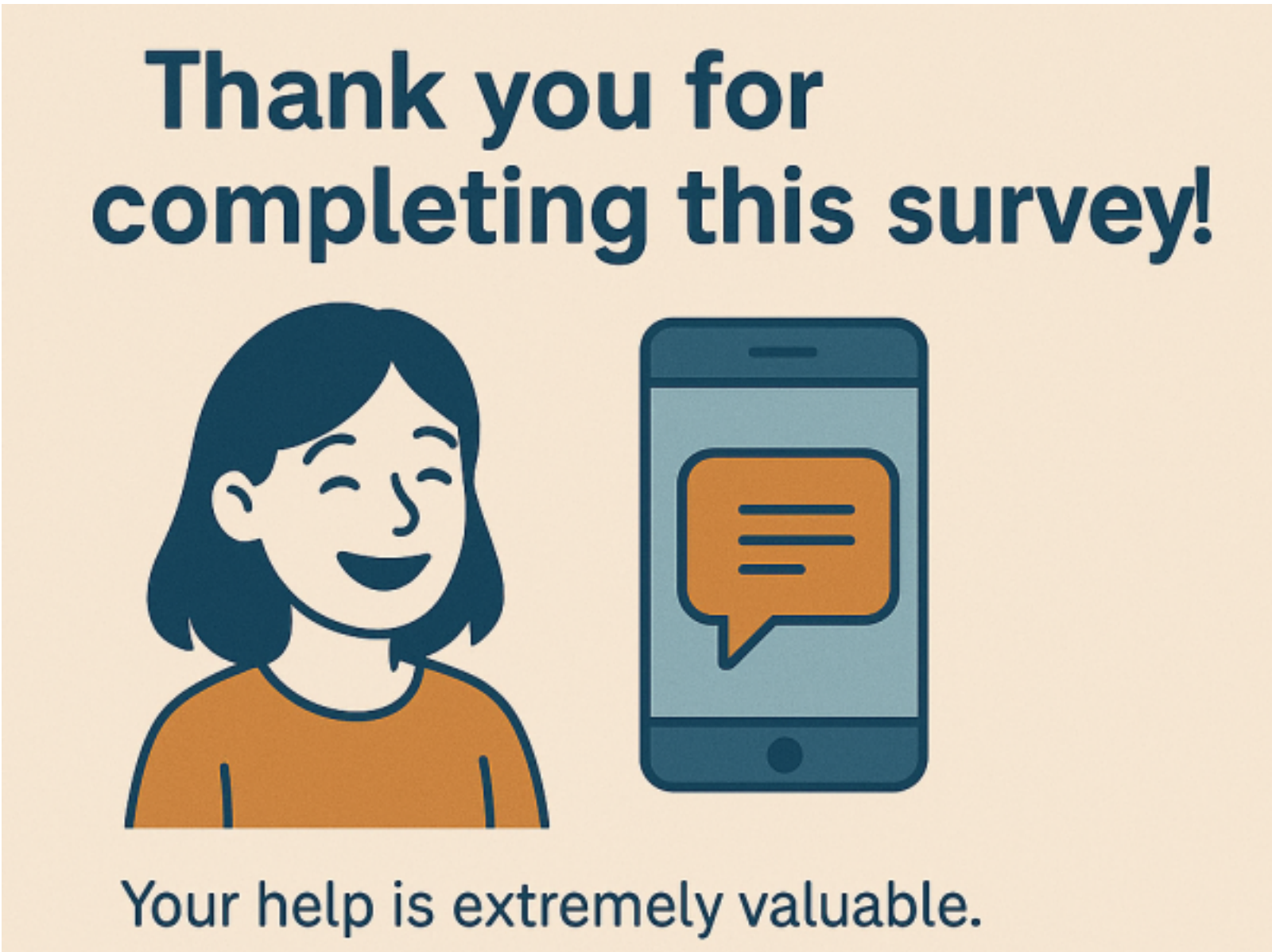

We will be in touch with you prior to the next round of questions in about 6-months' time.

Appendix 3. *Adolescent EMA Survey*

1.2 - OSIYAW - Adolescent EMA

ExpertReview score Fair

Landing page & ID

Prolific Welcome

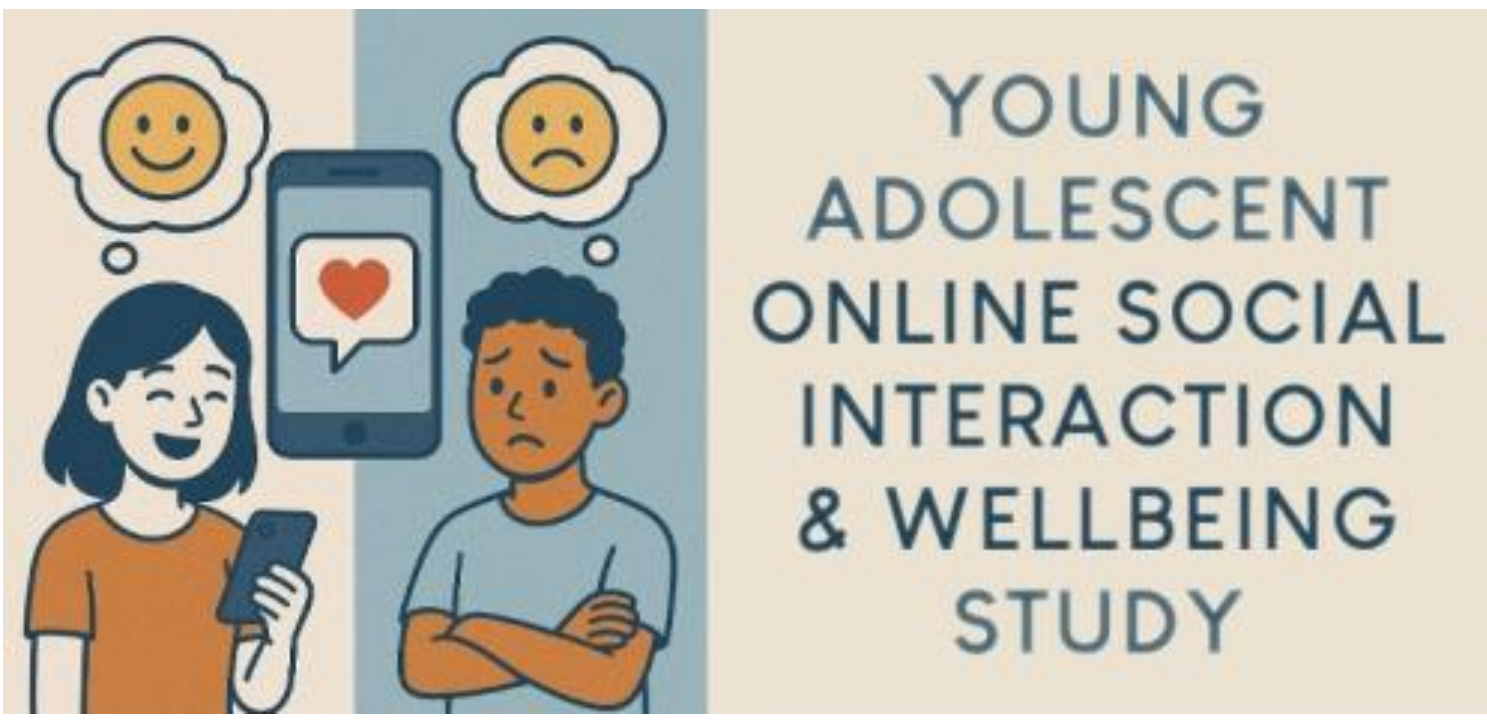

WELCOME BACK!!

Details

What is your **phone number**?

This response should auto-fill with the correct phone number (Don't worry if the first zero is missing). If not, please ensure this is filled in!!

${e://Field/A_Ph}

EMA#

Display this question

If False

EMA#

${e://Field/ema}

Import from library

Add new question

Add Block

Current State

Affective State *

How do you **feel *right now***?

Very Unhappy **Very Happy**

0 100

Anxiety *

Anxious **Relaxed**

0 100

Body Image *

Uncomfortable with my body **Comfortable with my body**

0 100

Page Break

Likeability *

How do you feel **relative to others** ***right now***?

Unlikeable **Likeable**

0 100

Affiliation

	Left Out	Included
	0	100

Import from library | Add new question

Add Block

Online Social Interaction

Online - passive

In the last two hours (120 minutes), ***how many minutes*** did you spend **passively reading or viewing** online posts or messages from ...?

	0	120
**People you know**		
AND / OR **Strangers (e.g., influencers)**		

Page Break

Online - connection

In the last two hours, **have you interacted socially with others ONLINE** (via video call, messaging, social media, in-game or other online chat)?

- Yes
- No

Page Break

Online - Mode

Display this question

If In the last two hours, have you interacted socially with others ONLINE (via video call, messaging... Yes Is Selected

Now, please think about the single ***most important*** **ONLINE social interaction** you had in the last two hours, was it via....

- **Video call** (e.g. FaceTime)
- **Messaging** (e.g. SMS, Discord, WhatsApp, Facebook Messenger, etc)
- **Social media** (e.g.Instagram, TikTok, Facebook, Snapchat, etc.)
- **Gaming** (e.g., Fortnite. Roblox)
- **Other (please specify):** ________

Page Break

Online - # people

Display this question

If In the last two hours, have you interacted socially with others ONLINE (via video call, messaging... Yes Is Selected

For this same, most important **ONLINE** social interaction, **who and how many people interacted?**

- Me and one friend or family member
- Me and one stranger
- Private Group with friends and/or family only
- Private Group including strangers / people I didn't know
- Public Group (i.e., anyone could participate / view) ________

Page Break

Online - Affective

Display this question

If In the last two hours, have you interacted socially with others ONLINE (via video call, messaging... Yes Is Selected

For this same **ONLINE** interaction, **how did it make you feel overall?**

Very Unhappy **Very Happy**

0 100

Page Break

Online- Affiliative

Display this question

If In the last two hours, have you interacted socially with others ONLINE (via video call, messaging... Yes Is Selected

For this same **ONLINE** interaction, **how did it make you feel socially?**

Left out / Disliked / Misunderstood **Included / Liked / Understood**

0 100

Import from library Add new question

Add Block

Social Interation - F2F

F2F - any

Have you **interacted socially with others IN-PERSON** (face-to-face) in the last two hours?

- Yes
- No

Page Break

F2F - how many?

Display this question

If Have you interacted socially with others IN-PERSON (face-to-face) in the last two hours? Yes Is Selected

Now, please think about the single, most important **IN-PERSON** **social interaction** you had in the last two hours.

Who and how many people interacted?

- Me and one friend or family member
- Me and one stranger
- Private Group with friends and/or family only
- Private Group including strangers / people I didn't know
- Public Group (i.e., anyone could participate)

Page Break

F2F - Affective

Display this question

If Have you interacted socially with others IN-PERSON (face-to-face) in the last two hours? Yes Is Selected

For this same **IN-PERSON** interaction, **how did it make you feel overall?**

	**Very Unhappy**	**Very Happy**
	0	100

Page Break

F2F - Affiliative

Display this question

If Have you interacted socially with others IN-PERSON (face-to-face) in the last two hours? Yes Is Selected

For this same **IN-PERSON** interaction, **how did it make you feel socially?**

	**Left out / Disliked / Misunderstood**	**Included / Liked / Understood**
	0	100

Import from library

Add new question

Add Block

Anxiety re Affordances

anx_miss

*

In the last two hours, have you **worried about** the following ...

... **Missing** **messages or social media** posts/updates from others?

- Not at all
- Once or twice
- A few times
- A lot
- Constantly

Q28

*

... **Responding fast enough** to **messages or social media** posts/updates from others?

- Not at all
- Once or twice
- A few times
- A lot
- Constantly

Q29

*

... **Other people not responding** to a **message or social media** update you have sent/posted?

- Not at all
- Once or twice
- A few times
- A lot
- Constantly

Q30

... Other people misunderstanding a **message or social media** update that you sent/posted?

- Not at all
- Once or twice
- A few times
- A lot
- Constantly

Import from library

Add new question

Add Block

Social Comparison

Compare?

In the past two-hours, have you **compared your appearance** to another individual?

- Yes
- No
- Prefer not to say

Page Break

Compare - context

Display this question

If In the past two-hours, have you compared your appearance to another individual? Yes Is Selected

In what context did you compare your appearance to another person?

- Social media (e.g., Instagram, TikTok, Facebook, Snapchat, LinkedIn)
- Magazine
- TV / Movie
- Advertisement
- In-person
- Other (please specify)
- Prefer not to say

Page Break

Compare - result

Display this question

If In the past two-hours, have you compared your appearance to another individual? Yes Is Selected

How did you think you looked compared to the other person?

- Much better
- Somewhat better
- About the same
- Somewhat worse
- Much worse

Import from library | Add new question

Add Block

Harmful Content

Harmful - warning

The following questions refer to unpleasant and / or unwanted experiences that may be confronting.

If you do not wish to answer a question or complete a task, you may skip it and go to the next question or stop immediately.

Page Break

Harmful - exposure

In the past two hours, have you experienced a situation **ONLINE** where **someone treated you in a hurtful or nasty way?**

- On Social Media
- Via Messaging
- Elsewhere online
- No, none of these
- Prefer not to say

Page Break

Q31

In the past two hours have you experienced a situation **ONLINE** where you **saw images you did not want to see?**

(e.g., sexual images, ways to be very thin, hate messages attacking groups or individuals, referencing drinking alcohol or taking illegal drugs, gory or violent images)

- On Social Media
- Via Messaging
- Elsewhere online
- No, none of these
- Prefer not to say

Import from library
Add new question

Add Block

End of Survey

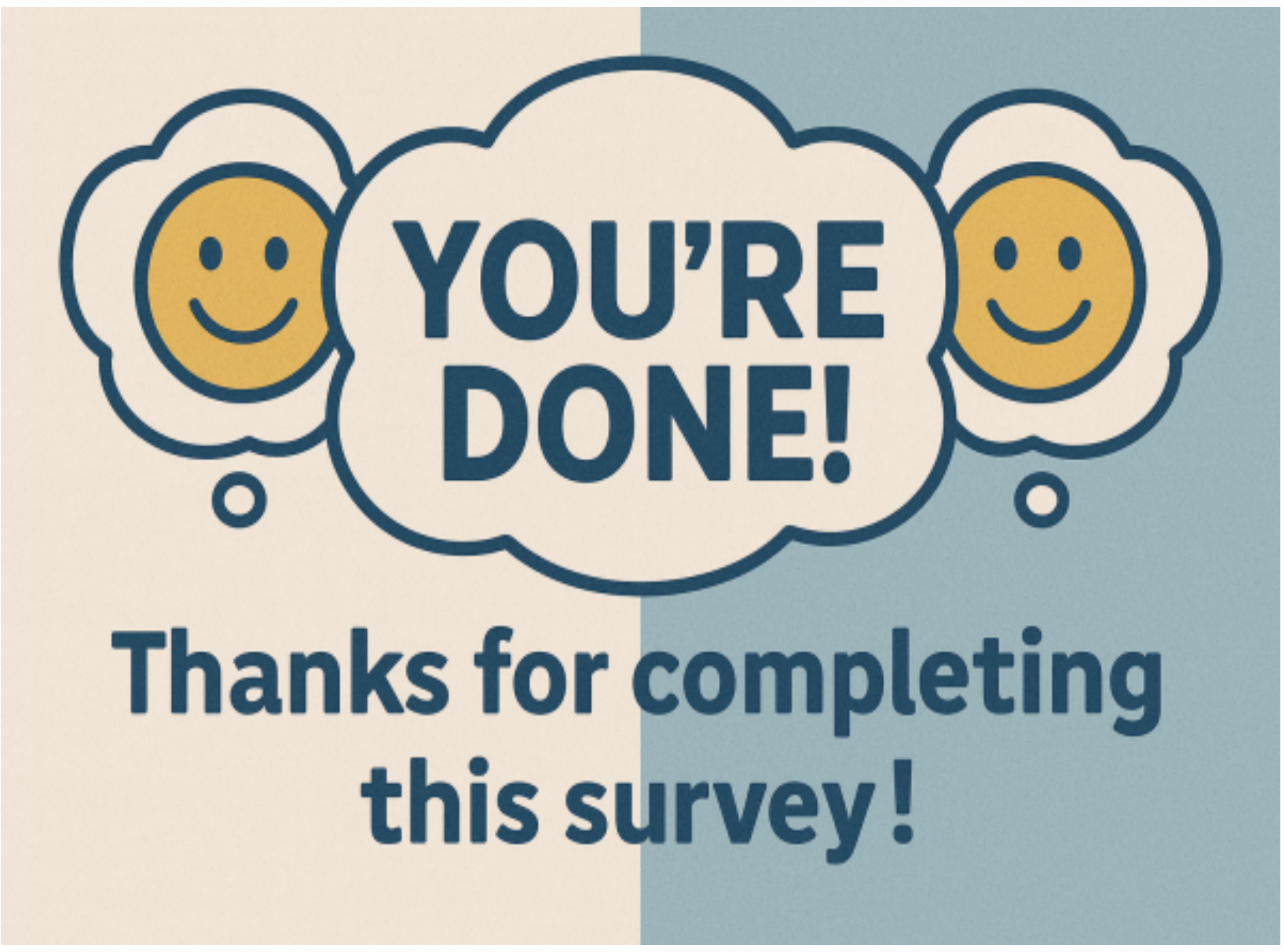

If these questions were distressing or raised issues for you, please discuss it with a trusted adult and / or click on this link to access a list of support services: Support Services - Australian Adolescent.docx .

Figure S2. *Infographic Shared with Participants After Baseline*

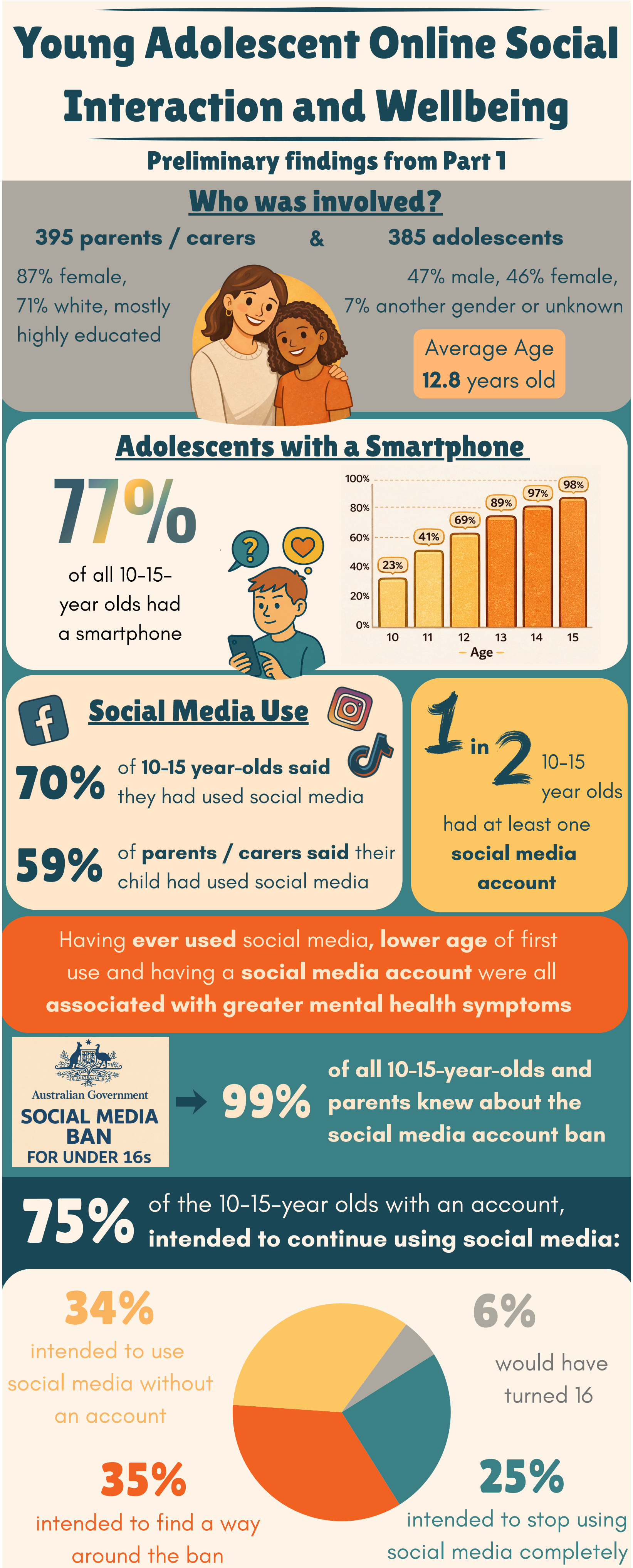

Supplement: Supplementary file 1 [file bmjopen-16-8-s001.pdf]
