# Supplementary material for: Tracking mental health and cognitive changes following the Australian social media minimum age legislation: a 4-wave longitudinal study protocol
Source: BMJ Open. 2026 Aug 5;16(8):e120216. doi: 10.1136/bmjopen-2026-120216 (PMC13448575; doi:10.1136/bmjopen-2026-120216)
Supplement: Supplementary file 2 [file bmjopen-2026-120216.reviewer_comments.pdf]

PEER REVIEW HISTORY

BMJ Open publishes all reviews undertaken for accepted manuscripts. Reviewers are asked to complete a checklist review form and are provided with free text boxes to elaborate on their assessment. These free text comments are reproduced below.

ARTICLE DETAILS

Title (Provisional)

TRACKING MENTAL HEALTH AND COGNITIVE CHANGES FOLLOWING THE AUSTRALIAN SOCIAL MEDIA MINIMUM AGE LEGISLATION: A 4-WAVE LONGITUDINAL STUDY PROTOCOL

Authors

Bilston, Anna; Brown, Lyndsay; Qi, Xu; Morgan, Emily; Hasan, Yasmin; Stevens, Sarah; Wang, Weike; Werner-Seidler, Aliza; Schweizer, Susanne

---

VERSION 1 - REVIEW

**Reviewer**	**1**
**Name**	**Iveson, Matthew**
**Affiliation**	**University of Edinburgh, Department of Psychology**
**Date**	**08-May-2026**
**COI**	**None**

Australian adolescent social media use and mental health before and after the social media minium age legislation: a study protocol.

This manuscript details the protocol for an ongoing study of social media use and mental health around the ban of social media for adolescents in Australia. The study is interesting, and I agree that the opportunity to examine causal associations with social media use is not to be missed. The protocol is well thought out, and the study design seems strong. However, there are a few issues regarding the details provided in the protocol which I believe should be addressed before publication. Firstly, I believe the rationale for examining specific factors should be clear and prior work explained to as to position the study effectively. I would also expect much of the study documentation, especially draft surveys, to be included as supplementary material. Finally, a topical study such as this should be extremely clear about public involvement and engagement activities and how (if) stakeholders have contributed to the study design. I have provided detailed comments about these and other minor issues below:

Title

Note the typo in ‘minimum’

Abstract

While the abstract effectively communicates the aim for a mixed methods approach, it isn’t clear how different modalities of data will be integrated, or what questions the linear regression and dynamic SEM is designed to answer.

Ethics and dissemination

Given the nature of the topic I would expect to see substantial stakeholder involvement and engagement, particularly with young people themselves. If engagement has been done already or is planned, this should be mentioned here and throughout the protocol.

Strengths and limitations

I wonder how EMA is designed to work during normal hours, as young people will likely be in school where mobile phones are banned in most states.

In my eyes, the biggest strength is the natural experiment itself and the opportunity to assess causal associations, but this isn’t mentioned.

Introduction

‘The study’ (Page 6, paragraph 3) hasn’t been introduced yet, as the authors are still outlining the problem.

It should be clearer what the authors mean by “parental attitudes” etc. and what previous literature has shown. Similarly with “societal rules and norms”

Study design

I would expect to see the design chosen with reference to previous work, particularly when it comes to the measures being used. For example, what mental health measures/questionnaires are being used? Why has the backwards span task been chosen (especially over other tasks)? I note also that the backwards span task mentioned in the Study Design section (Page 7, paragraph 3) are not mentioned in any detail in the Measurements section later, just a reference to previous uses.

Participant Sample Size and Recruitment

Having a smartphone was also an inclusion criteria for the screenshots of usage, presumably.

Any inclusion/exclusion criteria for the parents/carers should also be acknowledged.

It isn’t clear whether there are plans to recontact/re-recruit parents or adolescents that started the baseline survey without a matching partner.

It’s great that the authors have already highlighted the representativeness (or lack of) for the baseline sample. It would be good to see what the plans are to account for this in the analyses (e.g., constructing weights).

From table 1 it appears that most adolescents are relatively young. It may be good to also present number in age bands, as I'd imagine those aged around 15 will be of particular interest since they will span the boundary of the ban.

Table 2 includes the age of parents/carers, but still labels them as 'all 10-15 years' (Page 19)

Patient and Public Involvement

As noted in the Ethics section, I would expect a study as topical as this to demonstrate some direct engagement with relevant stakeholders. It would make sense, for example, to give young people some say on what questions are included in the survey and EMA components, given that they are experts by experience. Currently, it sounds like the study relies on precedent and briefings, which doesn't seem sufficient.

Measurements

As noted in the Study design section, what are the specifics of the backwards span task? What are the stimuli, what are the conditions, how many trials, how long is the task, is there feedback, etc.

I appreciate the tables summarising the survey questions, but note that most readers would expect the full survey and EMA to be accessible (including logic) in the supplementary/appendix (e.g., as a PDF).

While they are summarised in the tables, there is no detail about the mental health and wellbeing measures in the actual text. There should at least be mention of specific scales and the intended aim/point of each (e.g., measuring psychological distress).

Data analysis

Again, I would encourage the authors to think about producing sample weights, given the representativeness issue but also likely drop-out longitudinally. This would benefit any reuse of the cohort in future, too.

It would be good to see a little more detail on the inferential analysis, or at least an example. What is the most important/basic model for assessing the interrupted time series? Is the SEM part intended to help boil-down the various questionnaires and scales into something more latent, if so what? Similarly, it would be good to identify any confounders (e.g., parental education, source of recruitment) that would be important to account for in analyses.

Ethics and safety considerations

I would like to see some mention of the safety of the data, too, and the need to minimise the risk of identification. How will the authors ensure that this very personal data cannot be used to reidentify potentially vulnerable adolescents?

Dissemination of findings

I would also expect some plan for data accessibility. Will the data be accessible to other researchers, if so will it be deidentified (has it been consented), where will it be deposited, etc.?

VERSION 1 - AUTHOR RESPONSE

Reviewer: 1
Dr. Matthew Iveson, University of Edinburgh, Administrative Data Research Centre - Scotland
Comments to the Author:
Australian adolescent social media use and mental health before and after the social media minium age legislation: a study protocol.

This manuscript details the protocol for an ongoing study of social media use and mental health around the ban of social media for adolescents in Australia. The study is interesting, and I agree that the opportunity to examine causal associations with social media use is not to be missed. The protocol is well thought out, and the study design seems strong. However, there are a few issues regarding the details provided in the protocol which I believe should be addressed before publication.

Firstly, I believe the rationale for examining specific factors should be clear and prior work explained to as to position the study effectively.

I would also expect much of the study documentation, especially draft surveys, to be included as supplementary material.

Finally, a topical study such as this should be extremely clear about public involvement and engagement activities and how (if) stakeholders have contributed to the study design. I have provided detailed comments about these and other minor issues below:

(6) Title
Note the typo in 'minimum'

Thank you. This typo has been rectified.

(7) Abstract
While the abstract effectively communicates the aim for a mixed methods approach, it isn't clear how different modalities of data will be integrated, or what questions the linear regression and dynamic SEM is designed to answer.

To clarify how we will integrate data modalities, and after a review of expected sample size, we have revised the ***Abstract / Methods and analysis*** section as follows:

Linear regression analyses will be used to assess correlational relationships between social media experiences and responses and mental health outcomes, as well as how individual factors moderate these relationships. To assess these relationships over time, and between momentary experiences and responses (EMA data) and more stable trait-based (individual) and environmental (parenting and government restrictions) factors we will use linear mixed-effects models and mixed-effects segmented regression.

(8) Ethics and dissemination
Given the nature of the topic I would expect to see substantial stakeholder involvement and engagement, particularly with young people themselves. If engagement has been done already or is planned, this should be mentioned here and throughout the protocol.

We describe our previous stakeholder engagement in response to comment 18 below. We have also revised the following sections to note how we disseminate findings throughout the study:

Abstract:

This study has been approved by the UNSW Human Research Ethics Committee (iRECS 9197). Findings from this study will help identify potential opportunities for interventions at an individual, micro (parental) and macro (governmental) level, to mitigate any negative impacts of social media for younger adolescents. Results will be disseminated through peer-reviewed publications, conference presentations, public engagement opportunities and researcher associated websites. After each assessment timepoint core findings will also be distilled into engaging formats such as infographics and videos, which will be shared with young people and their parent/carer.

Ethics and dissemination/Dissemination of findings:

The results of this study will be disseminated through multiple channels including academic journals, conference presentations, and researcher related websites. Participant confidentiality will be maintained by reporting only aggregate results and not including any individually identifying information in publications. Following each assessment timepoint core findings will also be captured in engaging format such as infographics and short videos. These materials will be shared with young people and their carers (e.g., following baseline descriptives were captured in an infographic; Figure S2). Participants who would like to receive the study the full results will be able to provide their email or postal address to the research team who will share aggregated findings only. Additionally, included participants have provided consent to de-identified information being shared for use in future research in an open access repository that will also include all analysis and measures used in the study: https://osf.io/thky7/overview.

(9) Strengths and limitations

I wonder how EMA is designed to work during normal hours, as young people will likely be in school where mobile phones are banned in most states.

This is an important consideration, which we addressed in the study design by only sampling after school hours. This has been detailed in the revised ***Method/Measurements/EMA*** section:

Participants with smartphones will also complete EMA surveys as an ecologically valid method of assessing the relative effects of adolescents' online and offline social interactions. The surveys were designed to be completed on participants' phones in 1-2 minutes (approximately 30 minutes total per survey period). EMA prompts will be sent outside of school hours, with the first prompt at 3:40pm to enable us to capture interactions that have happened in the final hours of school, then at 5:40pm and 8:00pm. The EMA period will run for 5-days from Wednesday to Sunday capturing both weekday and weekend interactions.

While this will capture interactions at the time of day when 10-15-year-old Australians have the most time to interact socially both online and in-person, these interactions won't capture interactions before and throughout the school day, which will be by their nature largely in-person, given a lack of permitted access to mobile phones. We now note this limitation in the ***Strengths and limitations of this study*** section as follows:

- *ecological momentary assessments are limited by not capturing interactions before and during the school day when participants are not permitted access to their mobile phones;*

(10) In my eyes, the biggest strength is the natural experiment itself and the opportunity to assess causal associations, but this isn't mentioned.

The ***Strengths and limitations*** section has been edited as follows:

- *ecological momentary assessments to capture mood, mental health symptoms and online exposures in close-to-real time, minimising recall bias, combined with a longitudinal design to capture changes both within and between participants across a five-year age range, enabling investigation of the causal role of social media exposure on mental health by age;*

- *matched parent/carer and adolescent dyads to assess associations with digital parenting as well as data collection before and ~6-, 12- and 18-months after the introduction of the Social Media Minimum Age legislation, enabling examination of the potential impacts of the Australian Government policy; thereby assessing two levels of external influence on adolescent social media behaviours.*

(11) Introduction
'The study' (Page 6, paragraph 3) hasn't been introduced yet, as the authors are still outlining the problem. It should be clearer what the authors mean by "parental attitudes" etc. and what previous literature has shown. Similarly, with "societal rules and norms"

To be consistent and clearer regarding the study context, this section on the ***Introduction*** (paragraph 3) has been edited to now read as follows:

At a micro level, adolescents' online exposure and experiences are likely to be influenced by parental positive or negative views of social media, their attitude to the acceptable amount and type of social media exposure for their children and themselves, and the way they negotiate social media access with their children, often referred to as "digital parenting" [3–6]. Digital parenting styles may also impact mental health outcomes with emerging research suggesting stronger positive associations between positive adolescent mental health and open communication and monitoring, than with restrictions and surveillance [7,8].

(12) Study design
I would expect to see the design chosen with reference to previous work, particularly when it comes to the measures being used. For example, what mental health measures/questionnaires are being used? Why has the backwards span task been chosen (especially over other tasks)? I note also that the backwards span task mentioned in the Study Design section (Page 7, paragraph 3) are not mentioned in any detail in the Measurements section later, just a reference to previous uses.

To better justify our rationale for the study design, the following has been added to ***Methods and analysis/Study Design***:

This study employs an interrupted time series design (Figure 1), with the introduction of the Social Media Minimum Age legislation serving as the exposure. Data will be collected from parent/carer–adolescent dyads (note for parsimony the manuscript refers to parent/adolescent dyads going further, but is meant to be inclusive of other caregivers) at four timepoints: baseline (collected in the 6-weeks before the implementation of the Australian Social Media Minimum Age legislation on December 10, 2025); then again at 6-month (April-May 2026), 12-month (Oct-Nov 2026) and 18-month (April-May 2027) follow-ups.

At each timepoint adolescent participants will complete a core assessment (approximately 70 minutes) as well as a 5-day ecological momentary assessment (EMA). The EMA includes three brief surveys per day (approximately 2 minutes per survey, 30 minutes total). Parent participants will complete a core assessment at each timepoint (approximately 30 minutes).

The adolescent core assessments include a questionnaire battery that captures anxiety, depression, wellbeing and disordered eating behaviours, as well as individual dispositional factors such as social sensitivity and inclination to social comparison, with validated measures. Attitudes and experiences relating to social media will also be captured with validated measures as well as a set of bespoke questions capturing attitudes and behaviours relating to the Australian Social Media Minimum Age legislation (see Measurements below, Table S3 and Appendix 1 for specific measures and questions as they appear to participants). Cognitive functioning will be assessed using the affective backward digit span task (approximately 5 minutes), a validated measure that captures cognitive control in affective and neutral contexts [9,10]. This will allow us to examine potentially different impact of social media on affective versus cognitive control. In addition, adolescent participants with a smartphone will be asked to upload screen shots of overall time spent on their phone and on their most used applications over the last full week.

Parent core assessments will include a questionnaire battery, assessing parents' digital parenting style as well as their own and their child's social media attitudes and behaviours.

(13) Participant Sample Size and Recruitment
Having a smartphone was also an inclusion criteria for the screenshots of usage, presumably.
Any inclusion/exclusion criteria for the parents/carers should also be acknowledged.

In response to this comment, the following edits were made to the ***Methods and analysis/ Participant Sample Size and Recruitment*** section:

All adolescent and parent participants were required to be fluent in English and living in Australia. There were no other inclusion/ exclusion criteria for parents. Adolescent participants were not required to have a smartphone to participate in the questionnaire component of the study. To be eligible to participate in the EMA component of the study, adolescents were additionally required to have access to an Android or iOS smartphone for the four 5-day, multi-daily survey periods following the baseline, and 6-, 12- and 18-month assessments. These participants were then also asked to upload screenshots of screentime as a part of the questionnaire battery. Adolescent participants could earn up to $170 (questionnaires only) or $190 (questionnaires and EMAs), and parent/carers up to $55, for completing all parts of the study.

(14) It isn't clear whether there are plans to recontact/re-recruit parents or adolescents that started the baseline survey without a matching partner.

In response to this comment, the following edits were made to the ***Methods and analysis/ Participant Sample Size and Recruitment*** section:

This resulted in a sample of 422 parent and adolescent dyads being screened. Of these 375 matched parent-adolescent dyads commenced baseline. An additional 20 parents and 10 adolescents commenced the baseline assessment; however, they did not have a matching adolescent or parent, respectively, start the baseline survey. All dyads where at least one of the dyad (parent or adolescent) completed the baseline questionnaire will be contacted to complete the 6-, 12- and 18-month follow-up questionnaires.

(15) It's great that the authors have already highlighted the representativeness (or lack of) for the baseline sample. It would be good to see what the plans are to account for this in the analyses (e.g., constructing weights).

In response to this comment, the following edits were made to the ***Methods and analysis/ Data analysis/Inferential analysis*** section:

Sensitivity analyses will be conducted including socio-economic status in the analyses to investigate whether any effects hold when accounting for individual differences in socio-economic status indexed through self-perceived wealth and parental education. Each will be entered in separate sensitivity analyses. If sample attrition varies as a function of any specific characteristics, non-response weights will be applied.

Additionally, the Strengths and limitations of this study section have been edited – see response to comment (3)

(16) From table 1 it appears that most adolescents are relatively young. It may be good to also present number in age bands, as I'd imagine those aged around 15 will be of particular interest since they will span the boundary of the ban.

In response to this comment, to enable readers to see the distribution of participants by age the following edits were made to **Table 1**:

Age in years n (%)	
10	*28 (8%)*
11	*43 (12%)*
12	*73 (20%)*
13	*100 (27%)*
14	*70 (19%)*
15	*51 (14%)*
All 10-15 years M (SD)	*12.8 (1.4)*

(17) Table 2 includes the age of parents/carers, but still labels them as 'all 10-15 years' (Page 19)

Thank you. This error in Table 2 has been rectified.

(18) Patient and Public Involvement
As noted in the Ethics section, I would expect a study as topical as this to demonstrate some direct engagement with relevant stakeholders. It would make sense, for example, to give young people some say on what questions are included in the survey and EMA components, given that they are experts by experience. Currently, it sounds like the study relies on precedent and briefings, which doesn't seem sufficient.

The authors acknowledge the reviewer's concerns about PPI involvement. The broader context to this project involves a collaboration between two research groups that have embedded ongoing engagement with young people as part of our usual research activities. For example, three authors on the current paper have been involved in the Future Proofing Study, longitudinal cohort study focused on the mental health of over 6000 young people which includes a focus on screen and social media use (e.g., https://www.blackdoginstitute.org.au/wp-content/uploads/2024/08/Teens-Screens_Report-2024_WEB.pdf). For this study, we have regularly involved youth advisors to guide us on all aspects of the research, including questionnaire selection, wording of questions, and been guided by their areas of interest. Furthermore, our teams have also led the development of a digital literacy program, which is closely related to screen and social media use, which involved consultation with students across 12 schools via surveys (n=336) and focus groups (n=521) to inform the design, development and delivery of the 'Teens & Screens' program. To inform the current study, we drew upon our experience and advice from young people across these activities over the last five years.

This study has been developed in response to adolescents', parents' and policymakers' expressed needs to understand how social media use relates to adolescent mental health. Public surveys indicate that, of those concerned about young people's mental health, 44% of parents and 22% of adolescents believe social media is the thing that most impacts teen mental health [11], and they are looking for guidance about how to address this [12,13]. Further, this study design is informed by "known evidence gaps" identified as priorities by the U.S. Surgeon General including, but not limited to, the impact of online versus offline social interaction on mental health, the potential pathways through which social media causes harm, and the moderating role of development stage on the relationship between social media use and adolescent mental health [14]. Importantly, all study measures, including the questionnaires, cognitive task and EMA protocol, have been validated in adolescents (10-24-years old; Sawyer et al., 2018). The research team have regularly involved youth advisors to guide us on all aspects of the research, including consultation with students across 12 schools via surveys (n=336) and focus groups (n=521) to inform the design, development and delivery of a social media literacy program. To inform the current study, we drew upon our experience and advice from young people across these activities over the last five years.

(19) Measurements
As noted in the Study design section, what are the specifics of the backwards span task? What are the stimuli, what are the conditions, how many trials, how long is the task, is there feedback, etc.

We now provide the details of the affective backward digit span task in the ***Methods and analysis / Measurements*** section:

Cognitive Task

At the end of the adolescent questionnaires, participants will be redirected to in Gorilla to complete the affective backwards digit span task as a measure of affective and cognitive control[23]. Participants will be presented with a series of digits (1500ms) superimposed on images (Figure 3a&b). After the last image in a series they are asked to report the digits in reverse order on an on-screen number pad (Figure 3c). The task begins at a span level presenting two digits. Each span level is presented twice. The task progresses to the next span level until the participant has completed both trials at a given level incorrectly, or a span of 8 digits is reached, whichever comes first. The task is presented twice in two conditions. The presentation order is counter-balanced across participants. In one condition the images are neutral and in the other condition they are affective. All images are derived from the Geneva Affective Picture Database [45]. Three indices will be derived from this task: affective span and neutral span (cognitive control), which are operationalised as the maximum span levels achieved in the affective and neutral conditions respectively; and affective control is operationalised, as the proportional difference between affective and neutral span levels divided by the overall span level. Proportional scores are sensitive to performance differences that are accounted for by age-related differences in cognition.

(20) I appreciate the tables summarising the survey questions, but note that most readers would expect the full survey and EMA to be accessible (including logic) in the supplementary/appendix (e.g., as a PDF). While they are summarised in the tables, there is no detail about the mental health and wellbeing measures in the actual text. There should at least be mention of specific scales and the intended aim/point of each (e.g., measuring psychological distress).

In addition to the summary table, the measures have been added as appendices to a ***Supplementary Materials*** file as they appeared to participants in Qualtrics. Per BMJ Open guidelines, an additional "***Supplementary Material***" section has been added to the main manuscript outlining this:

Supplementary Materials

The following are available in the Supplementary Materials:

- *Baseline adolescent (Table S1) and parent (Table S2) demographics*
- *Summaries of the adolescent (Table S3), parent (Table 34) and EMA (Table S5) questionnaires including categories, constructs, measures and sources*
- *Example images from the cognitive task (Figure S1)*
- *Baseline adolescent (Appendix 1) and parent (Appendix 2) questionnaire batteries as they appear in Qualtrics*
- *Adolescent EMA survey as it appears in Qualtrics*

Additionally, the ***Methods and analysis / Measurements / Questionnaires*** section has been replaced with a more detailed and specific description of the measures and their purpose:

Participant demographics. *Age, gender and ethnicity were captured for parents and adolescents. Parents additionally reported on country of residence, postcode, gender, ethnicity, level of education and perceived wealth*

Adolescent mental health. *Parents report whether adolescents have previously and/or currently received any mental health or neurodevelopmental diagnoses and/or status. Adolescent completed self-report measures to capture their symptoms of mental health problems and mental wellbeing over the past two weeks. Adolescent generalized anxiety symptoms will be measured using the 7-item Generalized Anxiety Disorder Scale (GAD-7) [30] with items such as "Not being able to stop or control worrying" and "Trouble relaxing". Adolescent depression symptoms will be assessed using the Patient Health Questionnaire-Adolescent, 8-Item Version (PHQ-8) [31–33] with items such as "Little interest or pleasure in doing things" and "Feeling down, depressed, irritable or hopeless". Both the GAD-7 and PHQ-8 items are reported on a 4-point Likert scale from "Not at All" to "Nearly Every day". Wellbeing will be assessed using the Short Warwick-Edinburgh Mental Well-being Scale[34], with items such as "I've been feeling useful" and "I've been thinking clearly", assessed on a 5-point Likert scale from "None of the Time" to All of the Time". Eating disorder symptoms will be measured using the 8-item Eating Disorder Examination Questionnaire adapted for Children [35] with items such as "Have you tried not to eat any foods you like to control your weight and shape?" and "How often have you felt guilty after*

eating because of the effect on your shape and weight?". These scales are well validated and commonly used, enabling comparison across previous and future studies.

Adolescent individual differences. *As a key developmental vulnerability factor, social sensitivity will be measured using the Online and Offline Social Sensitivity Scale [37]. The 18-items scale captures participants sensitivity to social rejection with both general and online specific items such as "I always expect criticism" and "I delete my social media post if I don't get the responses I wanted". A social version of the Repetitive Negative Thinking Questionnaire (RNTQ) will be used. The original questionnaire assesses repetitive negative thinking tendencies across generic negative situations; in this modified version the situations are adapted to be social (e.g. "Following unpleasant social interactions my thoughts keep repeating themselves."). This 8-item social version has been validated in older adolescents (Bilston & Schweizer, 2024). This questionnaire will be validated for younger adolescents in this study. Additionally, as a lot of concern around social media use focuses on the displacement of healthy habits, physical activity will be assessed using a 2-item measure assessing physical exercise time per day (using a validated single item measure [38]) and days per week (selected from the International Physical Activity Questionnaire - Short Form [39]). Also sleep quality will be assessed using the 8-Item PROMIS Pediatric Sleep Disturbance Form [40]. All these measures are validated in adolescent populations.*

Adolescent and parent social media attitudes and behaviors. *Adolescent internet and social media usage including access, behaviour, and parental oversight will be assessed using questions from the LSE Toolkit [41] as well as bespoke questions regarding age of first phone, social media and messaging use. Adolescent and parent attitudes to social media will be assessed using the Social Media Mindsets Scale [42], which includes both valence and agency items such as "Using social media is meaningful for me" and "I'm in control of how I use social media" respectively, measured on a 5-point Likert scale from "Strongly Disagree" to "Strongly Agree". An adapted version of this scale to assess parents' attitudes to their child's social media use. Parent's approach to managing and monitoring their child's social media use will be assessed using the Digital Parenting Scale [13] which includes items such as "I have rules about the amount of time my child is allowed to spend on digital devices" and "I teach my child how to behave on digital devices" measured on a 5-point Likert scale from "Never" to "Always".*

Mechanisms of social media impacts. *Social comparison will be assessed using the Social Comparison Scale [43], with 10 items such as "Unlikeable" to "More Likeable" and "Untalented" to "More Talented" reported on a 10-point semantic differential scale. Specific appearance-based comparison will also be assessed using a subscale of the State Self-Esteem Scale [44] with items such as "I am dissatisfied with my weight" and "I feel good about myself" measured on a 5-point Likert scale from "Not at all" to "Extremely". Online harm exposure and responses will be assessed using five items (contact with strangers, bullying, sexual content, violence and hate messaging, and actions without consent) from the EU Kids Online Research Toolkit. Examples of questions include "Have you ever had contact on social media or messaging with someone you or your family/a close friend have not met face-to-face before?" and "In the past year, have you ever seen any sexual images (not including via school or home education on puberty and sexuality)?*

Australian Social Media Minimum Age Legislation Impact. *Australian adolescent and parent changes in social media use associated with the ban will be assessed using bespoke items asking about knowledge about the ban and reactions to publicity before (at baseline), and after the introduction of the ban (at 6-, 12- and 18-month follow-ups).*

Screen Shot Uploads. *Objectively measured total phone and online social interaction time for the prior week will be measured via screen time reports uploaded and extracted at each timepoint.*

<u>(21) Data analysis</u>
Again, I would encourage the authors to think about producing sample weights, given the representativeness issue but also likely drop-out longitudinally. This would benefit any reuse of the cohort in future, too.

We thank the reviewer for their suggestion. Instead of weighting the cross-sectional analyses we will conduct sensitivity analyses including sociodemographic status as described above. However, in response to the reviewer, we will apply non-response weights to the longitudinal data. Sample weighting has now been described in the ***Methods and analysis / Participant Sample Size and Recruitment*** section as follows:

Sensitivity analyses will be conducted including socio-economic status in the analyses to investigate whether any effects hold when accounting for individual differences in socio-economic status indexed through self-perceived wealth and parental education. Each will be entered in separate sensitivity analyses. If sample attrition varies as a function of any specific characteristics, non-response weights will be applied.

(22) It would be good to see a little more detail on the inferential analysis, or at least an example. What is the most important/basic model for assessing the interrupted time series? Is the SEM part intended to help boil-down the various questionnaires and scales into something more latent, if so what? Similarly, it would be good to identify any confounders (e.g., parental education, source of recruitment) that would be important to account for in analyses.

We now further detail our analytic approach. Please note we have removed reference to dynamic structural equation modelling as our sample size is inadequately powered to conduct these analyses with the limited number of data points. We have revised the ***Methods and analysis / Inferential analyses*** section as follows:

Data collected throughout the study will be analysed using multiple methods including linear regression and mixed-effects models will be applied to questionnaire and EMA-derived data, for cross-sectional and prospective analyses respectively. These analyses will enable assessment of mental health, wellbeing and cognitive outcomes between individuals over time, in relation to individual demographic, dispositional, developmental and experiential differences, digital parenting approaches and governmental policy adherence. The repeated measures and longitudinal design will also enable examination of changes within individuals based on changes in their online and offline social interaction behaviours over time. The core analyses will include separate models for each set of outcomes of interest: mental health, cognition and wellbeing. The models will include pre/post legislation and Wave (baseline, 6m, 12m, 18m) as fixed effects and participant ID as random intercept effect. Changes in the proposed mechanisms of action (e.g., harms exposure) will be added to these models to examine whether they account for any changes in the outcomes of interest before and after the Social Media Minimum Age legislation. Additionally, mixed-effects segmented regression will be applied to EMA-derived data to examine trends in momentary mood before and after the Social Media Minimum Age legislation. This will allow us to model mood trends across the 15 EMAs before the Social Media Minimum Age legislation to each of the EMAs after the legislation. The inclusion of Time point as nested variable will allow us to track the differential impact over time.

(23) Ethics and safety considerations
I would like to see some mention of the safety of the data, too, and the need to minimise the risk of identification. How will the authors ensure that this very personal data cannot be used to reidentify potentially vulnerable adolescents?

We agree that data security is paramount and have added the following paragraph in the ***Ethics and dissemination / Ethics and safety considerations*** section:

Additionally, data privacy and confidentiality are of utmost importance to protect this vulnerable population. Data will be stored on a secure UNSW OneDrive server, with access restricted to the research team. During the study, data will be stored in a re-identifiable format where any identifiers removed and replaced with a unique code for each participant as well as a dyad code for linking parents and adolescents, and a family code for linking parents and siblings. A linkage file including the personally identifiable email, phone number, and random participant ID will be stored separately on UNSW OneDrive only accessible by the Chief Investigator and Lead Researcher. Data will only be re-identified for in the case of withdrawal.

(24) Dissemination of findings
I would also expect some plan for data accessibility. Will the data be accessible to other researchers, if so will it be deidentified (has it been consented), where will it be deposited, etc.?

Deidentified data and all analyses will be made available via the Open Science Framework. The following has been added to the manuscript in ***Ethics and dissemination / Dissemination of findings*** section articulating this:

The results of this study will be disseminated through multiple channels including academic journals, conference presentations, and researcher related websites. Participant confidentiality will be maintained by

reporting only aggregate results and not including any individually identifying information in publications. Participants who would like to receive the study results will be able to provide their email or postal address to the research team who will share aggregated findings only. Additionally, included participants have provided consent to de-identified information being shared for use in future research in an open access repository that will also include all analysis and measures used in the study: https://osf.io/thky7/overview.

VERSION 2 - REVIEW

**Reviewer**	**1**
**Name**	**Iveson, Matthew**
**Affiliation**	**University of Edinburgh, Department of Psychology**
**Date**	**26-Jun-2026**
**COI**	

I would like to thank the authors for their perseverance in revising the manuscript. Given the topical nature and potential impact of the study, I believe it is best to be clear and thorough. The authors have added substantial detail to the protocol, adding to the clarity of its aims and methods. I particularly appreciate the details about the tasks, planned analysis and previous stakeholder engagement. I hope the authors continue this engagement and commitment to open science throughout the study. My only remaining recommendation it to have a read-through of the final manuscript, as there are a few typos (e.g., "redirected to in Gorilla to complete…", Page 10, line 6; "re-identified for in the case of", Page 12, line 16) from the new additions that should be easily rectified before publication.
